# Natural circularly permuted group II introns in bacteria produce RNA circles

**DOI:** 10.1016/j.isci.2021.103431

**Published:** 2021-11-13

**Authors:** Adam Roth, Zasha Weinberg, Koen Vanderschuren, Mitchell H. Murdock, Ronald R. Breaker

**Affiliations:** 1 Department of Molecular, Cellular and Developmental Biology, Yale University, New Haven, CT 06520-8103, USA; 2 Howard Hughes Medical Institute, Yale University, New Haven, CT 06520-8103, USA; 3 Department of Molecular Biophysics and Biochemistry, Yale University, New Haven, CT 06520-8103, USA

**Keywords:** Biological sciences, Molecular biology, Structural biology, Bioinformatics

## Abstract

Group II self-splicing introns are large structured RNAs that remove themselves from transcripts while simultaneously sealing the resulting gaps. Some representatives can subsequently reverse splice into DNA, accounting for their pervasive distribution in bacteria. The catalytically active tertiary structure of each group II intron is assembled from six domains that are arranged in a conserved order. Here, we report structural isomers of group II introns, called CP group II ribozymes, wherein the characteristic order of domains has been altered. Domains five and six, which normally reside at the 3′ end of group II introns, instead occupy the 5′ end to form circularly permuted variants. These unusual group II intron derivatives are catalytically active and generate large linear branched and small circular RNAs, reaction products that are markedly different from those generated by canonical group II introns. The biological role of CP group II ribozymes is currently unknown.

## Highlights

• Circularly permuted versions of group II introns occur in multiple bacterial phyla
• The splice sites are reversed in order and located within the interior of the ribozyme
• CP group II ribozymes generate large branched and small circular RNA products *in vitro*
• The biological function of this ribozyme class is not known

## Graphical abstract

**Figure:**
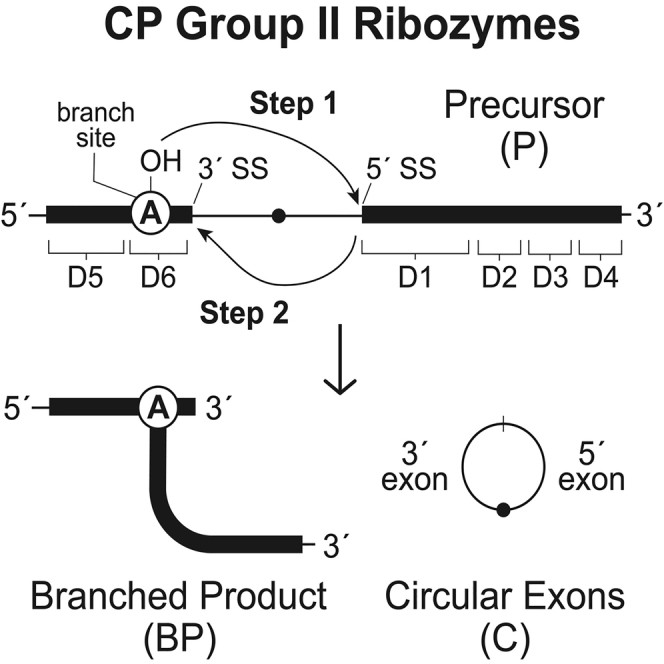

## Introduction

Catalytic RNA molecules participate in a great diversity of biological processes, including ribozyme-mediated regulation of gene expression, protein synthesis, and RNA modification (Cech and Steitz, 2014). Despite this spectrum of prominent roles, there are very few large catalytic RNAs known to exist in modern cells, probably because those present in the ancient RNA world (Benner et al., 1989; Robertson and Joyce, 2012) have long ago been driven to extinction by competitive pressure from protein-based enzymes. Nonetheless, recent reports have identified additional classes of large structured RNAs in bacteria (Puerta-Fernandez et al., 2006; Weinberg et al., 2009, 2017), some of which are comparable in size and structural complexity to known large catalytic RNAs. This highlights the tantalizing possibility that additional classes of large ribozymes may yet persist in some species and await discovery.

Some contemporary large ribozymes, such as group I and group II self-splicing introns (Lambowitz and Belfort, 1993), have probably solidified their place in modern metabolism by virtue of their roles as specialized selfish genetic elements. One of the survival strategies executed by these RNAs is to catalyze auto-excision from mRNAs. This keeps adverse effects on their host organisms to a minimum while simultaneously providing access to a wider variety of genomic insertion sites.

To catalyze its own removal from RNA transcripts, each group II intron forms an elaborate, conserved secondary structure typically consisting of six domains arranged in a characteristic order (Michel et al., 1989). In a test tube, some representatives can promote the successive phosphoester transfer reactions that constitute splicing without the aid of protein factors. In a biological setting, however, group II ribozyme activity is augmented by conformational adjustments made through association with an intron-encoded protein (IEP) (Wank et al., 1999; Haack et al., 2019). The IEP, whose open reading frame (ORF) is sequestered in one of the intron domains so as not to impede catalysis, can exhibit multiple activities, including those corresponding to reverse transcriptase and DNA endonuclease (Zimmerly et al., 2001). Group II introns commonly require a ‘branchpoint adenosine’ in domain six (D6), which presents its 2′ oxygen atom as the nucleophile for the first phosphoester transfer reaction involving the 5′ splice site (SS). The newly formed 3′ hydroxyl group subsequently presents its oxygen atom as the nucleophile for attack at the 3′ SS in the second phosphoester transfer reaction. This two-step process results in ligated exons and an excised intron that exists as a lariat containing a 2′-5′ phosphodiester linkage (van der Veen et al., 1986; Zimmerly and Semper, 2015). Subsequent to these splicing reactions, the IEP remains associated with the lariat so that it can assist with the next phase of the group II intron replication cycle (Haack et al., 2019): reverse splicing into DNA where, once it has been reverse transcribed, the group II intron is poised to propagate once again. Employing this strategy, group II introns have been highly successful, promoting their phylogenetic dispersal and spawning ribozyme evolutionary descendants that include spliceosomes found in many eukaryotic species (Fica et al., 2013).

As might be expected for a widely distributed mobile genetic element, a number of structural variants of group II introns have been observed. Some of these variants have missing or nonfunctional domains within the IEP (Zimmerly et al., 2001; Simon et al., 2008), whereas others lack certain domains of the ribozyme itself (Copertino and Hallick, 1993). Such variants can be functionally impaired or might depend upon the assistance of other RNAs that function in *trans* to promote splicing or mobility. There are also versions of group II introns with extra RNA domains and others that make use of alternative splice sites (McNeil et al., 2014; Tourasse et al., 2010). Yet no naturally occurring variant of the group II intron has been identified in which the 5′ to 3′ order of its major structural domains has been scrambled.

Here we describe the identification of a collection of unusual variants of group II introns in bacteria, originally called “T-large” RNAs (Harris and Breaker, 2018). Encoded within chromosomes and on plasmids in multiple phyla, this RNA averages approximately 900 nucleotides (nt) in length and bears a striking resemblance to group II self-splicing introns (Lambowitz and Zimmerly, 2011), but for two important distinctions. First, unlike group II introns, which contain six major structural domains (D1 through D6) whose order of occurrence is universally conserved (Michel et al., 1989; Zimmerly and Semper, 2015), the newly identified RNAs contain analogous domains arranged in an alternative order, such that the D5 and D6 elements typically residing at the 3′ end of group II ribozymes occupy a new position at the 5′ end of this variant RNA class. Second, because the locations of the splice sites are maintained with respect to their immediately adjacent domains, the splice sites occur in reverse order in the rearranged group II variant and are positioned in the interior of the ribozyme rather than at the intron boundaries. We find that the circularly permuted variants of group II ribozymes mediate reaction chemistry identical to that of standard group II introns but, owing to their shuffled domain order, generate reaction products that are drastically different. It remains to be determined what biological role is fulfilled by the action of this structural variant of the group II ribozyme.

## Results

### Identification of a large noncoding RNA resembling group II introns

**Figure 1:**
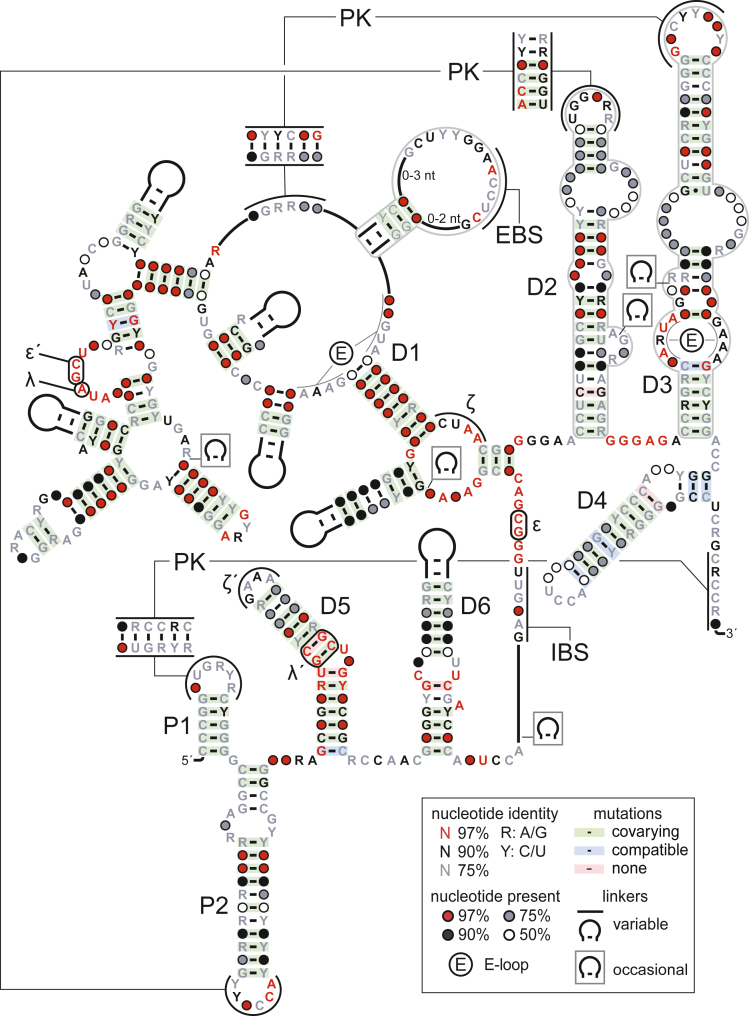
Identification of a large RNA resembling the group II self-splicing intron Consensus sequence and secondary structure model of CP group II RNA. Proposed pseudoknot (PK) interactions unique to CP group II RNAs are indicated. With the exception of P1 and P2, which are also unique to CP group II RNA, individual domains and predicted tertiary interactions (indicated by Greek letters or by EBS [exon-binding site]/IBS [intron-binding site]) adopt the nomenclature of the structurally analogous elements in group II introns. Multiple secondary structure architectures are observed in the positions occupied by the domains or subdomains outlined in gray. See also Figures S1–S4.

As part of a recently published search for structured RNAs using methods based on comparative genomics (Weinberg et al., 2017), we identified a ∼900-nucleotide noncoding RNA (ncRNA) class with extensive secondary structure that is encoded in bacterial chromosomes and plasmids (Figure 1). These RNAs were originally detected by analyzing sequences from a hot spring metagenome (Swingley et al., 2012). Examples were later found in bacterial species within *Thermus*, a genus in the phylum Deinococcus-Thermus, as well as in assorted organisms within the phyla Firmicutes and Proteobacteria (Data S1). In total, our searches revealed ∼290 non-redundant sequences corresponding to this large ncRNA class.

Searches for related RNAs matched some fragments of group II introns that were predicted using models in the Rfam database (Gardner et al., 2009). In addition, manual analysis revealed that specific substructures of the newly found RNAs bear striking resemblances to some domains that define the group II intron class, as outlined in more detail below.

Despite the apparent correspondence of the individual component domains between the two RNA classes, the order in which these structural elements occur in the newly discovered class is distinct from the highly conserved domain order observed in group II introns (Figure 1). In the variant RNA class, domains that are highly similar to D5 and D6 reside near the 5′ end. This is in stark contrast to all previously known group II introns, where these domains occupy positions toward the 3′ end. Furthermore, domains that appear to be analogous to D1, D2, D3, and D4 of group II introns occur 3′ to the D5- and D6-like substructures in the newly identified RNA collection. The resulting domain order of this RNA class (D5-D6-D1-D2-D3-D4) thus represents a substantial rearrangement of canonical group II intron primary structure (D1-D2-D3-D4-D5-D6). Due to its similarities to group II ribozymes and the characteristic altered domain order, we recommend this collection of large noncoding RNAs be designated as the circularly permuted (CP) group II RNA class.

### Similarities between CP group II RNAs and typical group II ribozymes

**Figure 2:**
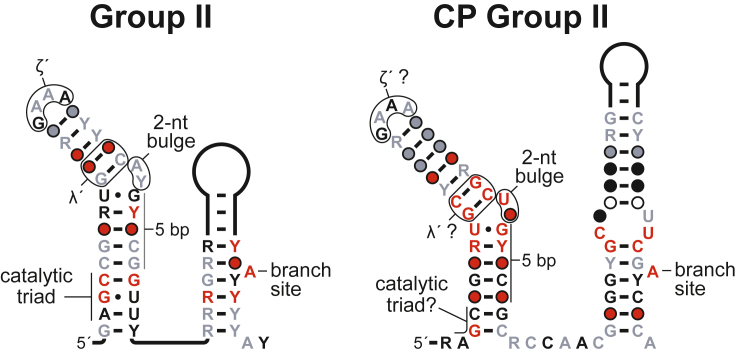
Comparison of D5 and D6 in canonical and permuted group II ribozymes Consensus sequence and secondary structure models of D5 and D6 from group II and CP group II RNAs. Potentially shared features of these structures are indicated. Other annotations are as described in Figure 1.

Among group II introns, D5 and D6 display the highest level of sequence and structure conservation. Therefore, the occurrence of comparable domains within CP group II RNAs was strongly suggestive of both evolutionary and functional relationships to canonical group II introns. Indeed, D5 and D6 of CP group II RNAs exhibit sequence and secondary structure features that are highly similar to those of the analogous domains in canonical group II introns (Figure 2). D5 is the catalytic center of group II ribozymes, positioning two mechanistically critical divalent metal ions via interactions between the highly conserved AGC “catalytic triad” at the base of D5 and the AY bulge forming the elbow of this domain (Figure 2) (Toor et al., 2008). The start of D5 in CP group II RNAs possesses the same trinucleotide sequence (albeit with an adenosine apparently not participating in a canonical Watson-Crick pair), which potentially could interact with the UN bulge in D5 to coordinate divalent metal ions in an analogous fashion. Moreover, the identical number of base-pairs (five) separating these regions in CP and canonical group II introns would permit a similar mode of interaction (Figure 2).

The terminal loop and stem regions of D5 are known to mediate long-range tertiary interactions with D1 that are critical for choreographing the active site conformations required for group II intron splicing (Pyle, 2016). Due to the high degree of conservation of D5 in group II introns and CP group II RNAs, it is possible from sequence and positional similarities alone to make predictions regarding some of the putative corresponding elements in CP group II RNAs. For example, by analogy with ζ-ζ′ interactions in group II introns (Candales et al., 2012), the D5 tetraloop in CP group II RNA is likely to dock with conserved nucleotides of the three-stem junction at the base of D1 (Figures 1 and 2). Comparison with canonical self-splicing introns (Boudvillain et al., 2000) also suggests that a non-Watson-Crick interaction between the λ' site in D5 and an adenosine residue within a highly conserved hexanucleotide sequence in D1 is also present in CP group II RNAs (Figures 1 and 2).

Similar comparisons can be made elsewhere in the RNA. The key feature of D6, the bulged adenosine that serves as the nucleophile in the first step of group II intron splicing, is also conserved in CP group II RNAs (Figure 2). In addition, there is an internal loop within this domain in the permuted RNA that contains conserved nucleotides (Figure 2), which is consistent with the presence of an internal loop at this position among some group II introns (Candales et al., 2012; Chu et al., 1998; Toor et al., 2001).

Downstream of D6 in CP group II RNAs is a large domain, displaying an intricate secondary structure that is the likely analog of D1 in group II ribozymes (Figure 1). In the canonical group II intron, D1 serves as a structural scaffold by forming long-range tertiary contacts with portions of the active site and also contains exon-binding sites (EBS) that participate in SS selection via base-pairing (Pyle, 2016; Michel and Ferat, 1995). There is a variable-length linker sequence between D6 and D1 in CP group II RNAs, which is bounded by highly conserved segments that resemble the consensus nucleotides demarcating the 5′- and -3′-splice sites (GUGYG and AY, respectively) (Zimmerly and Semper, 2015) of canonical group II introns (Figure 1). In CP group II RNAs, these “boundary” segments reside in the expected positions relative to D1 and D6, respectively, but are proximal to one another, separated only by the linker sequence. Thus, these splice sites occur in reverse order in the primary structure and are positioned wholly within the body of the CP group II RNA rather than at the 5′ and 3′ boundaries of the typical string of group II intron domains. Interestingly, the ε-ε′ long-range base-pairing interaction that in group II introns guides the selection of the 5′ SS (Jacquier and Michel, 1990) also appears to exist in CP group II RNAs (Figure 1).

Immediately 3′ to D1 in CP group II RNA are two domains that probably correspond to D2 and D3 of group II ribozymes. D2 and D3 are smaller domains that provide structural contributions to the splicing reaction, respectively helping to appropriately position D6 and accelerating reaction chemistry through an array of interactions with D5 and the 5′ SS in D1 (Costa et al., 1997; Fedorova and Pyle, 2008). The purine-rich intervening sequence (J2/3), which makes important long-range contacts with D5 (Toor et al., 2008; Podar et al., 1998), is a feature shared by both group II and CP group II RNAs, as is the E-loop at the base of D3 (Figure 1) (Lambowitz and Zimmerly, 2011). Also, like the canonical group II intron, CP group II RNA tolerates a certain degree of structural variety in D2 and D3, as evidenced by the multiple secondary structure architectures that are accommodated at these positions (Figures S1 and S2).

Based on its positional relationship to D1, D2, and D3, the stem-loop near the 3′ end of CP group II RNA is referred to as D4. In group II ribozymes, the primary role of D4 is to accommodate the ORF encoding the IEP, although subdomains within D4 also serve to promote the association of the IEP with the ribozyme, enhancing both splicing of the intron and retrotransposition of the free lariat (Lambowitz and Belfort, 2015). However, no ORFs have been observed within the predicted boundaries of CP group II RNAs, and so the D4 assignation is based on its relative position within CP group II RNA rather than on its predicted analogous function.

Group II introns rely on several intra- and inter-domain long-range base-pairing interactions to establish their active conformations (Michel and Ferat, 1995; Pyle, 2010). Likely related to domain reordering, CP group II RNAs contain a unique set of predicted pseudoknots. The terminal loop of D3 is proposed to dock with a D1-embedded sequence through Watson-Crick base-pairing, and the P1 loop near the 5′ end of the RNA potentially forms a pseudoknot with a segment at the 3′ end (Figure 1). In addition, the D2 terminal loop is predicted to form a pseudoknot with the terminal loop of the P2 stem. In canonical group II introns, D2 is engaged in a network of interactions with D1, D3 and D6, and helps to correctly position the branchpoint adenosine of D6 in the active site (Costa et al., 1997; Robart et al., 2014). Thus, it could be that the proposed pseudoknot between P2 and D2 of the permuted variant helps to maintain an analogous spatial organization among these various domains. Overall, it seems possible that these pseudoknots behave as structural staples to assist the folding of domains whose spatial interrelationships have changed drastically in the permuted RNA. Another structural element that is unique to CP group II RNAs is a moderately conserved stem-loop residing within the intervening sequence between D6 and D1 (Figure 1; Data S1). Conceivably, this stem-loop could participate in stabilizing interactions between the permuted structural domains and the unusually situated putative splice sites.

### A proposed reaction pathway for CP group II RNA self-processing

**Figure 3:**
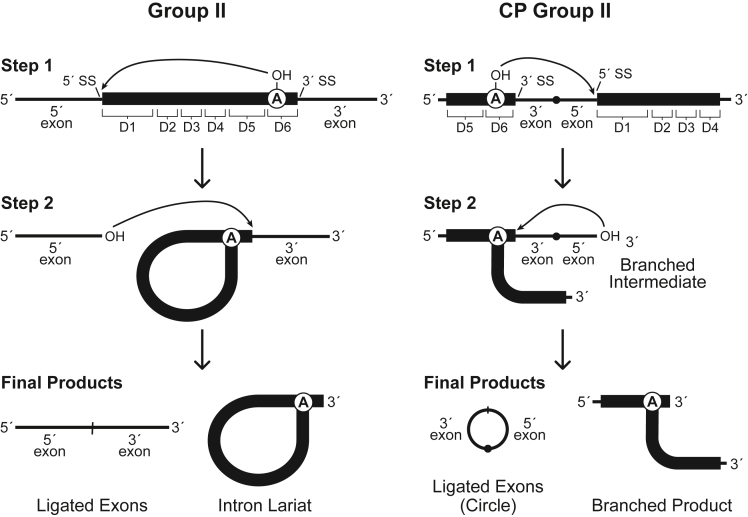
Splicing pathways for group II and CP group II RNAs The typical splicing pathway for group II introns (left) involves two steps. In step 1, the branch-site adenosine 2′ oxygen carries out a nucleophilic attack on the phosphorus at the 5′ splice site. In step 2, the 3′ oxygen at the newly generated terminus of the 5′ exon carries out a nucleophilic attack on the 3′ splice site. These successive phosphoester transfer reactions yield linear ligated exons and an intron lariat structure formed by a 2′-5′-phosphodiester linkage at the branch site. An analogous reaction pathway is proposed for the CP group II RNA (right). However, the circularly permuted arrangement of domains and splice sites in CP group II RNAs yields distinct circular ligated exons and a T-shaped branched product. There is no formal boundary between the annotated 3′ and 5′ exons in CP group II RNA, as these segments are contiguous in both precursor and product RNAs.

Given the extensive similarities between the respective structural domains of group II ribozymes and CP group II RNAs, it seemed likely that the latter would also exhibit catalytic activity by assembling an active site highly similar or identical to that of group II introns, despite permutation of the domain order. For the sake of consistency, and to simplify the comparison between canonical and CP group II RNAs, the putative splice sites in CP group II RNA are designated according to their structurally analogous sites in group II introns (Figure 3). Splice sites in group II ribozymes reside just outside of the terminal domains D1 and D6, allowing for the excision of the intact mobile RNA without any loss of information (Figure 3, left). In contrast, CP group II RNAs are arranged such that D6 and D1 occur successively within the interior of the noncoding RNA structure, and therefore the putative 5′ and 3′ splice sites are reversed in order and separated only by a short linker sequence (Figure 3, right). If the reaction mechanism proceeds analogously to that of canonical group II introns, it would yield substantially different reaction products.

Specifically, in step one of the theorized CP group II ribozyme reaction, the 2′ oxygen of the branch-site adenosine participates in nucleophilic attack on the scissile phosphate at the 5′ SS, now located downstream of the branch site (Figure 3, right). In the second step, the oxygen atom of the newly formed 3′ hydroxyl group would be expected to attack the 3′ splice site, which occurs upstream in the permuted variant. This unusual configuration is expected to generate a small covalently closed circle analogous to the conjoined linear exons produced by canonical group II ribozymes. The other, larger product of the proposed CP group II ribozyme reaction is a branched RNA, analogous to the excised lariat intron generated by typical group II introns (Figure 3, left). Interestingly, decades ago, an artificial group II intron derivative that was engineered in the laboratory (Jarrell, 1993) to have a permuted architecture like the one observed in CP group II RNAs was found to generate unusual splicing reaction products precisely like those described above.

### CP group II RNA undergoes self-processing *in vitro*

**Figure 4:**
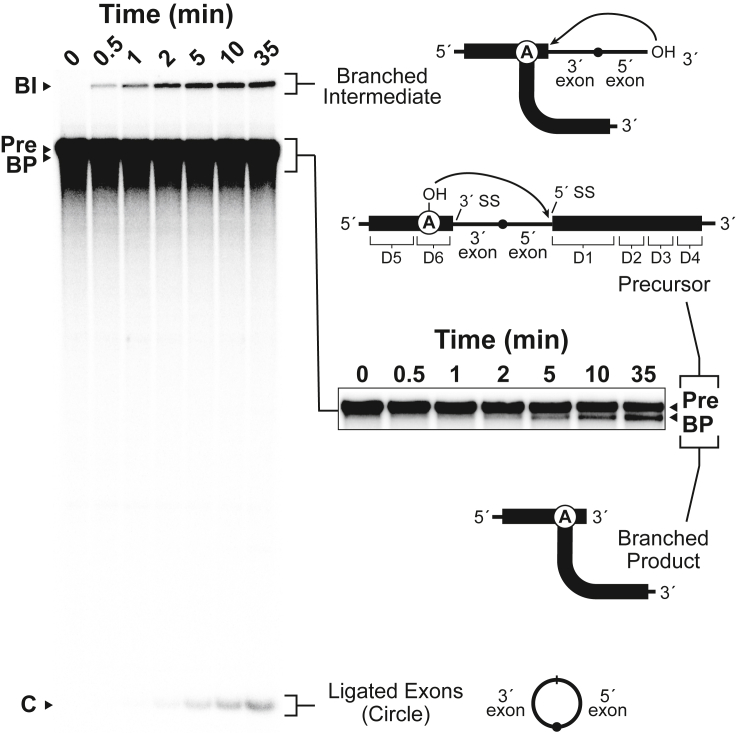
CP group II RNA exhibits RNA processing activity Products generated during a time course reaction using internally ^32^P-labeled CP group II RNA from *C. testosteroni* were separated by denaturing 7% polyacrylamide gel electrophoresis (PAGE). The precursor (Pre; 902 nt) and the product RNAs, which are predicted to include the branched intermediate (BI), the branched product (BP) and the RNA circle (C), are indicated and depicted schematically using the corresponding diagrams from Figure 3. The region of the gel encompassing the Pre and BP bands is expanded and shown with reduced exposure (inset) to allow visualization of the distinct bands. See also Figure S5.

To determine whether the natural permuted group II RNA behaves as predicted, we performed a reaction time course with an internally ^32^P-labeled CP group II RNA from *Comamonas testosteroni* KF-1. This construct, designated *Cte* 1, yields an accumulation of processed RNAs with electrophoretic mobilities (Figure 4) that are consistent with the predicted products (Figure 3). Due to its branched structure (presumably containing a 2′-5′-phosphodiester linkage), the branched intermediate (BI) formed by the step 1 reaction migrates more slowly than the linear CP group II precursor (Pre) RNA. The expected products resulting from the second step of the postulated reaction are the shortened branched product (BP), which migrates only slightly more quickly than the precursor, and the excised intervening sequence between D6 and D1, which presumably exists in circular form (C). The detection of bands potentially corresponding to the predicted reaction products indicates that this circularly permuted version of the group II intron retains autocatalytic activity, thereby constituting a variant form of the widespread class of group II ribozymes.

**Figure 5:**
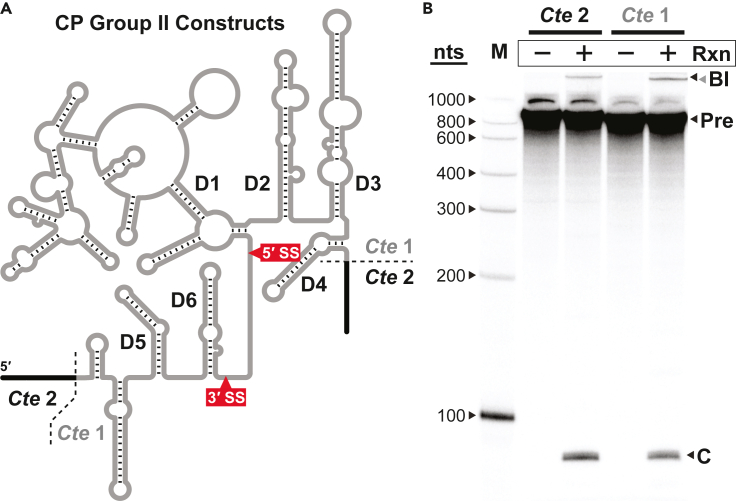
CP group II precursor RNAs of different lengths generate small products of the same size (A) Schematic secondary structure diagram corresponding to the *Cte* 1 and *Cte* 2 constructs. *Cte* 2 contains 5′ (36 nt) and 3′ (43 nt) extensions (black lines) that are not present in *Cte1*. Approximate locations of the 3′ and 5′ splice sites (SS) are indicated. (B) Internally ^32^P-labeled RNA constructs that were unreacted (−) or reacted (+) for 2 h at 23°C were analyzed by denaturing 7% PAGE. Numbers indicate the lengths in nucleotides (nts) of the corresponding RNA size markers. The gel mobility of the branched intermediate (BI) for *Cte* 1 (gray arrowhead) is faster than that of the BI for *Cte* 2 (black arrowhead). Other annotations are as described for Figure 4.

To evaluate further whether these reaction products (Figure 4) correspond to those predicted (Figure 3), we prepared a second *C. testosteroni* construct, *Cte* 2, in which additional sequences, native but presumably extraneous, were included on the ends of the CP group II RNA precursor. *Cte* 2 is 79 nucleotides (nt) longer than *Cte* 1, with an additional 36 and 43 nt at its 5′ and 3′ ends, respectively (Figure 5A). As expected, the putative branched intermediate that accumulates during the *Cte* 2 reaction exhibits slightly slower gel mobility (larger size) compared to the analogous RNA from the *Cte* 1 reaction (Figure 5B). In contrast, the short, putatively circular ligated exon products generated by *Cte* 1 and *Cte* 2 reactions co-migrate, supporting the conclusion that they indeed correspond to the intervening sequence between D6 and D1. Because this sequence is derived from the interior of the CP group II ribozyme, its size is unaffected by the 5′ and 3′ extensions in the precursor RNA. Furthermore, if the splice sites corresponding to this excised fragment are designated by sequence motifs (GGGCG and NU) in the linker joining D6 and D1 (Figure 1) that resemble the 5′ and 3′ ends of canonical group II introns (GUGYG and AY), then the length of the excised product in the *C. testosteroni* constructs should be 86 nt, which is roughly consistent with its mobility during PAGE (Figure 5B). However, the length of a circular RNA cannot be confirmed using linear RNA size markers due to the difference in PAGE mobility of circular and linear molecules.

### Characterization of CP group II RNA major reaction products

**Figure 6:**
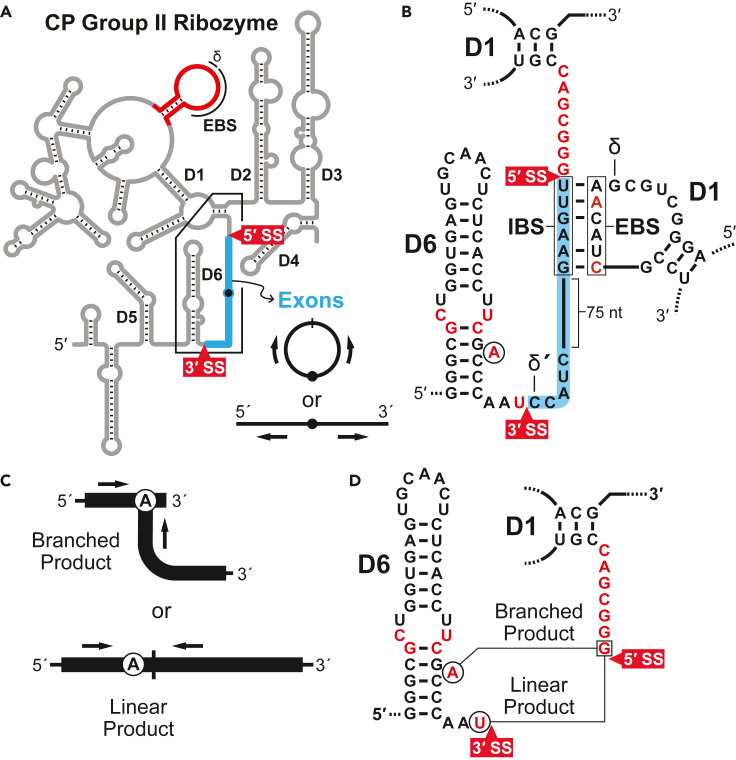
Characterization of CP group II RNA reaction products (A) Schematic depiction of possible linear and circular forms of the portion of CP group II ribozymes that is excised (blue line). Primers (arrows) were designed so that RT-PCR amplification would be productive with a circular template but nonproductive with a linear one. (B) Detailed secondary structures corresponding to regions of the *C. testosteroni* CP group II precursor RNA containing the intervening sequence between D6 and D1 (blue line) and the subdomain within D1 ((A), red line) that is predicted to participate in recognition of the 5′ splice site via base-pairing between its EBS and the IBS at the 3′ end of the 5′ exon. An additional possible base-pair that would be analogous to the δ-δ′ interaction between D1 and the start of the 3′ exon in some group II introns is also indicated. Nucleotides depicted in red are at least 97% conserved among CP group II RNA representatives. The branchpoint adenosine is encircled. The region highlighted in blue represents the sequence corresponding to the excised RNA (analogous to the 3′ and 5′ exons). (C) Schematic depictions of the branched reaction product (top) predicted to result from nucleophilic attack of the branchpoint adenosine 2′ oxygen at the 5′ splice site, and a possible linear product (bottom) resulting from a mechanism analogous to the group II intron circularization pathway. RT-PCR using primers (arrows) targeting sequences on opposite sides of the branch-site adenosine was used to define the junction between the D5-D6 and D1-D4 fragments. (D) Secondary structure diagram depicting the junctions between D5-D6 and D1-D4 fragments that possibly correspond to different reaction products generated during CP group II RNA catalysis. Junctions occur between the boxed nucleotide and either of the circled nucleotides, with thin lines representing zero-length connectors. Other annotations are as described for (B). See also Figure S6 and Table S1.

We next sought to determine the exact sequence and length of the small excised product and whether it exists in circular or linear form. Using as a template the gel-purified RNA product corresponding to band C (Figure 4) from a reaction with *Cte* 1 RNA, we performed reverse transcription followed by PCR (RT-PCR). We utilized a DNA primer pair that targeted the isolated RNA, but that would fail to generate an exponential amplification product if the RNA target was linear. With a linear RNA template, the primers would be oriented divergently (Figure 6A), resulting in a nonproductive RT-PCR reaction. In contrast, if the RNA target was circular, the same primer pair would be convergently oriented, thereby yielding robust PCR amplification. More specifically, given a circular RNA template, reverse transcriptase would generate cDNAs corresponding to multimers of the excised sequence. The subsequent PCR step would then be expected to generate multiple amplification products of various lengths corresponding to these tandem repeats, due to the numerous primer-binding sites on the multimeric cDNA template.

Analysis of the RT-PCR products by agarose gel electrophoresis revealed multiple amplified products (data not shown), consistent with a circular RNA template. This heterogeneously sized population of double-stranded DNAs was then subcloned and subjected to DNA sequencing, revealing that the PCR products contained multimers of a unit-length sequence that corresponds precisely to the linker region between D6 and D1 in *C. testosteroni* CP group II RNAs (Table S1). The nucleotides flanking this linker that correspond to the conserved nucleotides defining the putative 3′ (NU) and 5′ (GGGCG) splice sites (Figure 6B) are not included in the circularized RNA product generated by CP group II RNAs, which is consistent with the exclusion of the analogous sequences from the ligated exons of canonical group II ribozymes (Lambowitz and Zimmerly, 2011; Pyle, 2016). These observations support the hypothesis that CP group II RNA catalysis generates RNA circles using a two-step reaction mechanism analogous to that of group II ribozymes, with the circular product representing a structurally distinct version of the conjoined linear exons formed by canonical group II introns. Among the CP group II representatives we have identified, these circular products are predicted to range in length from 15 to 86 nt.

In group II introns, splice site selection is guided by exon-binding sites (EBS) residing within D1, which base-pair with intron-binding sites (IBS) occurring just beyond the group II ribozyme boundaries (Michel and Ferat, 1995). A subdomain within D1 of CP group II RNAs contains a conserved terminal loop sequence, a portion of which is complementary to a linker segment abutting the 5′ SS (Figure 6B). By analogy with group II ribozymes, this subdomain is likely to serve as EBS1. As is typical of many individual substructures of group II introns, the secondary structures of subdomains bearing EBS are observed to vary considerably among representatives of this class. This also appears to be the case with the subdomain in CP group II RNA that contains the putative EBS1. Whereas a variety of secondary structures occupy this position within D1, they share a similarly conserved terminal loop exhibiting complementarity to the 3′ end of the excised intervening sequence (Figure S3). Thus, the EBS-IBS base-pairing interaction appears to be conserved in CP group II RNAs, suggesting that it represents another architectural component that is shared with canonical group II ribozymes.

Some group II introns form another base-pairing interaction, designated δ-δ′, between the nucleotide immediately preceding EBS1 and the first nucleotide of the 3′ exon (Michel et al., 1989). There is the potential for base-pairing between the analogous nucleotides in *C. testosteroni* CP group II RNA (Figure 6B). In fact, as the nucleotide identities at these positions are widely conserved among CP group II ribozymes (Figures 1 and S3), a δ-δ′-type interaction might represent another feature common to group II and CP group II RNAs.

Having determined the splice sites used by CP group II RNA to remove and subsequently circularize the intervening sequence between D6 and D1, we next sought to define the junction created between D6 and D1 in the larger, putatively branched RNA product (BP) generated by CP group II ribozymes (Figure 4). RNAs corresponding to this proposed branched reaction product were purified using denaturing PAGE and subjected to RT-PCR using primers targeting sequences on opposite sides of the branchpoint adenosine (Figure 6C, top). The presence of a 2′-5′ linkage would be expected to impede the progress of reverse transcriptase, although certain types of reverse transcriptases are capable of reading through such branched structures (Lorsch et al., 1995). For a lariat-containing template with a branchpoint adenosine, it has been noted that reverse transcriptase misincorporates an adenosine residue rather than a complementary thymidine as it navigates through the branchpoint (Vogel et al., 1997). Following RT-PCR with gel-purified RNA corresponding to the BP band derived from the *Cte* 1 precursor (Figure 4), we detected DNA amplification products with sizes corresponding to reacted RNAs in which the intervening sequence had been excised. This population of DNAs was then gel-purified, subcloned, and sequenced.

Two sequences were detected in the pool of PCR products (Table S1). One sequence reveals a junction occurring between the nucleotide immediately 3′ to the 5′ SS and a thymidine residue in place of the branchpoint adenosine (Figure 6D). This is consistent with one of the products of CP group II ribozyme catalysis containing a 2′-5′ phosphodiester bond between the branchpoint adenosine and the conserved guanosine at the 5′ SS. Thus, the CP group II ribozyme appears to employ the branch-site adenosine as the nucleophile in the first step of the splicing reaction, as does the canonical group II intron. Interestingly, the other sequence that was detected among the RT-PCR products represents a direct fusion between the nucleotides defining the 5′ and 3′ splice sites (Figure 6D). In group II ribozymes, this type of junction, in which the 5′ and 3′ ends of the intron itself are linearly conjoined, results from the circularization pathway, and requires an initial *trans-*splicing step involving release of the 3′ exon (Murray et al., 2001; Monat et al., 2015). The utilization of an analogous secondary splicing pathway by CP group II ribozymes might therefore generate a linear product in which the region between the 5′ and 3′ splice sites has been removed (Figure 6C, bottom). Such a reaction product is predicted to have an electrophoretic mobility similar to that of the branched product, and thus it is possible that these two types of RNA molecules could have been co-purified from the isolated product band. Alternatively, the CP group II sequences conjoined at the splice sites might be derived from template switching during reverse transcription of the branched product. For 2′-5′ branched RNAs *in vitro*, template switching by reverse transcriptase from the 2′ arm to the 3′ arm has been reported (Pratico and Silverman, 2007), and it is possible that a similar event could be responsible for the second population of junction sequences.

### Mutations of conserved residues affect canonical and permuted group II ribozymes analogously

**Figure 7:**
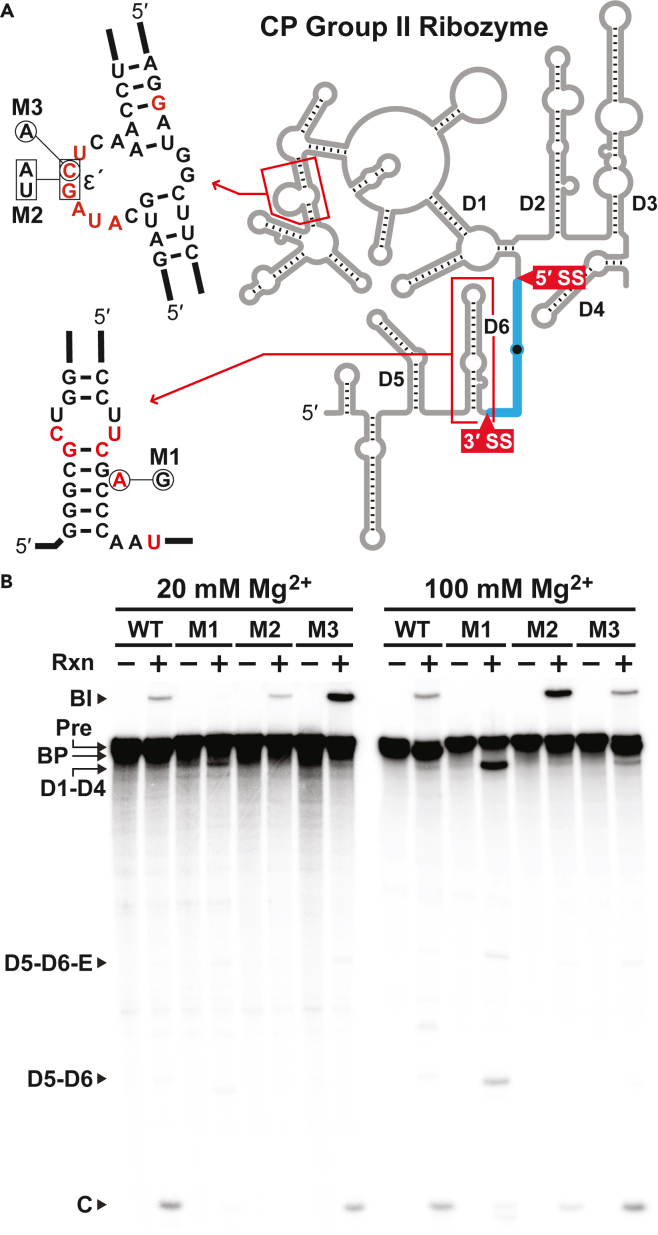
Effects of mutations reinforce parallels between CP group II and group II ribozymes (A) Secondary structures corresponding to the regions in *C. testosteroni* CP group II RNA that contain the proposed ε' site and the branchpoint adenosine, where mutations (M1, M2 and M3) were introduced at specific positions as indicated. Other annotations are as described for Figure 6B. (B) Ribozyme activities of internally ^32^P-labeled wild-type (WT) and mutant CP group II RNAs were assessed by subjecting samples that were unreacted and reacted for 2 h at 23°C to denaturing 6% PAGE. All precursor RNAs were 902 nt in length. Prominent bands in the M1 plus 100 mM Mg^2+^ lane are predicted to represent RNA products spanning D1-D4 (3′ fragment of Pre hydrolysis at the 5′ SS), D5-D6-E (5′ fragment of Pre hydrolysis at the 5′ SS that retains the exons), and D5-D6 (remainder of D5-D6-E after the second step of splicing removes the exons via circularization to produce band C). Other annotations are as described for Figure 4.

It was expected that CP group II RNAs containing mutations of conserved elements would be impaired catalytically, and that a given mutation would have effects on CP group II ribozyme catalysis similar to those exhibited by canonical group II ribozymes bearing analogous nucleotide changes. We tested a mutant version of *Cte* 1 RNA in which the highly conserved branch-site adenosine was replaced with a guanosine residue (Figure 7A). In the presence of 20 mM MgCl_2_, this construct (M1) generated diminished levels of the branched intermediate and circular products (Figure 7B), consistent with the demonstrated role of this nucleotide in the first transesterification step (see discussion above).

At higher Mg^2+^ concentrations, reactions with M1 RNA yield two prominent unique bands that probably correspond to the linear D1-D4 and D5-D6 fragments resulting from hydrolysis of the 5′ splice site during step 1 and subsequent release of the circular “exons” during step 2. Another unique band that is generated by M1 RNA at high Mg^2+^ concentration has a gel mobility consistent with a D5-D6-E product (E represents “exons”), which is a product of hydrolysis at step 1 that has not yet undergone step 2 of the splicing reaction. If our predictions about these products are correct, then the effects of mutating the branchpoint adenosine in CP group II RNA are consistent with the effects of mutating the analogous nucleotide in canonical group II introns (Liu et al., 1997).

The ε-ε′ interaction is mediated by long-range Watson-Crick base-pairing between a conserved GC dinucleotide near the 5′ SS and its complement, located within a conserved substructure in D1 (Jacquier and Michel, 1990) (Figures 1 and7A). In group II introns, this tertiary interaction participates in selection of the 5′ SS, but also makes important contributions to catalysis, as it is involved in a network of critical contacts with other key structural domains, including the internal loop at the base of D3 (Fedorova and Pyle, 2008; Boudvillain and Pyle, 1998). To assess the effects on CP group II catalysis of disrupting the putative ε-ε′ interaction, we constructed double (M2) and single (M3) mutants at the ε' site in D1 of *Cte* 1 (Figure 7A).

At lower Mg^2+^ concentrations, no circular spliced products were detected using the M2 construct, despite the accumulation of branched intermediates (Figure 7B). In the presence of 100 mM Mg^2+^, however, the circular product was detected and the branched intermediate accumulated to a greater extent, probably because the higher concentration of divalent metal ions partially compensates for structural defects in the M2 RNA. Not surprisingly, the effects on CP group II RNA catalysis of mutating only a single conserved nucleotide (M3) were less debilitating. The RNA circle was produced under the lower Mg^2+^ concentration, while at higher ionic strength, a smaller proportion of the population appeared to be trapped as branched intermediates, with the apparent accumulation of both branched and circular splice products (Figure 7B). Disruption of ε-ε′ base-pairing has similarly adverse effects on canonical group II intron self-splicing, resulting in greatly reduced accumulation of the intron lariat and spliced exon products (Jacquier and Michel, 1990). These results provide further evidence of the high degree of relatedness between the group II and CP group II ribozyme architectures.

### Proposed pseudoknots unique to CP group II RNAs

Although many of the tertiary structure interactions that organize the global fold of group II introns appear to be shared by the permuted version of the RNA (Figures 1 and 2), CP group II ribozymes also possess a number of predicted long-range base-pairing interactions that are not known to have counterparts in canonical group II introns (Figure 1). It is hypothesized that these pseudoknots assist in maintaining the relative spatial positioning of the structural domains analogously to that of the canonical group II intron, despite the permuted order in which these domains occur. The proposed pseudoknot unique to CP group II RNAs that is most widely distributed among known representatives, and whose existence is convincingly supported by covariation, is formed between the terminal loops of P2 and D2 (Figure 1). The other two proposed pseudoknots observed uniquely in CP group II ribozymes appear to be somewhat more restricted phylogenetically.

To assess the role of the highly conserved P2-D2 pseudoknot in CP group II RNAs, we prepared two constructs derived from a representative encoded on the *Thermus thermophilus* chromosome (Figure S4A). In an attempt to isolate the contribution to catalysis of the P2-D2 loop-loop interaction, these constructs were distinguished solely by whether or not they contained the P2 stem element. *Tth*1 RNA, in which the more narrowly distributed pseudoknot between P1 and the 3′ extremity of CP group II RNA is omitted, nonetheless undergoes processing in the presence of 20 mM Mg^2+^, generating the circular reaction products characteristic of CP group II catalysis (Figure S4B). In the presence of 100 mM Mg^2+^, *Tth* 1 RNA processes to a fuller extent, and the accumulation of the branched intermediate is more pronounced. In contrast, a second construct, *Tth* 2, which lacks P2 and therefore precludes formation of the pseudoknot with the D2 loop, appears to be catalytically inactive under the lower Mg^2+^ conditions (Figure S4B).

In 100 mM Mg^2+^, however, *Tth* 2 RNA generates the branched intermediate and the circular product, indicating that the higher Mg^2+^ concentration compensates for the deletion of P2. Although the deleterious effects of removing the P2 element are likely derived from disruption of the conserved pseudoknot, other contributions to catalysis of the P2 stem itself cannot be ruled out. These results imply a role in structure stabilization for the P2-D2 loop-loop interaction. By extension, the additional proposed pseudoknots in Figure 1 might also be involved in structure stabilization, particularly in extreme thermophiles like *T. thermophilus*.

### Broad conservation of the reaction catalyzed by CP group II ribozymes

We tested the activities of CP group II RNA representatives from three additional species of bacteria. Alongside the previously examined constructs from *C. testosteroni*, we assayed full-length constructs derived from *Acinetobacter radioresistens*, *Polaromonas naphthalenivorans*, and *Thiomonas intermedia* in 20 mM and 100 mM Mg^2+^. Reactions with CP group II RNAs from each of these organisms generated apparent branched intermediates as well as the expected circularized intervening sequences (Figure S5). The presence of the circles implies the existence of the branched products, although these longer products are expected to migrate closely to the precursor RNAs, and are therefore not resolved by PAGE.

For the reaction using *P. naphthalenivorans* RNA, production of the branched intermediate required a higher Mg^2+^ concentration and was more readily detected with a shorter construct (*Pna* 2) that lacks presumably extraneous sequences on the 5′ and 3′ ends. With the exception of the *A. radioresistens* RNA under lower salt, circles were generated by all constructs at both Mg^2+^ concentrations. Similarly to *Cte* 1 and *Cte* 2 RNAs (Figures 5A, 5B, and S5), two constructs from *P. naphthalenivorans* differing in the presence of extensions at the 5′ and 3′ ends produce identical circular products, providing support for a broadly conserved mechanism of CP group II RNA catalysis. Moreover, the predicted lengths of the circular products corresponding to *A. radioresistens*, *C. testosteroni*, *P. naphthalenivorans* and *T. intermedia* are 50, 86, 32 and 35 nt, respectively — sizes that are internally consistent among the small product bands observed (Figure S5).

Thus, five (including *T. thermophilus*; Figure S4B) arbitrarily selected representatives of the CP group II class appear to generate identically processed RNA products, and this supports the conclusion that the biochemical roles performed by these ribozymes are being preserved through evolution. If these molecules represent incidentally recombined, cryptic variants of group II introns with no conserved biological role, it seems unlikely that the highly conserved sequences and structural domains required for autocatalysis of these large RNAs would be maintained absent a valuable purpose for the cell or possibly for a selfish genetic element.

### Expression of CP group II RNA in bacteria

To assess whether CP group II RNAs are expressed in bacteria, we subjected total RNA purified from *P. naphthalenivorans* to RT-PCR with primers selectively targeting the ribozyme. CP group II RNA is encoded near the origin of replication on plasmid pPNAP05, a 59 kb plasmid that is one of eight extrachromosomal elements in this organism. Reverse transcriptase was used to synthesize cDNA with the appropriate reverse primers, and these cDNAs were subsequently amplified using PCR. First, three primer pairs were employed that targeted sequences on one side or the other of the branchpoint adenosine in D6 (Figure S6A).

Because these primer pairs are contained wholly within either the D5-D6 or D1-D4 segments, they are expected to yield amplification products of the same size regardless of whether they utilized reacted or unreacted CP group II RNA as a template. Indeed, the RT-PCR products correspond in size to the respective DNAs amplified from genomic DNA (Figure S6A). Importantly, amplification from total RNA depends on the presence of reverse transcriptase, indicating that the amplicons are not derived from contaminating genomic DNA. Thus, transcripts containing CP group II RNA are expressed in *P. naphthalenivorans* under the growth conditions used in this study.

In an attempt to detect processed forms of CP group II RNA in *P. naphthalenivorans*, we employed three pairs of primers that targeted sequences on opposite sides of the branch-site adenosine (Figure S6B). In this way, templates in which the intervening sequence had been excised would result in PCR products slightly smaller than those amplified from genomic DNA or from precursor RNA transcripts. However, we detected no such additional band with faster gel mobility (Figure S6B). Thus, although we can conclude that CP group II RNAs are expressed in *P. naphthalenivorans*, it is not yet apparent that these RNAs undergo self-processing under the growth conditions employed.

### Identification of a conserved ORF frequently associated with CP group II RNA

CP group II RNAs are encoded at two loci in *T. thermophilus* HB8, one occurring in the chromosome and the other in plasmid pTT8. In both instances there are ORFs immediately upstream, which are oriented divergently with respect to the CP group II RNAs. The corresponding proteins, which were initially annotated as TTHC007 (the locus tag of the plasmid copy), are 88% identical and each contain ∼260 amino acids. A search for related proteins revealed homologs in multiple phyla that are encoded in proximity to CP group II RNAs. The corresponding ORFs are often immediately adjacent to CP group II genes, and while the protein and the RNA are sometimes encoded on the same strand, they are also frequently oriented divergently or convergently with respect to one another (Data S1). Immediate proximity is not always observed, however, as the TTHC007 ORF sometimes resides several kilobases away from the CP group II RNA gene. Due to the genetic association of this ORF with CP group II RNA in multiple bacterial phyla, we recommend designation of the corresponding protein as AcpG (Associated with CP Group II RNA).

Despite the recurrent proximity of these genes to each other, there are nonetheless many CP group II representatives with no detected AcpG homologs encoded nearby, and there are also cases where the *acpG* gene appears to be entirely absent from genomes that contain CP group II RNA. Thus, the apparent genetic association might not be indicative of a physical interaction between CP group II RNA and AcpG. Beyond the genetic association, there are currently no clues regarding the biochemical function of AcpG, as no homology with any other known protein class has been detected.

## Discussion

Among naturally occurring ribozymes, circular permutation is observed commonly among certain classes of small self-cleaving RNAs, including the hammerhead and twister classes (Roth et al., 2014). But the primary demand placed upon such ribozymes is cleavage at a single site within an RNA strand. Where the 5′ and 3′ ends occur within the structure is of little consequence, providing the catalytic core remains intact, and so different permuted forms of self-cleaving ribozymes are likely to be functionally indistinguishable. For large self-splicing ribozymes, however, domain order is paramount. The group II intron catalyzes two sequential RNA cleavage reactions in the course of autoexcision from a transcript. For this excision to proceed neatly, the two splice sites must reside at the 5′ and 3′ extremities of the ribozyme structure. Any change in the arrangement of these splice sites will lead to substantive changes in the reaction products.

The CP group II family provides a striking natural example of a self-splicing ribozyme whose domains and splice sites have been rearranged in such a manner. In this permuted version of the group II ribozyme, the splice sites occur in reverse order within the interior of the primary structure, typically separated from one another by less than 100 nt. Relying on reaction mechanisms analogous to those of canonical group II introns, the CP group II ribozyme catalyzes a “back-splicing” reaction (Lasda and Parker, 2014) in which the intervening sequence between the splice sites is excised as a small circular product. And instead of a conventional lariat intron RNA (Peebles et al., 1986), CP group II ribozymes generate a branched, T-shaped product in which the 5′ domains of the ribozyme are tethered to the 3′ domains via a 2′-5′ phosphodiester link.

Especially given that the reaction catalyzed by CP group II RNA could be viewed as a seemingly futile one, it could be hypothesized that CP group II RNAs are merely stochastically misordered remnants of canonical group II introns, having retained their biochemical activity but serving no biological role. Indeed, fragments of group II ribozymes encoded at separate loci in plant mitochondria can assemble in *trans* (Bonen, 2008), and circularly permuted derivatives of group II introns that were engineered in the laboratory also catalyze back-splicing, yielding large-branched and small circular RNA products similar to those observed with naturally occurring CP group II ribozymes (Jarrell, 1993). Thus, permutation in itself certainly does not create an impediment to assembly of a working active site.

For a number of reasons, though, it seems unlikely that representatives of the CP group II class are cryptic RNAs that all arose incidentally. In this study we tested CP group II RNAs from five bacterial species, all of which were catalytically active *in vitro* and generated the same set of reaction products (Figures S4 and S5). The possibility seems remote that a large complex ribozyme with no biological role would nonetheless exhibit conservation of all the key structural features required for catalysis. In addition, the majority of bacterial group II introns contain ORFs encoding IEPs (Simon et al., 2008), whose various domains are critical for retromobility. Yet ORFs corresponding to these IEPs are never observed within CP group II RNAs or in proximity to them. If, in fact, CP group II RNAs are simply haphazardly recombined group II introns, it would be surprising for the conserved ORF to be consistently jettisoned while all other structural domains of the ribozyme are retained.

Furthermore, although the respective structural domains that comprise CP group II and canonical group II ribozymes are strikingly similar, the former class contains features that are not shared by canonical group II introns. Three pseudoknots are proposed for CP group II RNAs that are not observed among canonical group II introns. Moreover, two of these predicted pseudoknots involve stem-loops at the 5′ end that also are unique to CP group II RNAs. One general consequence of permutation might be destabilization of the global tertiary fold. Indeed, another class of self-splicing intron, the group I RNA, exhibits deficiencies in its folding pathway when it is circularly permuted in the laboratory (Lease et al., 2007). Thus, the roles of the structural features unique to CP group II RNA might be to reinforce interdomain contacts that might have been destabilized as a result of permutation, and this is consistent with our observation that higher Mg^2+^ concentrations are required to support the catalytic activity of a mutant CP group II RNA in which some of these unique elements have been deleted (Figure S4). Taken together, these observations suggest that the CP group II family represents a distinct ribozyme class with a biological function differing from that of canonical group II ribozymes.

Group II introns in bacteria behave primarily as selfish retroelements (Zimmerly and Semper, 2015), and it is therefore important to consider the possibility that the permuted variants have a similar function. The IEP ORFs commonly contained within group II ribozymes encode reverse transcriptase and endonuclease domains that are essential for the life cycle of the retro-element. In contrast, the circularly permuted variant appears not to accommodate any ORF within its structure. Therefore, if any aspect of the biological role of CP group II RNA relied upon a retrotransposition event, reverse transcriptase activity would have to be provided by an enzyme encoded at a different locus. Notably, the use of distally encoded reverse transcriptase enzymes by group II ribozymes lacking an IEP has been reported (Simon et al., 2008; Meng et al., 2005), and so this remains a possibility for CP group II RNAs.

The group II intron also relies on a maturase domain within the IEP to help guide the branch-point of this elaborately folded RNA (Haack et al., 2019; Zimmerly et al., 2001). The CP group II RNA, although its domains have been shuffled, remains nonetheless a similarly large and complex structured RNA. In many respects, then, for this RNA to be relieved of the requirement for protein-mediated assistance *in vivo* would be surprising. The proposed long-range pseudoknots occurring uniquely in CP group II ribozymes help to promote ribozyme activity at lower Mg^2+^ concentrations, and it is possible that the structural reinforcement they provide is sufficient for activity *in vivo*. But it could also be the case that additional factors, encoded at other loci, are necessary for the biochemical function of CP group II RNA. The AcpG proteins that are often encoded near CP group II RNA genes might be involved in such a role, although it appears that at least some genomes with CP group II RNA lack *acpG* homologs. The absence of a requirement for an associated protein *in vivo* would not necessarily be without precedent, however, as it has been speculated that a group II ribozyme from *Clostridium tetani* might function independently of such factors *in vivo* (McNeil and Zimmerly, 2014).

If CP group II RNAs were to behave as a type of selfish element, it is not at all clear how the reactions catalyzed by this RNA *in vitro* would permit propagation. Canonical self-splicing group II introns are excised in their entirety, and thus carry all the information necessary to perform the splicing reaction in reverse, inserting into DNA with the aid of the IEP to complete the life cycle. CP group II RNAs, in contrast, would largely remain embedded in their corresponding transcripts after back-splicing, albeit with a branched structure containing a 2′-5′ link and loss of the sequence corresponding to the circular product. In principle, reversing the back-splicing reaction would result in cleavage of a DNA strand *in vivo*, but such a reaction would not restore the original domain order of CP group II RNA, nor would it repair the DNA lesion. Furthermore, there would be a loss of information corresponding to the circularized intervening sequence. Thus, a pathway that would support mobility of this RNA as a self-contained unit is difficult to envision. Rather, the ability of the CP group II ribozyme to disrupt the integrity of an RNA or DNA sequence could be exploited by mobile elements with which it might be associated, or it could be used as a form of defense against invading nucleic acids.

If CP group II RNA is not itself a mobile element and does not work in concert with a different one, then it must provide some benefit to the organisms in which it resides for its complex structure to remain so highly conserved. Self-splicing introns often restore interrupted ORFs, allowing translation of the corresponding protein, and such types of joining reactions can even be mediated by a permuted version of a self-splicing ribozyme. A permuted group I precursor RNA occurring in the mitochondria of mushroom corals employs back-splicing so that exons that are misordered on the linear precursor are appropriately spliced in the resulting circular product (Chi et al., 2019). CP group II RNAs appear to stand alone, however, never having been observed to interrupt or precisely abut an ORF. Moreover, the splice sites are contained within the interior of the ribozyme. Thus, any effects they might exert on gene expression are not likely to result from splicing per se.

It is conceivable, though, that CP group II reaction products might affect transcript stability in *cis*. The introduction of a branched structure containing a 2′-5′ phosphodiester bond might render the transcript resistant to exonucleases, for example, thereby extending its half-life and influencing the expression levels of any genes co-expressed with the CP group II ribozyme. It is also possible that CP group II RNAs could exert effects in *trans*. Group II ribozymes are known to destabilize the spliced transcripts from which they were derived. The excised intron lariat recognizes the spliced mRNA via EBS-IBS base-pairing, and catalyzes reverse splicing and spliced exons reopening reactions (Jarrell et al., 1988) to reduce the level of spliced message, consequently inhibiting gene expression (Qu et al., 2018). The branched product generated by the CP group II ribozyme would presumably have the analogous capability to target RNAs or DNAs for destruction in *trans*, simply by catalyzing its reverse reactions.

*Trans*-acting effects of the small circular product on gene expression or other processes also cannot yet be ruled out. However, the sequences of CP group II RNAs corresponding to the excised circles are less conserved, and thus, despite its presumed increased stability, the circular product is unlikely to be the primary agent of CP group II RNA function. Nonetheless, even if RNA circles are by-products of CP group II RNA catalysis, this aspect of the reaction could be exploited as a tool. There is an increasing appreciation for the diverse biological roles of circular RNAs (Xiao et al., 2020; Ebermann et al., 2020), and CP group II ribozymes engineered to contain customized ‘exon’ sequences could provide a convenient method for the production of the corresponding circles *in vitro* and even *in vivo*.

CP group II RNAs are often encoded on plasmids. There are also certain subclasses of canonical group II introns that have a propensity toward plasmid residence. This is due in part to target sites that are more relaxed than those on the bacterial chromosome, promoting reverse splicing into transiently single-stranded regions of DNA (Ichiyanagi et al., 2003). But because there is no obvious mechanism for retrotransposition of CP group II RNAs, the reasons for their occurrences on plasmids are likely to be different. A CP group II RNA is encoded on plasmid pTT8 in *T. thermophilus* HB8. Interestingly, nearly the entire sequence encoding the ribozyme can be deleted without any significant impact on autonomous replication of the plasmid (Aoki and Itoh, 2007). Thus, a role for CP group II RNA in the replication of this plasmid perhaps can be eliminated. It should be noted, however, that another CP group II RNA is encoded on the *T. thermophilus* chromosome, and so the presence of this copy might serve to complement the pTT8 deletant.

Existing enzymes often provide the evolutionary source material for the emergence of new ones. Even among the few known classes of large ribozymes, such an evolution of new activity from old is known to have occurred. The lariat-capping (LC) ribozyme appears to be a descendant of the group I self-splicing intron, although its three-dimensional structure has diverged significantly and its reaction mechanism and biological function are distinct (Meyer et al., 2014; Nielsen et al., 2005). Unlike its group I intron ancestor, which catalyzes a two-step splicing reaction, the LC ribozyme mediates a one-step branching reaction that results in a tiny loop at the 5′ end of an mRNA, which probably confers protection from 5′ exonucleases. For CP group II RNA, however, the differences between its overall structure and that of its group II parent are expected to be minor, as the reaction mechanism appears to be shared by the canonical and permuted versions of these RNAs. Thus, the CP group II class of ribozymes illustrates how a simple rearrangement in primary structure can lead to distinct reaction products and potentially to an entirely different biological function.

### Limitations of the study

CP group II ribozymes from five species of bacteria generate the same set of reaction products when assayed *in vitro*, and thus it seems reasonable to conclude that the same products are formed by CP group II catalysis in cells. However, although expression of CP group II RNA is detected in a native host organism, no direct evidence is provided that this self-processing reaction occurs in cells. Also, no role is known for the protein encoded by *acpG*, which is the gene that tends to associate with CP group II sequences. Lastly, the biological role of CP group II ribozyme catalysis has not yet been determined.

## STAR★Methods

### Key resources table

**Table:** 

REAGENT or RESOURCE	SOURCE	IDENTIFIER
**Bacterial and virus strains**		

*Polaromonas naphthalenivorans* CJ2	ATCC	ATCC BAA-779

**Biological samples**		

*Thermus thermophilus* HB8 cell pellet	Thomas Steitz	N/A
*Comamonas testosteroni* KF-1 genomic DNA	David Schleheck	N/A
*Acinetobacter radioresistens* SH164 genomic DNA	Harald Seifert	N/A
*Thiomonas intermedia* K12 genomic DNA	Sabine Heinhorst	N/A

**Chemicals, peptides, and recombinant proteins**		

[α-^32^P] ATP	PerkinElmer	BLU503H250UC
[α-^32^P] GTP	PerkinElmer	BLU506H250UC
[γ-^32^P] ATP	PerkinElmer	BLU502Z250UC

**Oligonucleotides**		

See Table S2 for oligonucleotides	MilliporeSigma	N/A

**Software and algorithms**		

ImageQuant	GE Healthcare Life Sciences	N/A
JackHMMER	Johnson et al., 2010	http://hmmer.org

### Resource availability

#### Lead contact

Further information and requests for resources and reagents should be directed to and will be fulfilled by the lead contact, Ronald Breaker (ronald.breaker@yale.edu).

#### Materials availability

This study did not generate new unique reagents.

### Experimental model and subject details

#### Microbe strain

*P. naphthalenivorans* CJ2 (ATCC BAA-779) was cultured at 20°C in medium based on ATCC Medium 2258 R2A.

#### Sources of genomic DNA and total RNA

*Polaromonas naphthalenivorans* CJ2, *Thermus thermophilus* HB8, *Comamonas testosteroni* KF-1, *Acinetobacter radioresistens* SH164, and *Thiomonas intermedia* K12 were used as sources of genomic DNA. *Polaromonas naphthalenivorans* CJ2 was used as a source of total RNA.

### Method details

#### Assays *in vitro* of CP group II ribozymes

Synthetic oligonucleotides were purchased from MilliporeSigma. Template DNAs were amplified by PCR using genomic DNA from *C. testosteroni* KF-1, *T. thermophilus* HB8, *A. radioresistens* SH164, *T. intermedia* K12, and *P. naphthalenivorans* CJ2 (ATCC BAA-779) using the appropriate primers (Table S2). Following purification with the QIAquick PCR Purification Kit (QIAGEN), template DNAs were used in transcriptions *in vitro* as previously described (Roth et al., 2006), except that they included 10 μCi [α-^32^P] ATP or [α-^32^P] GTP. Precursor RNAs were then purified by denaturing (8M urea) 6% polyacrylamide gel electrophoresis (PAGE) and eluted from gel slices in 10 mM Tris-HCl (pH 7.5), 200 mM NaCl, 1 mM EDTA. The recovered material was concentrated by precipitation with ethanol. Gel-purified RNAs were heated to 80°C for 1 min in 40 mM HEPES (pH 7.5 at 23°C), then cooled to and incubated at 23°C for the designated times in the presence of 40 mM HEPES (pH 7.5 at 23°C), 20 mM or 100 mM MgCl_2_ (as indicated), and 0.5 M (NH_4_)_2_SO_4_. Precursor RNAs from *T. thermophilus* were subjected to an initial denaturation for 1 min at 90°C, cooled to 60°C (0.5°C/s), and incubated in the reaction buffers described above at 60°C. Reactions were terminated with equal volumes of urea-containing gel loading buffer (87 mM Tris base, 89 mM boric acid, 20% sucrose, 0.05% bromophenol blue, 0.05% xylene cyanol, 0.1% sodium dodecyl sulfate, 7.3 M urea, 100 mM EDTA). Reaction products were separated by denaturing PAGE, and a PhosphorImager and ImageQuant software (GE Healthcare Life Sciences) were used to visualize radiolabeled species. Molecular weight markers were prepared by dephosphorylating 1 μg RiboRuler Low Range RNA Ladder (Thermo Fisher Scientific) with rAPid alkaline phosphatase (Roche Life Sciences) according to the manufacturer's protocol. The resulting RNAs were radiolabeled at the 5′ ends using 30 μCi [γ-^32^P] ATP and T4 polynucleotide kinase (New England Biolabs) following the manufacturer's protocol. Unincorporated [γ-^32^P] ATP was subsequently removed using NucAway Spin Columns (Ambion).

#### Characterization of CP group II ribozyme reaction products

Internally ^32^P-labeled *Cte* 1 RNA was reacted for 2 h under the conditions described above. Gel-purified circular products were reverse transcribed using the appropriate primer and SuperScript II Reverse Transcriptase (Thermo Fisher Scientific) according to the manufacturer's protocol. The resulting cDNA was amplified by PCR, with a final 5 min incubation at 72°C to promote A-tailing. Subsequently, the PCR products were subcloned into the pCR 2.1-TOPO TA vector and the resulting plasmids were used in transformations of One Shot TOP10 competent cells according to the manufacturer's (Thermo Fisher Scientific) suggested protocols. Plasmid DNAs from the resulting colonies were prepared using the QIAprep Spin Miniprep Kit (QIAGEN) and sequenced.

The gel-purified branched products from a *Cte* 1 reaction as described above were reverse transcribed and subsequently PCR-amplified using the appropriate primers, but otherwise as described for the circular products. PCR products of the expected size were purified from an agarose gel using a QIAquick Gel Extraction Kit (QIAGEN) per the manufacturer's suggestions. The gel-purified DNA was then re-amplified by PCR, and the ensuing steps — subcloning, transformation, plasmid preparation and sequencing — were as described above.

#### Construction of mutant CP group II RNAs

Using the QuikChange II XL Site-Directed Mutagenesis Kit (Agilent) according to the manufacturer's instructions, a pCR 2.1-TOPO TA plasmid containing an insert corresponding to the full-length wild-type *C. testosteroni* CP group II ribozyme was used as a template with appropriate primers to generate plasmids bearing mutant versions of CP group II RNA. The resulting plasmids were then used in bacterial transformations, again following the manufacturer's instructions. Plasmid DNAs from transformants were prepared as described above and the presence of the desired point mutations in otherwise wild-type backgrounds was confirmed by sequencing. These plasmids were subsequently used as templates in PCRs with the appropriate primers to generate linear double-stranded templates for *in vitro* transcriptions.

#### RT-PCR amplification from total RNA

*P. naphthalenivorans* CJ2 (ATCC BAA-779) was purchased from ATCC. The growth medium was based on ATCC Medium 2258 R2A, and contained 0.5 g/L yeast extract, 0.5 g/L Proteose Peptone No. 3, 0.5 g/L casamino acids, 0.5 g/L glucose, 0.3 g/L sodium pyruvate, 0.3 g/L K_2_HPO_4_, and 0.05 g/L MgSO_4_× 7H_2_O, with the addition of monobasic anhydrous potassium phosphate (KH_2_PO_4_) to adjust the final pH to 7.2. Using pelleted cells from a 10 mL culture grown with shaking (175 rpm) at 20°C until turbid (72 h), total RNA was isolated using TRIzol (Ambion) according to the manufacturer's instructions. Pelleted cells from a 3 mL culture grown under the same conditions were used to prepare genomic DNA with a DNeasy Blood & Tissue Kit (QIAGEN) according to instructions provided by the manufacturer. RNA samples were treated with RQ1 RNase-free DNase (Promega), extracted successively with phenol/chloroform (1:1) and chloroform/isoamyl alcohol (24:1), and concentrated by precipitation with ethanol. Following a 1 min denaturation step at 80°C, 1 μg of DNase-treated RNA and the appropriate reverse primer were added to 20 μL reactions containing SuperScript III Reverse Transcriptase (Thermo Fisher Scientific) and to mock reactions not containing reverse transcriptase; incubations were for 2 h at 50°C under conditions recommended by the manufacturer. Subsequently, the products of these reverse transcription reactions and the genomic DNA preparation were used as templates in PCR amplifications with the appropriate primer pairs. The PCR products were then analyzed on 3% agarose gels stained with ethidium bromide.

### Quantification and statistical analysis

This manuscript does not include quantification or statistical analysis.

## Data Availability

All data reported in this paper will be shared by the lead contact upon request. This paper does not report original code. Any additional information required to reanalyze the data reported in this paper is available from the lead contact upon request.

## Supplemental information

Document S1. Figures S1–S6 and Tables S1 and S2

Data S1. Locations and sequence alignments of CP group II RNAs, related to Figure 1

Data S2. Sequence alignments of CP group II RNAs in machine-readable format, related to Figure 1

Data S3. Sequence alignments of AcpG proteins in machine-readable format, related to Data S1
