# Supplementary material for: Natural circularly permuted group II introns in bacteria produce RNA circles
Source: iScience. 2021 Nov 13;24(12):103431. doi: 10.1016/j.isci.2021.103431 (PMC8637638; doi:10.1016/j.isci.2021.103431)
Supplement: Document S1. Figures S1–S6 and Tables S1 and S2 [file mmc1.pdf]

iScience, Volume *24*

Supplemental information

Natural circularly permuted group II

introns in bacteria produce RNA circles

Adam Roth, Zasha Weinberg, Koen Vanderschuren, Mitchell H. Murdock, and Ronald R. Breaker

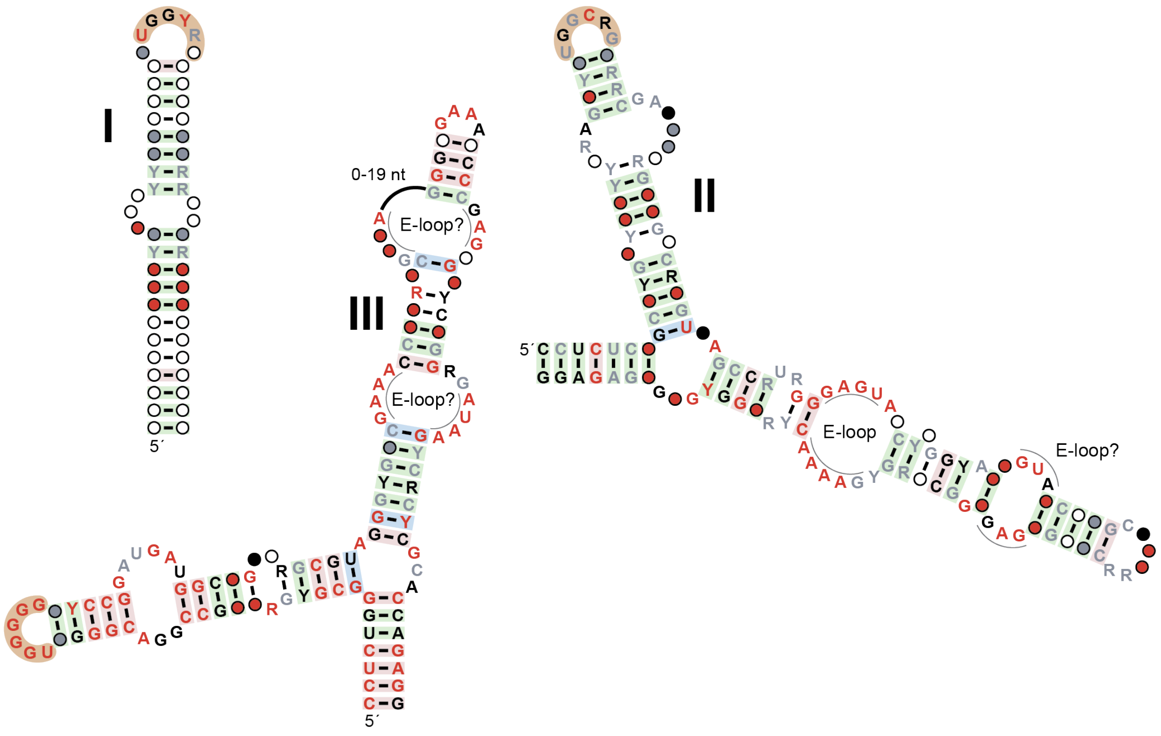

Figure S1. Consensus sequence and secondary structure models of three distinct types of D2 substructures of CP group II RNAs, related to Figure 1. Substructures I, II, and III occur in 58%, 25%, and 17% of CP group II RNA representatives, respectively. The conserved terminal loops characteristic of D2 are shaded in beige. “E-loop?” indicates substructures with only weak adherence to the E-loop consensus. Other notations are as described for Figure 1.

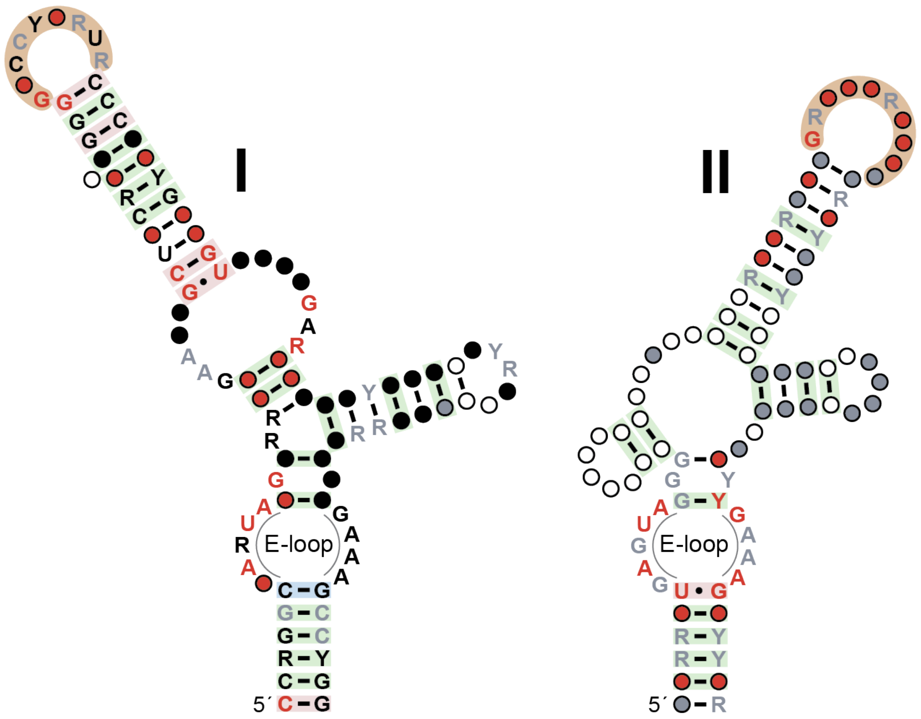

Figure S2. Consensus sequence and secondary structure models of two distinct types of D3 substructures of CP group II RNAs, related to Figure 1. Substructures I and II occur in 43% and 57% of CP group II RNA representatives, respectively. Substructure I exists only in species of the genus *Thermus*, whereas substructure II is usually observed in Proteobacteria. The most conserved terminal loops in this substructure are shaded in beige. Other notations are as described for Figure 1.

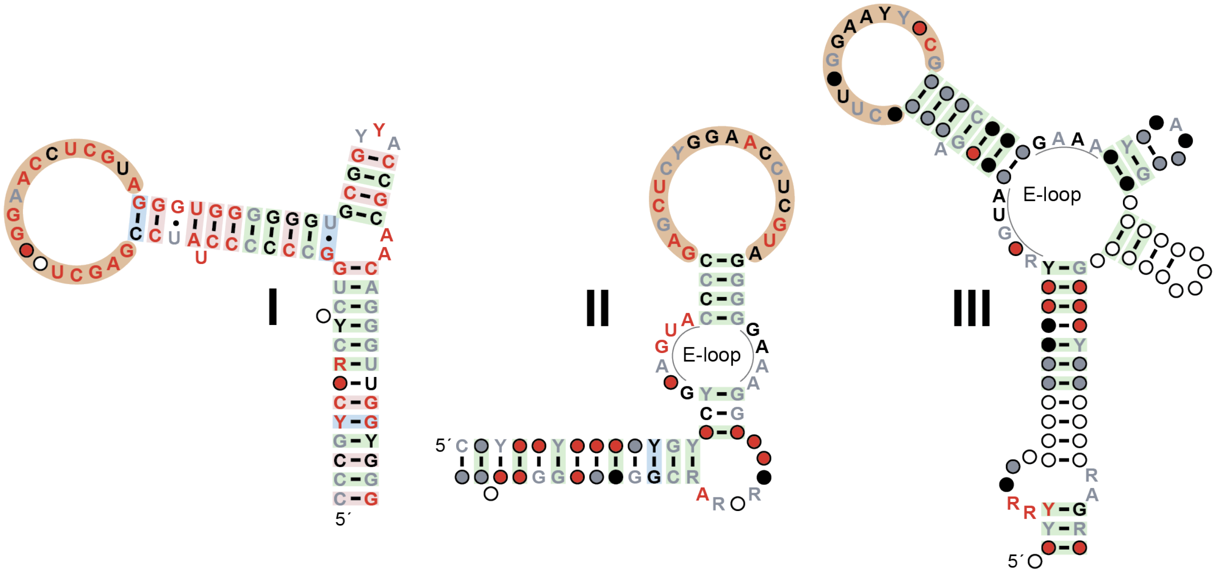

Figure S3. Consensus sequence and secondary structure models of three distinct types of D1 substructures of CP group II RNAs containing the EBS sequence, related to Figure 1. Substructures I, II, and III occur in 58%, 24%, and 17% of CP group II RNA representatives, respectively. Substructure I exists exclusively in species of the genus *Thermus*, whereas substructure II usually occurs in *Thermus* and environmental sequences. Substructure III is generally found in Proteobacteria. The conserved terminal loops of D1 carrying the EBS sequence are shaded in beige. Other notations are as described for Figure 1.

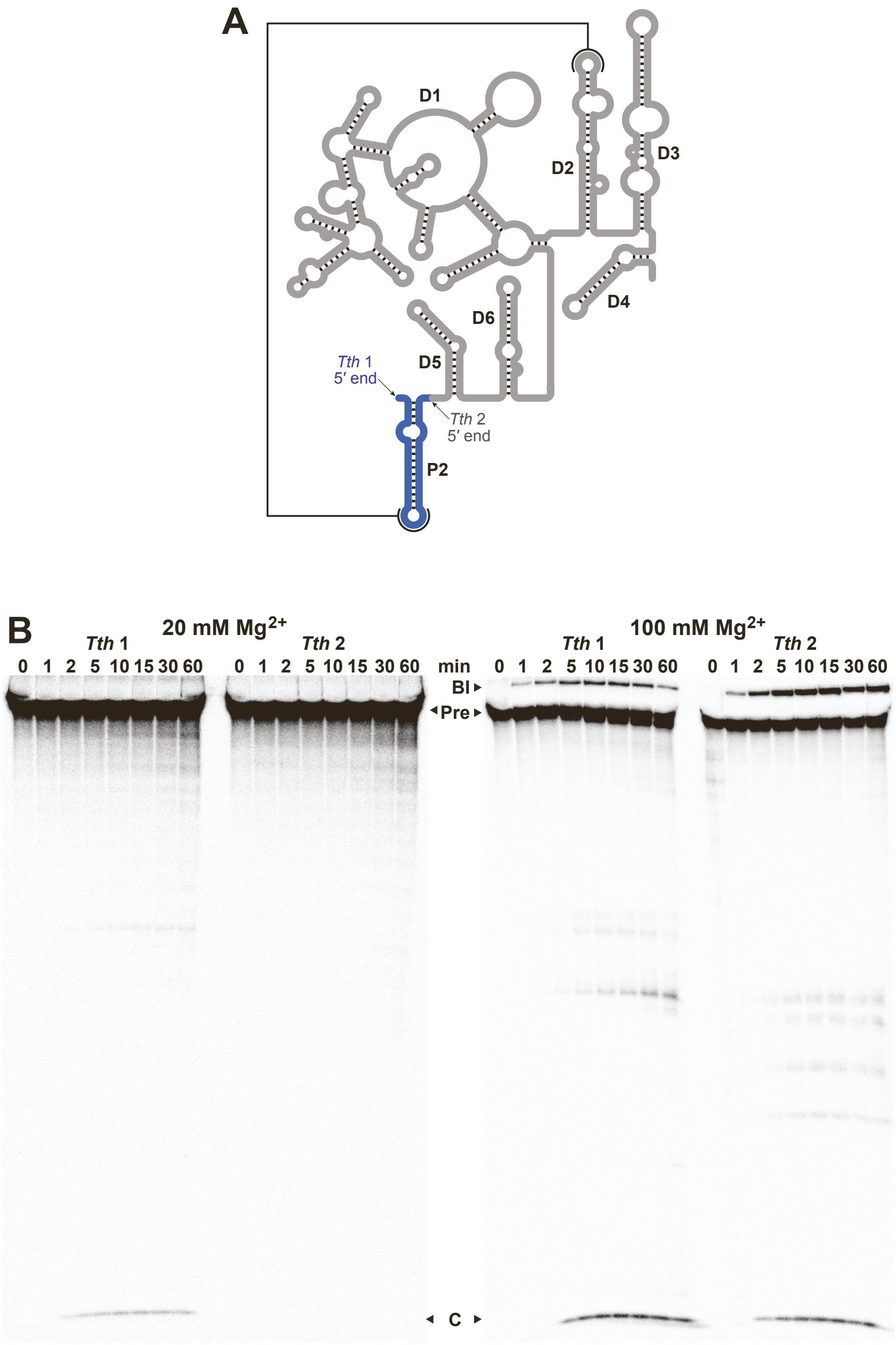

Figure S4. A proposed role for the P2-D2 loop-loop interaction in structure stabilization, related to Figure 1. **(A)** Schematic diagram highlighting the difference between the *Tth* 1 construct, which contains the P2 element, and the *Tth* 2 RNA, which lacks it. **(B)** Time courses of self-splicing reactions with internally ^{32}P-labeled *Tth* 1 and *Tth* 2 constructs (precursor RNAs are 979 and 936 nt, respectively) were analyzed by denaturing 7% PAGE. Other notations are as described for Figure 4.

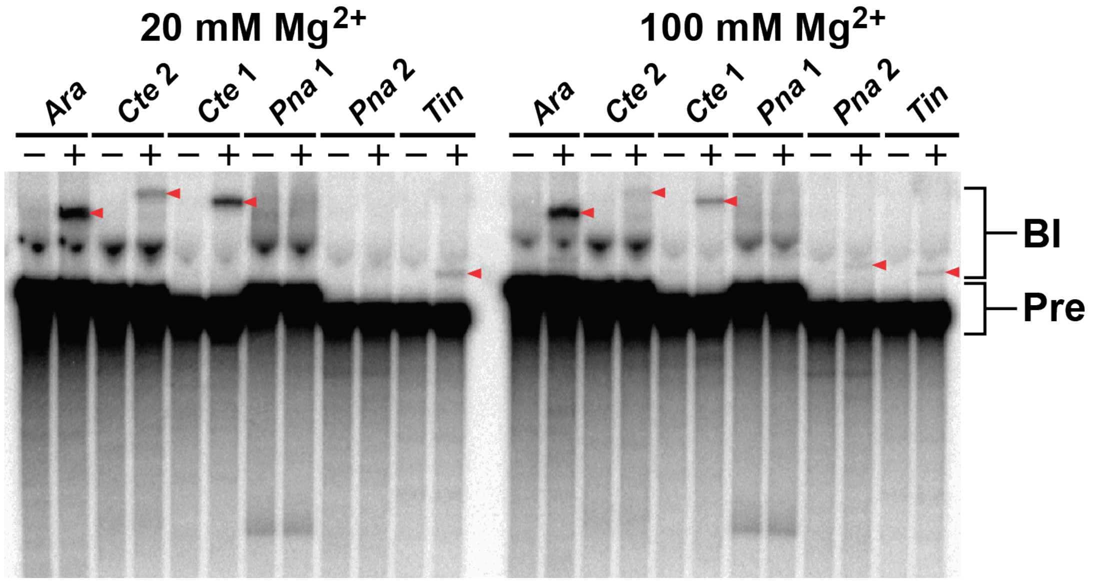

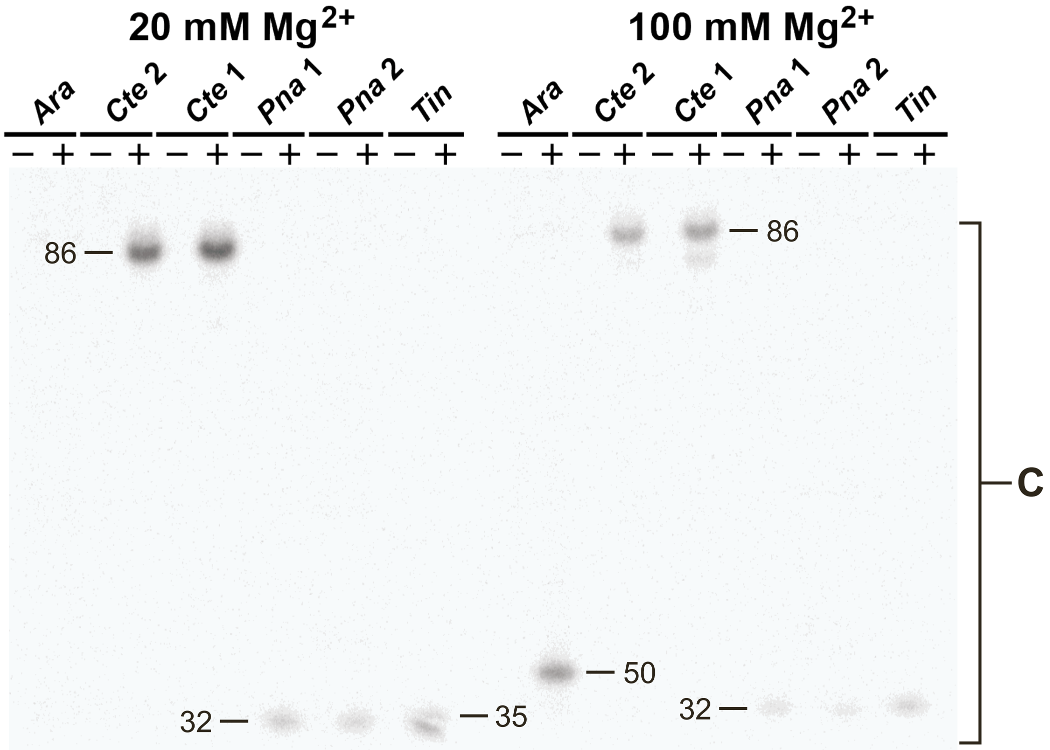

Figure S5. Characteristic reaction products are generated by CP group II ribozymes from multiple bacterial species, related to Figure 4. Unreacted and reacted (1 h at 23°C) internally ^{32}P-labeled CP group II precursor RNAs from *A. radioresistens* (*Ara*; 1019 nt), *C. testosteroni* (*Cte* 1 and *Cte* 2; 902 and 981 nt, respectively), *P. naphthalenivorans* (*Pna* 1 and *Pna* 2; 951 and 855 nt, respectively), and *T. intermedia* (*Tin*; 852 nt) were analyzed by denaturing 7% PAGE. Red arrowheads designate the positions of the branched intermediates (BI). Numbers denote the predicted lengths in nucleotides of the circularized exon RNA splice products. Other notations are as described for Figure 4.

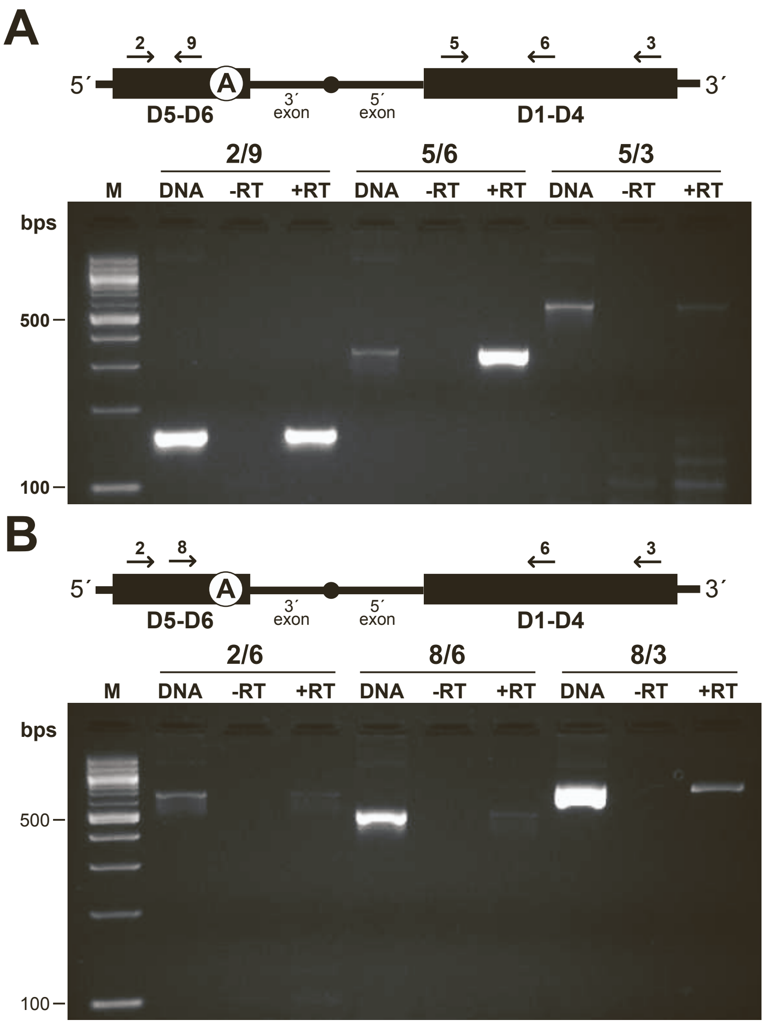

Figure S6. CP group II RNA is expressed in bacteria, related to Figure 6. **(A)** Top: Schematic depiction of unreacted CP group II RNA from *P. naphthalenivorans* and the locations targeted by DNA primers (numbered arrows) used for RT-PCR reactions. Bottom: RT-PCR reactions were conducted with total RNA isolated from *P. naphthalenivorans*. Note that the primer pairs used target sequences on one side or the other of the CP group II RNA branch-point adenosine. The resulting amplicons (+RT) were analyzed on a 3% agarose gel and compared to products amplified from *P. naphthalenivorans* genomic DNA (DNA) or from mock RT reactions in which reverse transcriptase was omitted (-RT). A 100 bp DNA ladder provided marker bands (M). **(B)** Top: The RNA construct described in (A), but depicting the binding locations of additional DNA primers. Bottom: RT-PCR reactions resulting from the use of primer pairs that target sequences on opposite sides of the branch-point adenosine. Other details are as described in (A).

Note: The RT-PCR product band in the +RT reaction for primer pair 5/3 is faint but evident. This product is absent in the -RT reaction lane. Similarly, the RT-PCR product bands for the +RT reactions for primer pairs 2/6 and 8/6 are faint, whereas they are absent in the -RT lanes.

Table S1. Sequences of RT-PCR products used to characterize CP group II reaction products, related to Figure 6. Vector sequences are in red. Highlighted regions correspond to partial [purple and blue] and full-length [yellow and green] exon circle sequences. A single point mutation is indicated in white. Sequences corresponding to domain six and domain one are in blue and green, respectively. The non-templated nucleotide is underlined.

**Segment/junction**	**Representative sequence**
Excised exon circle	CGAGCTCGGATCCACTAGTAACGGCCGCCAGTGTGCTGGAATT<br>CGCCCTTGCGGCGTACTGAGTGACAAACGAGGCAAAACCAAATT<br>GAGGTTCCATCTTGCGGCTCAACCCCGCAAGATCATCGCCAGAC<br>CGCTGGCGGCGTACTGAGTGACAAACGAGGCAAAACCAAATTG<br>AAGTTCCATCTTGCGGCTCAACCCCGCAAGATCATCGCCAGACC<br>GCTGGCGGCGTACTGAGTGACAAACGAGGCAAAACCAAATTGAA<br>GTTCCATCTTGCGGCTCAACCCCGCAAGATCATCGCAAGGGCGA<br>ATTCTGCAGATATCCATCACACTGGCGGCCGCTCGAGCATGCAT<br>CTAG
Branched product junction	GGATCCACTAGTAACGGCCGCCAGTGTGCTGGAATTCGCCCTTT<br>GCGAAGGGCGCTGGTGAGTGCAACTCTCACCTTCG<u>T</u>GGGCGAC<br>CGTGAAACGGCGCTGGGCAGGAAATGGCCCAGTGACCTGGTCA<br>ATGGAAGGGCGAATTCTGCAGATATCCATCACAC
Linear product junction	GCCCTTTGCGAAGGGCGCTGGTGAGTGCAACTCTCACCTTCGA<br>CCCAATGGGCGACCGTGAAACGGCGCTGGGCAGGAAATGGCCC<br>AGTGACCTGGTCAATGGAAGGG

Table S2. Oligonucleotide primer sequences, related to STAR Methods.

Name	Sequence (5´ to 3´)	Purpose
Cte2	TAATACGACTCACTATAGGCAAGCCGGGGCGCCACCCC	Forward primer for PCR amplification of *Cte* 1 template
Cte3	TGCCCGCCAAGGCCTGAGGTCGGC	Reverse primer for PCR amplification of *Cte* 1 template
Cte1	TAATACGACTCACTATAGGGCAGATCGCCAAACTGCAACCACACAC	Forward primer for PCR amplification of *Cte* 2 template
Cte4	TCCACGCCACTGCCGAATCCGCCAAG	Reverse primer for PCR amplification of *Cte* 2 template
Cte13	TTGCGAAGGGCGCTGGTGAGTGCAAC	Forward primer for RT-PCR using the branched/linear products as templates
Cte14	CCATTGACCAGGTCACTGGGCCATTTCC	Reverse primer for RT-PCR using the branched/linear products as templates
Cte15	GCGGCGTACTGAGTGACAAACGA	Forward primer for RT-PCR using the circular product as template
Cte16	GCGATGATCTTGCGGGGTTGAGC	Reverse primer for RT-PCR using the circular product as template
Cte17	CAACTCTCACCTTCGGCCCAATCCATCTTGC	Forward primer used to generate M1 DNA by site-directed mutagenesis
Cte18	GCAAGATGGATTGGGCCGAAGGTGAGAGTTG	Reverse primer used to generate M1 DNA by site-directed mutagenesis
Cte19	GGGGGTCGATGCATATATCAAACCTGTAACGG	Forward primer used to generate M2 DNA by site-directed mutagenesis
Cte20	CCGTTACAGGTTTGATATATGCATCGACCCCC	Reverse primer used to generate M2 DNA by site-directed mutagenesis
Cte21	GGGGGTCGATGCATAGATCAAACCTGTAACGG	Forward primer used to generate M3 DNA by site-directed mutagenesis
Cte22	CCGTTACAGGTTTGATCTATGCATCGACCCCC	Reverse primer used to generate M3 DNA by site-directed mutagenesis
Tth17	TAATACGACTCACTATAGGGCCCGAAAGCCGACGCCTGTGGC	Forward primer for PCR amplification of *Tth* 1 template
Tth20	TAATACGACTCACTATAGGGAAGCCGCGTGCCTCCAGAAATGGGG	Forward primer for PCR amplification of *Tth* 2 template
Tth7	TGATCGTCAACGTCCCCTCGGGTAC	Reverse primer for PCR amplification of *Tth* 1 and *Tth* 2 templates

Ara1	TAATACGACTCACTATAGGGTTGCCTACCCATGAATCAGACACAC	Forward primer for PCR amplification of *Ara* template
Ara2	GTCTATGAAGTTGCCAAACTTCATCCTCAAACCAC	Reverse primer for PCR amplification of *Ara* template
Tin1	TAATACGACTCACTATAGGCCTAGCCAGCCGACTGCACGAATATC	Forward primer for PCR amplification of *Tin* template
Tin2	TTGTCACTTCACATCACACGCGCTCTCCCG	Reverse primer for PCR amplification of *Tin* template
Pna1	TAATACGACTCACTATAGGGCTGCCAGGCCCCTCAACTCTC	Forward primer for PCR amplification of *Pna* 1 template
Pna2	TAATACGACTCACTATAGGGCCGCCAAGCCCTCGACTTTC	Forward primer for PCR amplification of *Pna* 2 template (also used for RT-PCR with *P. naphthalenivorans* total RNA)
Pna3	GCGCCAACCGGTGAGGTCATCACTTTG	Reverse primer for PCR amplification of *Pna* 2 template (also used for RT-PCR with *P. naphthalenivorans* total RNA)
Pna4	GTCCACTTCGGCACACCTTTGACTCCAACTTG	Reverse primer for PCR amplification of *Pna* 1 template
Pna5	CGTCGGGATCTACCGAAATGGGGATCACG	Forward primer used for RT-PCR with *P. naphthalenivorans* total RNA
Pna6	TACTGCGCTTGGAAGCGCCGCCGTC	Reverse primer used for RT-PCR with *P. naphthalenivorans* total RNA
Pna8	GCTACGACGAGCGCTGGGCGATGAAATTCG	Forward primer used for RT-PCR with *P. naphthalenivorans* total RNA
Pna9	CGAATTTCATCGCCCAGCGCTCGTCGTAGC	Reverse primer used for RT-PCR with *P. naphthalenivorans* total RNA
