# Supplementary material for: Natural circularly permuted group II introns in bacteria produce RNA circles
Source: iScience. 2021 Nov 13;24(12):103431. doi: 10.1016/j.isci.2021.103431 (PMC8637638; doi:10.1016/j.isci.2021.103431)
Supplement: Data S1. Locations and sequence alignments of CP group II RNAs, related to Figure 1 [file mmc2.pdf]

Taxa, gene contexts and multiple-sequence alignment of permuted group II introns: Naturally occurring, circularly permuted group II introns in bacteria produce RNA circles

Adam Roth, Zasha Weinberg, Koen Vanderschuren, Mitchell H. Murdock, and Ronald R. Breaker

Note: the presentation and explanation text of this supplementary data on novel RNA motifs follows the pattern of our presentation of previously found conserved RNA motifs (Weinberg, *et al.*, 2010).

Contents

1 permuted-group-II **2**
1.1 Taxa 2
1.2 Gene contexts 2
1.3 Conserved domains 4
1.4 Multiple-sequence alignment 5

1 permuted-group-II

1.1 Taxa

The taxonomy of each organism containing a putative permuted-group-II RNA is listed, with abbreviations identifying each hit (e.g., "Eco-1-1" and "Eco-1-2" might hypothetically represent two distinct RNAs in *E. coli*). The abbreviations will be used to identify each individual permuted-group-II RNA in Sections 1.2 and 1.4:.

abbrev. of hits	taxonomy of species
Taq-1-1 to Taq-1-2	Bacteria Deinococcus-Thermus Deinococci Thermales Thermaceae *Thermus aquaticus* Y51MC23
Tos-1-1	Bacteria Deinococcus-Thermus Deinococci Thermales Thermaceae *Thermus oshimai* JL-2
Tth-1-1	Bacteria Deinococcus-Thermus Deinococci Thermales Thermaceae *Thermus thermophilus*
Tth-2-1 to Tth-2-2	Bacteria Deinococcus-Thermus Deinococci Thermales Thermaceae *Thermus thermophilus* HB8
Gsp-1-1	Bacteria Firmicutes Bacillales Bacillaceae *Geobacillus sp.* WCH70
Gdi-1-1	Bacteria Proteobacteria Alphaproteobacteria Rhodospirillales Acetobacteraceae *Gluconacetobacter diazotrophicus* PAl 5
Rpi-1-1	Bacteria Proteobacteria Betaproteobacteria Burkholderiales Burkholderiaceae *Ralstonia pickettii* 12D
Rpi-2-1	Bacteria Proteobacteria Betaproteobacteria Burkholderiales Burkholderiaceae *Ralstonia pickettii* 12J
Rpi-3-1	Bacteria Proteobacteria Betaproteobacteria Burkholderiales Burkholderiaceae *Ralstonia pickettii* OR214
Rsp-1-1	Bacteria Proteobacteria Betaproteobacteria Burkholderiales Burkholderiaceae *Ralstonia sp.* 5_2_56FAA
Rsp-2-1	Bacteria Proteobacteria Betaproteobacteria Burkholderiales Burkholderiaceae *Ralstonia sp.* 5_7_47FAA
Rsp-3-1	Bacteria Proteobacteria Betaproteobacteria Burkholderiales Burkholderiaceae *Ralstonia sp.* AU12-08
Cte-1-1	Bacteria Proteobacteria Betaproteobacteria Burkholderiales Comamonadaceae *Comamonas testosteroni* KF-1
Pna-1-1	Bacteria Proteobacteria Betaproteobacteria Burkholderiales Comamonadaceae *Polaromonas naphthalenivorans* CJ2
Tin-1-1	Bacteria Proteobacteria Betaproteobacteria Burkholderiales *Thiomonas intermedia* K12
Lni-1-1	Bacteria Proteobacteria Betaproteobacteria Neisseriales Neisseriaceae *Lutiella nitroferrum* 2002
Ajo-1-1	Bacteria Proteobacteria Gammaproteobacteria Pseudomonadales Moraxellaceae *Acinetobacter johnsonii* SH046
Ara-1-1	Bacteria Proteobacteria Gammaproteobacteria Pseudomonadales Moraxellaceae *Acinetobacter radioresistens* SH164
env-1 to env-51	environmental samples

1.2 Gene contexts

Each permuted-group-II RNA (indicated by "RNA→") is listed. For each hit, the downstream genes predicted to reside in a regulated operon are listed. If the nearest downstream gene is encoding in the opposite strand (and therefore presumed to not be a part of a regulated operon), then that gene is still depicted. Some environmental sequences and some RefSeq entries lack gene annotations, and so no genes are available for such sequences. The direction of each gene is indicated with an arrow (→), and each predicted conserved domain in the gene is named. Conserved domains associated with more than one permuted-group-II RNA are assigned a color; other domains are gray. Information about these conserved domains is given in Section 1.3. The accession of the sequence containing each permuted-group-II RNA is given in the column named "Seq. accession". Accessions beginning with "NC_", "NS_", "NW_" or "NZ_" are contained in RefSeq. Other accession refer to environmental samples. Nucleotide coordinates are given for the 5′ and 3′ boundaries of each permuted-group-II RNA. If the 5′ coordinate is greater than the 3′ coordinate, the RNA is present on the reverse-complement strand of the containing genomic DNA sequence. Each hit is denoted by an abbreviation (like "Eco-1-1") that refers to a taxonomy given in Section 1.1.

abbrev.	Seq. accession		5′ at	3′ at	genes
Gsp-1-1	NC_012790.1	+	6758	7479	RE_MjaII (pfam09520)→ hypo→ hypo→ hypo→ **RNA**→ hypo→ hypo→ hypo→ TTHC007 (TTHC007)→ hypo→ hypo→ hypo→
Ara-1-1	NZ_GG705138.1	+	384	1149	hypo→ hypo→ hypo→ **RNA**→ hypo→ hypo→ hypo→ hypo→ hypo→ Rep_3 (pfam01051)→
env-1	saf2_Contig13228	+	1	605	**RNA**→ hypo→ INT_P4 (cd00801)INT_REC_C (cd01182)bSCRAP90 (bSCRAP90)→ ←hypo
Ajo-1-1	NZ_GG704978.1	-	15389	14607	←hypo hypo→ hypo→ hypo→ hypo→ **RNA**→ (big gap) hypo→ ←hypo
Pna-1-1	NC_008761.1	+	83	834	hypo→ hypo→ **RNA**→ hypo→ hypo→ ←KfrA_N (pfam11740)
Lni-1-1	NZ_ACIS01000012.1	+	3950	4715	hypo→ hypo→ hypo→ hypo→ hypo→ hypo→ **RNA**→ ←hypo ←hypo

Gdi-1-1	NC_010124.1	+	23126	23958	←CpaF (COG4962) ←TTHC007 (TTHC007) ←hypo ←hypo hypo→ hypo→ **RNA**→ hypo→ TrwB_AAD_bind (pfam10412)SXT_TraD (TIGR03743)→ TrwB_AAD_bind (pfam10412)SXT_TraD (TIGR03743)→
Tth-2-1	NC_006461.1	-	975195	974287	←INT_REC_C (cd01182)bSCRAP90 (bSCRAP90) ←excise (TIGR01764) ←hypo ←TTHC007 (TTHC007) hypo→ hypo→ hypo→ **RNA**→ hypo→ ←hypo ←hypo
Tth-2-2	NC_006463.1	-	5976	5075	DUF2283 (pfam10049)→ ←hypo ←hypo ←hypo ←TTHC007 (TTHC007) hypo→ hypo→ hypo→ **RNA**→ hypo→ hypo→ ←hypo ←hypo
env-2	BISONR_BXAS706_y2	-	650	15	hypo→ hypo→ **RNA**→ hypo→
env-3	bisonPool14jan08_C6916	+	120	727	hypo→ hypo→ **RNA**→ hypo→
env-4	bisonPool14jan08_C6203	+	888	1067	←hypo hypo→ hypo→ **RNA**→ hypo→
env-5	BISONR_C5754	-	2425	2029	hypo→ hypo→ **RNA**→ hypo→ DUF433 (pfam04255)→
env-6	2014180340	+	86	380	hypo→ **RNA**→ hypo→
env-7	BISONQ_BXBH23756_b1	-	523	8	hypo→ hypo→ **RNA**→ hypo→
env-8	BISONP_C17150	+	858	1447	←TTHC007 (TTHC007) hypo→ hypo→ hypo→ **RNA**→
env-9	BISONP_FGGH27985_b1	+	1	780	**RNA**→
env-10	BISONR_C3523	-	1447	1136	hypo→ **RNA**→ hypo→ ←hypo
env-11	BISONR_C4792	+	1863	2033	←hypo ←hypo ←TTHC007 (TTHC007) ←hypo hypo→ hypo→ **RNA**→ hypo→
env-12	BISONR_C3582	-	1792	807	hypo→ hypo→ hypo→ **RNA**→ hypo→ hypo→
env-13	BISONR_C5102	+	529	1531	hypo→ hypo→ hypo→ **RNA**→ hypo→ hypo→
env-14	2014180950	+	1	144	**RNA**→ hypo→
env-15	2014173460	-	1004	74	**RNA**→ hypo→
env-16	2014178903	+	65	371	hypo→ **RNA**→ hypo→
env-17	bathPlank_BWBY8398_b1	+	1	615	**RNA**→ hypo→
env-18	bathPlank_BWBY3698_b1	+	1	659	**RNA**→ hypo→ hypo→
env-19	bathPlank_C1512	-	2836	2007	**RNA**→ hypo→ hypo→ ←hypo
env-20	bathPlank_C1513	-	177	1	←TTHC007 (TTHC007) ←hypo ←hypo hypo→ hypo→ **RNA**→
env-21	2013581011	-	727	115	hypo→ hypo→ **RNA**→ hypo→
env-22	2015251844	+	297	759	hypo→ hypo→ **RNA**→
Tos-1-1	NC_019388.1	+	23898	24743	←hypo ←hypo ←hypo hypo→ hypo→ **RNA**→ (big gap) hypo→ hypo→
Taq-1-1	NZ_ABVK02000019.1	+	12609	13454	←hypo ←hypo ←hypo hypo→ hypo→ ←hypo hypo→ hypo→ hypo→ **RNA**→ hypo→ hypo→ hypo→
env-23	BISONR_C5857	+	11383	12228	←hypo ←hypo ←TTHC007 (TTHC007) hypo→ hypo→ hypo→ **RNA**→ hypo→ hypo→ hypo→
env-24	ADKJ01000634.1	+	2097	2942	←hypo ←hypo ←hypo hypo→ hypo→ **RNA**→ (big gap) hypo→ hypo→
env-25	2014170290	+	266	714	hypo→ hypo→ **RNA**→ hypo→
env-26	bathPlank_C5834	+	1	788	**RNA**→ hypo→ hypo→ ←hypo hypo→
Tth-1-1	NC_007190.1	-	8512	7656	←hypo ←hypo ←TTHC007 (TTHC007) hypo→ hypo→ hypo→ ←hypo ←ABC_cobalt_CbiO_domain1 (cd03225) ←hypo ←hypo ←hypo DUF2442 (pfam10387)→ ←Replicase (pfam03090)PriCT_1 (pfam08708) ←hypo ←RelE (COG2026) ←COG4710 (COG4710) hypo→ **RNA**→ hypo→ ←hypo ←ABC_cobalt_CbiO_domain1 (cd03225)
env-27	BISONR_C4444	-	7762	6903	hypo→ hypo→ hypo→ **RNA**→ hypo→ ←hypo hypo→ ABC_cobalt_CbiO_domain1 (cd03225)→
env-28	JGI20131J14367_1002023	+	61	920	hypo→ hypo→ **RNA**→ hypo→ hypo→
env-29	bisonPool05dec07_BXBC33452_x2	-	558	3	←TTHC007 (TTHC007) hypo→ hypo→ **RNA**→ hypo→
env-30	BISONR_C1275	+	75	957	hypo→ hypo→ hypo→ **RNA**→ hypo→ hypo→
Taq-1-2	NZ_ABVK02000020.1	+	13319	14155	←Replicase (pfam03090)PriCT_1 (pfam08708) ←hypo ←hypo ←hypo ←hypo ←hypo hypo→ hypo→ hypo→ **RNA**→ hypo→ hypo→ hypo→ hypo→
env-31	2015254855	-	464	177	hypo→ hypo→ hypo→ **RNA**→ hypo→
env-32	BISONP_FGGH2987_x2	-	227	54	←TTHC007 (TTHC007) hypo→ hypo→ **RNA**→ hypo→
env-33	bathPlank_BWBY21907_b1	-	534	16	hypo→ hypo→ **RNA**→ hypo→
env-34	BISONR_C3739	-	2626	1795	hypo→ hypo→ hypo→ **RNA**→ hypo→ hypo→ ←hypo hypo→ ←hypo
env-35	BISONR_C4443	-	2840	2010	←TTHC007 (TTHC007) ←hypo hypo→ hypo→ **RNA**→ hypo→ ←hypo
env-36	bisonPool14jan08_BXAW28174_y3	+	669	801	←TTHC007 (TTHC007) hypo→ hypo→ **RNA**→
env-37	bisonPool05dec07_BXBC34878_x3	+	422	835	←TTHC007 (TTHC007) hypo→ hypo→ **RNA**→
env-38	BISONR_C3740	-	773	6	←hypo ←TTHC007 (TTHC007) hypo→ hypo→ **RNA**→
env-39	2015285099	-	667	154	hypo→ hypo→ **RNA**→ hypo→
env-40	bathPlank_BWBY22078_b1	-	476	47	←TTHC007 (TTHC007) ←hypo hypo→ hypo→ **RNA**→ hypo→
env-41	bisonPool05dec07_BXBC34694_x3	-	869	584	hypo→ hypo→ **RNA**→ hypo→

env-42	BISONQ_C9446	+	43	811	hypo→ hypo→ hypo→ **RNA**→ hypo→ hypo→ hypo→
env-43	BISONP_FGGH30115_g1	-	375	95	hypo→ **RNA**→ hypo→
env-44	BISONQ_BXBH15739_b1	+	13	823	hypo→ hypo→ **RNA**→
env-45	2015268708	+	194	479	hypo→ **RNA**→ hypo→
env-46	BISONQ_C10386	-	1014	178	hypo→ hypo→ hypo→ **RNA**→ hypo→
Cte-1-1	NZ_AAUJ02000001.1	+	5993950	5994757	←Transposase_7 (pfam01526) hypo→ hypo→ **RNA**→ ←thiolase (cd00751) ←pcaJ_scoB_fam (TIGR02428)
Rpi-2-1	NC_010683.1	+	11051	11813	hypo→ hypo→ **RNA**→
Rpi-3-1	NZ_APMQ01000029.1	+	13497	14259	Rep_3 (pfam01051)→ ←hypo ←hypo hypo→ hypo→ **RNA**→ hypo→ VirB4 (COG3451)TrwB_AAD_bind (pfam10412)→
Rsp-3-1	NZ_ASZV01000064.1	-	3561	2799	hypo→ hypo→ **RNA**→
env-47	JGI12006J14256_100191	-	4507	3745	hypo→ hypo→ hypo→ hypo→ **RNA**→ ←hypo hypo→ VirB4 (COG3451)TrwB_AAD_bind (pfam10412)→
env-48	JGI12010J14255_102123	-	1948	1186	hypo→ hypo→ hypo→ hypo→ **RNA**→ ←hypo hypo→ hypo→
env-49	JGI12146J14260_101207	-	3598	2836	hypo→ hypo→ hypo→ hypo→ **RNA**→ ←hypo hypo→ VirB4 (COG3451)TrwB_AAD_bind (pfam10412)→
env-50	JGI11877J14254_100152	-	3438	2676	←hypo ←hypo hypo→ hypo→ hypo→ hypo→ **RNA**→ ←hypo hypo→ VirB4 (COG3451)TrwB_AAD_bind (pfam10412)→
env-51	JGI12087J14258_101206	-	3598	2836	**RNA**→
Rsp-1-1	NZ_JH815241.1	+	10841	11603	hypo→ hypo→ **RNA**→
Rsp-2-1	NZ_GL520226.1	-	127969	127207	Rep_3 (pfam01051)→ ←hypo ←hypo hypo→ **RNA**→ (big gap) VirB4 (COG3451)TrwB_AAD_bind (pfam10412)→
Rpi-1-1	NC_012851.1	+	6297	7059	hypo→ hypo→ **RNA**→
Tin-1-1	NZ_ACXV01000048.1	+	3821	4496	Pilin (pfam00114)→ SR_ResInv (cd03768)HTH_Hin_like (cd00569)→ ←hypo ←hypo hypo→ **RNA**→ ←hypo ←Replicase (pfam03090)PriCT_1 (pfam08708)

1.3 Conserved domains

Conserved domains found in protein-coding genes listed in Section 1.2 are shown below, with the first sentence in their description from the Conserved Domain Database (if any). Conserved domains associated with more than one permuted-group-II RNA are assigned a color, while others are shown in gray. The number in parentheses after the colored domain name is the number of occurrences in Section 1.2.

u bSCRAP90 (2) Self-Cleaving Ribozyme Associated Protein number 90 (gi=270157735 by jackhmmer)
u cd00569 (1) Helix-turn-helix domain of Hin and related proteins, a family of DNA-binding domains unique to bacteria and represented by the Hin protein of Salmonella.
u cd00751 (1) Thiolase are ubiquitous enzymes that catalyze the reversible thiolytic cleavage of 3-ketoacyl-CoA into acyl-CoA and acetyl-CoA, a 2-step reaction involving a covalent intermediate formed with a catalytic cysteine.
u cd00801 (1) Bacteriophage P4 integrase.
u cd01182 (2) DNA breaking-rejoining enzymes, intergrase/recombinases, C-terminal catalytic domain.
u cd03225 (3) Domain I of the ABC component of a cobalt transport family found in bacteria, archaea, and eukaryota.
u cd03768 (1) Serine Recombinase (SR) family, Resolvase and Invertase subfamily, catalytic domain; members contain a C-terminal DNA binding domain.
u COG2026 (1) Cytotoxic translational repressor of toxin-antitoxin stability system [Translation, ribosomal structure and biogenesis / Cell division and chromosome partitioning]
u COG3451 (5) Type IV secretory pathway, VirB4 components [Intracellular trafficking and secretion]
u COG4710 (1) Predicted DNA-binding protein with an HTH domain [General function prediction only]
u COG4962 (1) Flp pilus assembly protein, ATPase CpaF [Intracellular trafficking and secretion]
u pfam00114 (1) Pilin (bacterial filament).
u pfam01051 (3) Initiator Replication protein.
u pfam01526 (1) Transposase.
u pfam03090 (3) Replicase family.
u pfam04255 (1) Protein of unknown function (DUF433).
u pfam08708 (3) Primase C terminal 1 (PriCT-1).
u pfam09520 (1) MjaII restriction endonuclease.
u pfam10049 (1) Protein of unknown function (DUF2283).
u pfam10387 (1) Protein of unknown function (DUF2442).
u pfam10412 (7) Type IV secretion-system coupling protein DNA-binding domain.
u pfam11740 (1) Plasmid replication region DNA-binding N-term.
u TIGR01764 (1) DNA binding domain, excisionase family.
u TIGR02428 (1) 3-oxoacid CoA-transferase, B subunit.
u TIGR03743 (2) conjugative coupling factor TraD, SXT/TOL subfamily.
u TTHC007 (16) TTHC007 protein

1.4 Multiple-sequence alignment

Each permuted-group-II RNA is denoted by an abbreviation (like "Eco-1-1") that refers to a taxonomy given in Section 1.1. The alignment may include sequences containing the RNA motif, as well as flanking sequence. The permuted-group-II RNA itself is denoted by the line underneath marked 5′ and 3′ on either end. Nucleotides in flanking sequences (i.e., not part of the motif) are written in gray letters. Stems of predicted rho-independent transcription terminators, if any, are shaded yellow. (Note: terminator predictions have not been analyzed manually, and many are likely to be false positives.) Nucleotides predicted to function (as DNA) as transcription-factor binding sites, if any, are shaded in green. (But note: these predictions are manually annotated, so they might be under-predicted.) Annotated start codons, if any, are shaded green. (Note: start codons are frequently misannotated, especially in environmental samples.) Nucleotides proposed to basepair as part of the consensus structure are shaded in color when they comprise Watson-Crick or G-U pairs. Otherwise they are shaded gray. Conserved stems are also indicated at the bottom of the alignment by angle brackets, where matching $<$ and $>$ denote base-paired columns. Below these angle brackets, the symbol "2" denotes base pairs exhibiting covariation, "1" denotes base pairs exhibiting compatible mutations, "0" denotes base pairs that are not observed to mutate and "?" denotes base pairs that have a significant frequency of non-canonical nucleotides for Watson-Crick or G-U pairs ($> 5\%$). Below these base pair annotation is the consensus sequence: "R" = "A" or "G", "Y" = "C" or "U", red nucleotides: nucleotide identity conserved more than 97% of the time, black nucleotides: 90%, gray nucleotides: 75%, red circle (•): nucleotide is present 97% of the time, black circle (•): 90%, gray circle (•): 75%, white circle (◦): 50%. All percentages of sequences just described (e.g. 97% conserved) assume that sequences have been weighted by the GSC algorithm implemented by the Infernal software package.

Duplicate sequences: the following putative homologs are not shown in the alignment because their sequences are identical to a homolog already shown: Rpi-1-1, Rpi-3-1, Rsp-2-1, Rsp-3-1, Taq-1-1, env-23, env-24, env-28, env-47, env-48, env-49, env-50, env-51

alignment positions 1 · · · 180

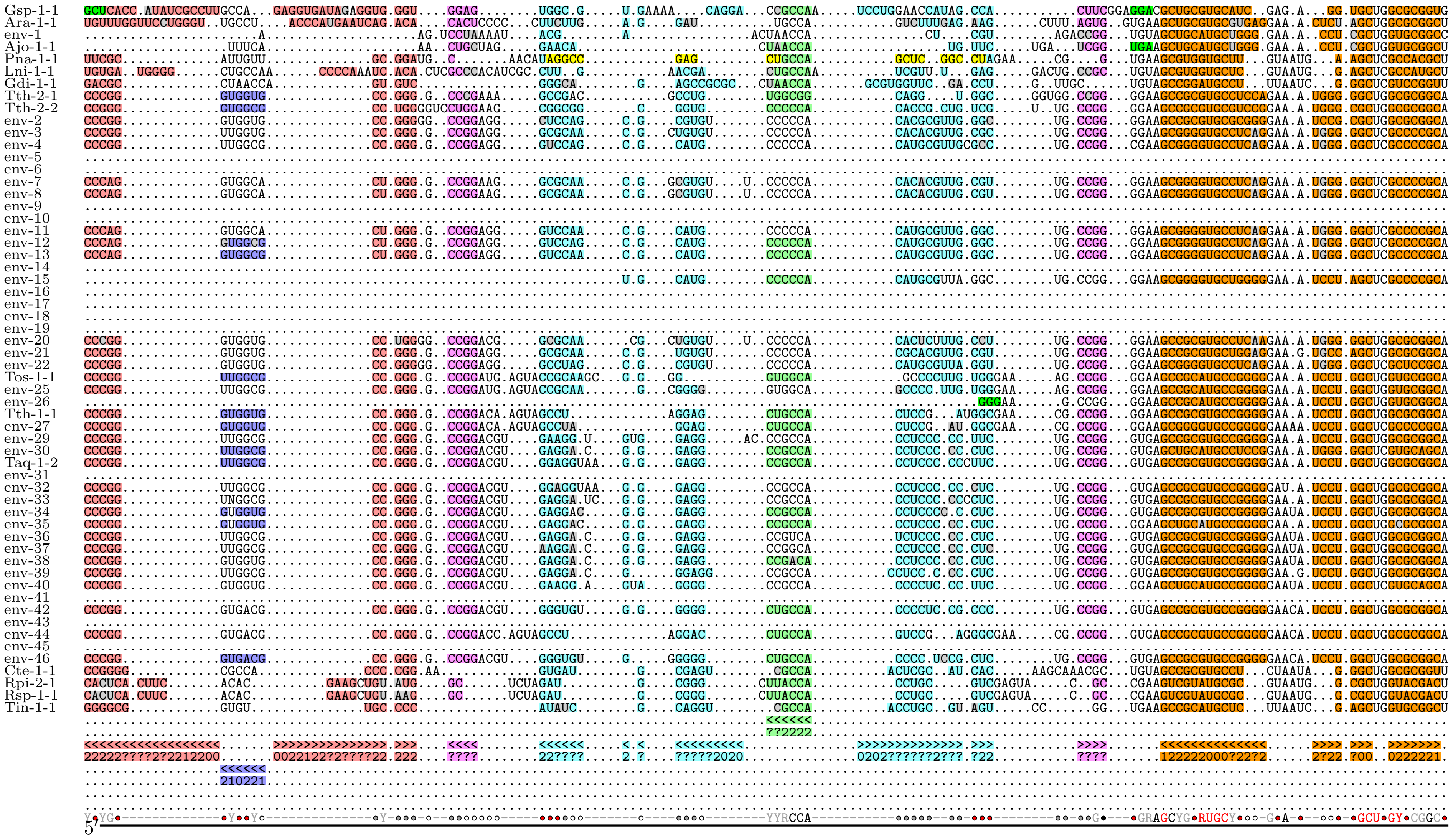

alignment positions 181 · · · 360

```
Gsp-1-1   U.CAA.C.GGGGGCU.GCGA.G.....UAA........................CAUCGC.UUCUACCC..GA.UG..GGUUA................................................................................UGCGUA.UAUGGGCGAC
Ara-1-1   A.CA..CUAGGGGCUGGCGG.G..UGUGACU.......................C.CCGCUUUCUACCUG.U..UC...................ACUCAUUUUGAU....GAAUGAACACCCUCCACUCCCAA.................GGUGGA.......GGUCA.GUUGGGCGAC
env-1     GCGAA...GGGGGCUCGAGA.GU..GAAAU.......................UC.UCUCGUUCUACCC..AA.UC..UAAUUC....CCUUAUU.UUGA...................................................AUUAAG........GGUU.GUUGGGCGAC
Ajo-1-1   A.CAA.C.GGGGGCUAAAGA.GU..GAAAU.......................UC.UCUUUUUCUACCC..AA.UC..AAAAAA....UUGAAC............................................................AAGAU...UAAAGUU.GUUGGGCGAC
Pna-1-1   A.CGA.CG.AGCGCUGGG...CGAUGAAAU......................UCG...CCCUUCGACU.C.AA.UC..CCUCUUUU..UCAUGGG.UGCU.G.................................................CCCAUGA........GA...UUGGGCGAC
Lni-1-1   G.CGA.UGGUGCGCUGGGGG.GU..GAAAU.......................UC.CCUCCUUCGAC.CC.AA.UC..CAU..............GCAUAACUGAUGGA......................................................UAUGAG.ACUGGGCGAC
Gdi-1-1   G.CAA.C.GGGCGCUCAG...GGGUGAAAC......................CCCC..CUGUUCGACCC..AA.UC..ACCGCCUU..CCGCACGGGCGA.CCGUGCCGGUCUUUGACAAUCGAAUACGAUUUAG.................AAGCACA..CCAAAAGAA.GUUGGGCGAC
Tth-2-1   CUCAA.CGGGGGGCUCCGGG.CGA.UAGAG......................ACG.UCCGGGUCUAUCCC.AA.UG..CGACAA....CCCCGA..GGGA.....................................................UCGGGG...GUUUGGAG.GAAGGGCGAC
Tth-2-2   CUCAA.CGCGGGGCCUGC.A.GGCUGAGAG......................GCC.U.GCGGUCUAUCGC.AA.UC..CAAA......CCCCGA..GGGA.....................................................UCGGGGAUCUGUAGGAG.GACGGGCGAC
env-2     CUCAA.CGGGGAGCGCGGUGCGCU.GAGA.......................AGC.CGCCGCUCGAUCCC.GA.UC..CACAUA....CCUGGGG.GGGA....................................................CUCCGGG...GAUUGACGGUUGGGCGAC
env-3     CUCAA.CGGGGAGCGCGGCG.GCU.GAGA.......................GGC.CGCCGCUCGAUCCC.GA.UC..CACGUCCAACCCGGGG..GGGA.....................................................CCCCGG...GGAUGAUGGUUGGGCGAC
env-4     CUCAA.CGAGGCGCGUGGAG.GCC.GAGA.......................GGC.CUCCGCUCGACCUC.AC.UC..CAUA......CCUGGG..GGGA.....................................................CCCAGG...GCCAGAU.GUUGGG....
env-5     ....................................................................................................................................................................................
env-6     ....................................................................................................................................................................................
env-7     CUCAA.CGCGGCGCU.GGGG.....UAACC..........................CCCC.UUCGACCGC.AC.UC..CAA......CCCGAG..GGGA.....................................................CUCGGG...GCUAGAC.GUUGGGCGAC
env-8     CUCAA.CGCGGCGCU.GGGG.....UAACC..........................CCCC.UUCGACCGC.AC.UC..CAA......CCCGAG..GGGA.....................................................CUCGGG...GCUAGAC.GUUGGGCGAC
env-9     .........GGCGCU.GGGG.....UAACC..........................CCCC.UUCGACCGC.AC.UC..CAA......CCCGAG..GGGA.....................................................CUCGGG...GCUAGAC.GUUGGGCGAC
env-10    ....................................................................................................................................................................................
env-11    CUCAA.CGCGGCGCU...GG.GG..UAACC.......................CC.CC...UUCGACCGU.AC.UC..CAA......CCCGAG..GGGA.....................................................CUCGGG...GCUAGAC.GUUGGG....
env-12    CUCAA.CGCGGCGCU...GG.GG..UAACC.......................CC.CC...UUCGACCGC.AC.UC..CAA......CCCGAG..GGGA.....................................................CUCGGG...GCUAGAC.GUUGGGCGAC
env-13    CUCAA.CGCGGCGCU...GG.GG..UAACC.......................CC.CC...UUCGACCGC.AC.UC..CAA......CCCGAG..GGAA.....................................................CUCGGG...GCUAGAC.GUUGGGCGAC
env-14    ....................................................................................................................................................................................
env-15    CUCAA.CGGGGAGCGUGGUG.CGCUGAGAG......................GC..CGCCACUCGAUCCC.GA.UC..CACACAUA..CCCGGGG.GGGA....................................................CCCUGGG.GAUGAUUGAUGGUUGGGCGAC
env-16    ....................................................................................................................................................................................
env-17    ....................................................................................................................................................................................
env-18    ....................................................................................................................................................................................
env-19    ..CAA.CGAGGCGC.GUGGA.GGCCGAGAG......................GCC.UCCGCUUCGACCUC.AC.UC..CAUA......CCUGGG..GGGA.....................................................CCCAGG...GCUAGAU.GUUGGGCGAC
env-20    CUCAA.CGGGGUGCGCGAU..CGCUGAAAA......................GUG..GUCGCUCGAUCCCCAA.UC..CUCAUA...CCCGGGG.GGGA....................................................CUCCCGG...GG................
env-21    CUCAA.CGGGGAGCGCGAUG..GCUGAGAG......................GC..UGUCGCUCGAUCCC.GA.UCCGCACAUA...CCUGGGG.GGGAU...................................................CCCCAGG..AAUGAGACGGUUGGGCGAC
env-22    CUCAA.CGGGGAGCGCGAUG..GCUGAGAG......................GC..UGUCGCUCGAUCCC.GC.UCC.AACAUA...CCCGGGG.GGGA....................................................CUCCGGG...GAGUGAUGGUUGGGCGAC
Tos-1-1   CUCAA.CGCGGCGCG.CCCA.GG..CACAG.......................CCUCGGG.UUCGACCGC.AC.UC..CACAU....CCCCGA..GGGA.....................................................UCGGGG...GCCAGACGGUUGGGCGAC
env-25    CUCAA.CGCGGCGCGCCC.A.GG..CACAG.......................CCUC.GGGUUCGACCGC.AC.UC..CACAU....CCCCGA..GAGA.....................................................UCGGGG...GCCAGACGGUUGGGCGAC
env-26    CUCAA.CGCGGCGCGCCC.A.GG..CACAG.......................CCUC.GGGUUCGACCGC.AC.UC..CACAU....CCCCGA..GGGA.....................................................UCGGGG...GCCAGACGGUUGGGCGAC
Tth-1-1   CUCAA.CGCGGCGCUCCGG.GGG..CACAG.......................CCU.CCGGUUCGACCGC.AA.UC..CACAUA...CCCCGA..GGGA.....................................................UCGGGG.....GCGAG.AUUGGGCGAC
env-27    CUCAA.CGCGGCGCU.CCGG.GG.GCACAG.......................CCUCCGG.UUCGACCGC.AA.UC..CACAUA...CCCCGA..GGGA.....................................................UCGGGG.....GCGAG.AUUGGGCGAC
env-29    C.CAAACGCGGCGCG.UCGG.GG..CACAG.......................CCGCCGG.CUCGACCGC.AA.UC..CAA......CCUGGG..GGGA.....................................................CCCAGG...GCUCGAU.GUUGGGCGAC
env-30    C.CAAACGCGGCGCG.UCGG.GG..CACAG.......................CCGCCGG.CUCGACCGC.AA.UC..CAA......CCUGGG..GGGA.....................................................CCCAGG...GCUCGAU.GUUGGGCGAC
Taq-1-2   C.CAAACGCGGCGCG.UCGG.GG..CACAG.......................CCGCCGG.CUCGACCGC.AA.UC..CACAUA...CCUGGG..GGGA.....................................................CCCAGG.....GGUAG.GUUGGGCGAC
env-31    ....................................................................................................................................................................................
env-32    C.CACACGCGGCGCU.GGAG.GG...ACA........................CC.CUCC.UUCGACCGC.AC.UC..CA.......CCUGGA..GGGA.....................................................UCCAGG...GCUAGAC.GUUGGG....
env-33    C.CAAACGCGGCGCUGGAGG.G...GACA.........................C.CCUCCUUCGACCGC.AC.UC..CAA......CCCGAG..GGGA.....................................................CUCGGG...GCUAGAC.GUUGGGCGAC
env-34    C.CAAACGCGGCGCU.GGAG.GG..GACA........................CC.CUCC.UUCGACCGC.AC.UC..CA.......CCUGGA..GGGA.....................................................UCCAGG...GCUAGAC.GUUGGGCGAC
env-35    C.CAAACGCGGCGCU.GGAG.GG..GACA........................CC.CUCC.UUCGACCGC.AC.UC..CA.......CCUGGA..GGGA.....................................................UCCAGG...GCUAGAC.GUUGGGCGAC
env-36    C.CAAACGCGGCGCU.GGAG.G...GCAC.........................C.CUCC.UUCGA..................................................................................................................
env-37    C.CAAACGCGGCGCU.GGAG.G...GCAC.........................C.CUCC.UUCGACCGC.AC.UC..CA.......CCUGGA..GGGA.....................................................UCCAGG...GCUAGACGGAAGGGCGAC
env-38    C.CAAACGCGGCGCU.GGAG.GG..GACA........................CC.CUCC.UUCGACCGC.AC.UC..CAA......CCCGAG..GGGA.....................................................CUCGGG...GCUAGAC.GUUGGGCGAC
env-39    C.CAAACGCGGCGCUGGAGG.G...GACA.........................C.CCUCCUUCGACCGC.AC.UC..CAA......CCUGGG..GGGA.....................................................CCCAGG...AGAUGACGGUUGGGCGAC
env-40    CUCAA.CGCGGCGCUGGAGG.G...GACA.........................C.CCUCCUUCGACCGC.AC.UC..CAUA......CCCGGG..GGAA.....................................................CCCGGG...GCCAGAC.GUUGGGCGAC
env-41    ....................................................................................................................................................................................
env-42    C.CAAACGCGGCGCU.GGGA.GG..GAGA........................CC.UCCC.UUCGACCGC.AC.UC.GCAA......CCCGAG..GGGA.....................................................CUCGGG...GUUCGAC.GUUGGGCGAC
env-43    ....................................................................................................................................................................................
env-44    C.CAAACGCGGCGCUGGAGG.....ACAC...........................CCUCCUUCGACCGC.AC.UC.GCAA......CCCGAG..GGGA.....................................................CUCGGG...GUUAGAC.GUUGGGCGAC
env-45    ....................................................................................................................................................................................
env-46    C.CAAACGCGGCGCUGGAGG.....ACAC...........................CCUCCUUCGACCGC.AC.UC.GCAA......CCCGAG..GGGA.....................................................CUCGGG...GUUAGAC.GUUGGGCGAC
Cte-1-1   G.CGAA..GGGCGCUGGUGA.GUG...CAA......................CUC.UCACCUUCGACCC..AA.UC..CAU.......CUUGCGGCUCAACCCCGCAAGAUCAUCGCCAGACCGCUGGCGGCGUACUGAGUGACAAACGAGGCAAAAC..CAAAUUGAA.GUUGGGCGAC
Rpi-2-1   A.CGA.CGAG.CGCUGGACG.GUG.UAA........................CUC.CGUCCUUCGA.CUC.AA.UC..........CCC....GGGA.........................................................GGG.....GCGAG.UUUGGGCGAC
Rsp-1-1   A.CGA.CGAG.CGCUGGACG.GUG.UAA........................CUC.CGUCCUUCGA.CUC.AA.UC..........CCC....GGGA.........................................................GGG.....GCGAG.UUUGGGCGAC
Tin-1-1   A.CGU.CGAG.CGCU.GCGG.GGU.GAAAA......................UCC.CCGC.UUCGA.CUC.AU.UC..CAGUCU............GCGCCUACUGAAAAGGC................................................GAUUGCAG.AUUGGGCGAC
          ....................................................................................................................................................................................
          ....................................................................................................................................................................................
          .......<<<<<<..<<<<<.<<<............................>>>.>>>>>..>>.>>>>..................<<<<<<<.........................................................>>>>>>>....................<
          .......222120..22222.2??............................??2.22222..02.1222..................???????.........................................................???????....................0
          ....................................................................................................................................................................................
          ....................................................................................................................................................................................
          ....................................................................................................................................................................................
          ....................................................................................................................................................................................
          ●-CRA-C○●GG●GCU○R●●R-G●--●A●A●----------------------●C-Y●●Y○UUC●AYC●○-A●-UC--●R●○○-----YY●○○○--●●●A--------------------------------------------------○●●●R○----○○●●GA●-RUUGGGCGAC
```

alignment positions 361 · · · 540

```
Gsp-1-1   ACAGAAAU.....................................................................................AGUGAUUCCGA.........CC..AUAC................UCGGAAU.UG.CU.CGUCACAUUC....G.AAGUGGG.U....
Ara-1-1   AAGGACACGGG.........AUCACGGUGACGUGG..........CGGUGGGAGUAACUUCCCUCCUAGGCUGAAAG.....GGCCAAAACCC..........................................................UGUAUUAAAACAG.....ACAGU....CA
env-1     AUGGAAAC.....................................................................................GGGAG..CUGUG........GUGA...................CACAGACCAAC.CC.UGUUUUAACU....G.AA.GGUAAGCAAU
Ajo-1-1   GUAGACAC.....................................................................................GG.GCGCUUUG.........GUGA....................CAAAGCA.CAACCCUGUUUUAAUU....G.AAGUCAG.ACUUG
Pna-1-1   GCAGAAAC.....................................................................................GGGCAGCAUG..........AGGAAAUG.................CGUGCU.GUGCC.UGGUACAGGU...CG.AAAUCCGGAAA..
Lni-1-1   ACGGAAAC.....................................................................................GGCGCACGGA..........AGGAAAUG.................ACCGUG.CGACC.UGUCGCAGGG...UG.AAAGCAGG.....
Gdi-1-1   GUGGACAC.....................................................................................GGCGCGCCCUC.CCCUCGGUUGGC...........ACCGAGGGCGAGGGCGCUG.CC.UGCCAGAUAGA...A.GAAUGGG.U....
Tth-2-1   GCGGGAAG.....................................................................................GGUCGCCGGGG....GGGGCGUAG...........GCCCCGGAAACCCGGC.GG.CU..GGCCCGUGUUGGG....AGUGGG.U...
Tth-2-2   CCGGACAG.....................................................................................GUCGGGCCU...........GAAA......................UGGCC.CG.GC.UGUCUGCCCC....G.AAAGUGGGAGAG
env-2     CCGGACAG.....................................................................................GUCAGA..............GAGGAGGUG...................CUC.UG.GC.UGUCCGUCCC....G.AAAGCGGGGUAGG
env-3     CCGGAGUG.....................................................................................GUCAGGGCU...........GAAA......................CGCUC.UG.GC.UGUCCGUCCC....G.AAAGCGGGGUAGG
env-4     ....................................................................................................................................................................................
env-5     ....................................................................................................................................................................................
env-6     ....................................................................................................................................................................................
env-7     CCGGACAG.....................................................................................GUCUGGCCGUCAGCG.....CAUCUCUAGCCAUCGCGCA.UGCUGGCCGUU.AG.GC.UGUCGGCUCC....G.AAAGCGG......
env-8     CCGGACAG.....................................................................................GUCUGGCCGUCAGCG.....CAUCUCUAGCCAUCGCGCA.UGCUGGCCGUU.AG.GC.UGUCGGCUCC....G.AAAGCGGGGA...
env-9     CCGGACAG.....................................................................................GUCUGGCCGUCAGCG.....CAUCUCUAGCCGUCGCGCA.CGCUGGCCGUU.AG.GC.UGCCGGCUCC....G.AAAGCGG......
env-10    ....................................................................................................................................................................................
env-11    ....................................................................................................................................................................................
env-12    CCGGACAG.....................................................................................GUCUGGCCGUCAGC......GCAC.................GCUGGCCGUC.AG.GC.UGCCGGCUCC....G.AAAGCGGGG..GG
env-13    CCGGACAG.....................................................................................GUCUGGCCGUCAGCG.....CAUCUCUAGCCAUCGCGCA.UGCUGGCCGUC.AG.GC.UGCUGGCUCC....G.AAAGCGGGG..GG
env-14    ....................................................................................................................................................................................
env-15    CCGGACAG.....................................................................................GUCGCGACU...........GAAA......................UGUCG.CG.GC.UGCCUGUUCC....G.AAAGUGGGGUCGG
env-16    ....................................................................................................................................................................................
env-17    ....................................................................................................................................................................................
env-18    ....................................................................................................................................................................................
env-19    CCGGAAAG.....................................................................................GUCGGGGCU...........GCGA......................UGCUC.CG.GC.UGUCCGUCCC....G.AAAGCGGGGUAGG
env-20    ....................................................................................................................................................................................
env-21    CCGGACAG.....................................................................................GUCAGGGCU...........GCGA......................CGCCC.CG.GC.UGCGUGUUCC....G.AAAGUGGGGUCGG
env-22    CCGGACAG.....................................................................................GUCGGGGCU...........GCGA......................UGCUC.CG.GC.UGCCUGUUCC....G.AAAGUGGGGGCGG
Tos-1-1   GCGGAAAG.....................................................................................GGUCCCAGGGG.........GGUCAUAA................CCCCUGG.GG.CU..GGGCCUGG.....G.AAAGUGGCU....
env-25    GCGGAAAG.....................................................................................GGUCCCAGGGG.........GGUCAUAA................CCCCUGG.GG.CU..GGGCCUGG.....G.AAAGUGGCU....
env-26    GCGGAAAG.....................................................................................GGUCCCAGGGG.........GGUCAUAA................CCCCUGG.GG.CU..GGGCCUGG.....G.AAAGUGGCU....
Tth-1-1   CCGGAAAG.....................................................................................GGUCCUGGGGG.........CG...AA.................CCCCCAG.GG.CU..GACCGCGG.....G.AAAGUGGCU....
env-27    CCGGAAAG.....................................................................................GGUCCUGGGGG.........GUCAA.AA................CCCCCAG.GG.CU..GACCGCGG.....G.AAACCCGCU....
env-29    CCGGAAAG.....................................................................................GGUCCUCGGGG.........CG...AA.................CCCCGAG.GG.CU..GAGCGUGG.....G.AAACCCGCU....
env-30    CCGGAAAG.....................................................................................GGUCCUCGGGG.........CCA..AA.................CCCCGAG.GG.CU..GAGCGUGG.....G.AAACCCGCU....
Taq-1-2   CCGGAAAG.....................................................................................GGUCCUCGGGG.........CG...AA.................CCCCGAG.GG.CU..GAGCGUGG.....G.AAACCCGCU....
env-31    ....................................................................................................................................................................................
env-32    ....................................................................................................................................................................................
env-33    GCGGAAAG.....................................................................................GGUCCUCGGGG.........CG...AA.................CCCCGAG.GG.CU..GAGCGUGG.....G.AAAGGGGCU....
env-34    GCGGAAAG.....................................................................................GGUCCCUAGGG.........CG...AA.................CCCUAGG.GG.CU..GAGCGUGG.....G.AAAGGGGCU....
env-35    GCGGAAAG.....................................................................................GGUCCCUAGGG.........CG...AA.................CCCUAGG.GG.CU..GAGCGUGG.....G.AAAGGGGCU....
env-36    ....................................................................................................................................................................................
env-37    GCGGAAAG.....................................................................................GGUCCUCGGGG.........CG...AA.................CCCCGAG.GG.CU..GAGCGUGG.....G.AAAGGGGCU....
env-38    GCGGAAAG.....................................................................................GGUCCUCGGGG.........CG...AA.................CCCCGAG.GG.CU..GAGCGUGG.....G.AAAGGGGCU....
env-39    GCGGAAAG.....................................................................................GGUCCUCGGGG.........CG...AA.................CCCCGAG.GG.CU..GAGCGUGG.....G.AAAGGGGCU....
env-40    GCGGAAAG.....................................................................................GGUCCUCGGGG.........CG...AA.................CCCCGAG.GG.CU..GAGCGUGG.....G.AAAGGGGCU....
env-41    ....................................................................................................................................................................................
env-42    GCGGACAG.....................................................................................GGUUUCCAGGG.........CCG..AA.................CCCUGGG.GG.CU..GAGCCUGG.....G.AAAGGGGCU....
env-43    ....................................................................................................................................................................................
env-44    GCGGACAG.....................................................................................GGUCCCCAGGG.........CCG..AA.................CCCUGGG.GG.CU..GGGCCAGU.....G.AACGGGGCU....
env-45    ....................................................................................................................................................................................
env-46    GCGGACAG.....................................................................................GGUCCCCAGGG.........CCG..AA.................CCCUGGG.GG.CU..GGGCCAGU.....G.AACGGGGCU....
Cte-1-1   CGUGAAAC.....................................................................................GG.CGCUGGGC.........AGGAAAUG................GCCCAGU.GACCU..GGUCAAUGGU...GAAAGUGGG.U....
Rpi-2-1   ACGGAAAC.....................................................................................GGG..CUCAGCG........AGGAAAUG...............CGCUGAGU.UC.CU..GUGUAGCGU....G.AAAGCGGUA....
Rsp-1-1   ACGGAAAC.....................................................................................GGG..CUCAGCG........AGGAAAUG...............CGCUGAGU.UC.CU..GUGUAGCGU....G.AAAGCGGUA....
Tin-1-1   ACGGAAAC.....................................................................................GGG..CUCGGC.........AGGAAAUG................GCCGAUU.AC.CC.UGUGUUGG......A.UAUGGACGGGA..
          ....................................................................................................................................................................................
          ....................................................................................................................................................................................
          ....................................................................................................................................................................................
          ....................................................................................................................................................................................
          <<<..................<<<<<....>>>>>...........<<<<<<<....>>>>>>>....<<<<..........>>>>.......<<<<<<<<<<<<<<<<<<<<...............>>>>>>>>>>>>>>>>.>>.>>...<<<<<<<<<<<......<<<<<<<<<<
          222..................00000....00000...........00?000?....?000?00....0000..........0000.......22????22?22????2????...............????2????22?22??.??.22...2222?22?220......22????????
          ....................................................................................................................................................................................
          ....................................................................................................................................................................................
          ●YGGA●A●-------------------------------------------------------------------------------------GG●●●●●●●●○---------●●●○--○○----------------○●●●R●●-●●-CY-○G●●●●●●●○----G-AAR●●RG○●----
```

alignment positions 541 · · · 720

```
Gsp-1-1  ...............GCAA.............................AUCCCACCUAGGGGGAGUUGG..............GAAA.........................CC.GCUCAACCU..A..GCGCGG.CGGGG.GAGGU.UGCG............................
Ara-1-1  .GAGG..ACUCA...CGGAA............UGAGUAACCUCGUGAAUAGCUGAUAACCAGACGCUUCAAGUC.........AAAAACAAAAAUAAA.........GACAA.GGGCGU.AGGU..UAUACCCAC.AGGUGGGCUUU.UGAG............................
env-1    A...AGACACAA...GGA..............UUGUGUUUGAGCGUUAAAGGCCC.CUUUUUAUCAUGA..............GCGA.........................UUUUGAUAUGAA..GG.CUAGGC.AGGUGGGCGAC.UGAAAU..........................
Ajo-1-1  ...............AGAU.........................C.ACGGAUGAUU.UCAACGCUAAAAUGAC...UGC....CUUUU..............GCAUGAGUGAUUUU.GCGAUGG.GCA.CAGCAC.AGGUGGGCGAC.UAAAAG....................UGA..G
Pna-1-1  .ACUG..........AGG.....................CAGUCAG..UCCGGACG.UCGGGAUCUACC..............GAA...AUG....................GG..GAUCACGA.UUG.CCACAC.ACGUAGGCUUC.UGAG............................
Lni-1-1  ...............UGAUCAGU..........................CCUGCC.UAUGGCCGAUACC..............GAAA.........................GG.GUUGGACAU.AUG.CCAGAC.ACGUAGGCUAC.UGAG............................
Gdi-1-1  ...............AAUCA............................GUCCGAUCCACGGGCGGC.AA..............GGGA.........................UU.GUCGAACGU.GUG.CCAAGC.AGGUGGGUGGC.AGCA............................
Tth-2-1  ...............GAAA............................ACCCCACC.CGUGGGCAGGGGGG..AGUCCGCCCGGGCGA...........CCGGG.UAGCUGUACCCUCUGCACAC..GG.CGCCGC.ACGCCACGGC..AU..............................
Tth-2-2  GGCGUAGCCC.....CAGA..............GGGCGUAGCCCUA..CCCCGCCGCCGGUGGGU.GGG..............GAAAUCG.G.AA.................CCC.ACCUGCCG.GUG..CCCCGCUGGCGCAGCGC.UGAGG.CCGGCGGCGAGACGCCGGGUGUCGGU
env-2    GGGUGGUGUCUA...CAUCAACAUCAUCUACAUAGACAUCAC..CUUCCUCCGCCGCCGGUGAUC.GGG..............GAAACGCAG.AA.................CCC.GGUUGCCG.GUG..CCCCACAGGCGCGGCGC.UGAGG.CCCACGAACAAACGUGGGGUGUCCG.
env-3    G..............GGCUAUG.....................CCUUCCCCCGCCGCCGGUAGCC.GGG..............GAAAGAA.GGAA.................CCC.GGUUGCCG.GUG..CCCCACAGGCCCAGCGC.UGAGG.CCCGUGAAUAGACACGGGGUGUCGGU
env-4    ....................................................................................................................................................................................
env-5    ....................................................................................................................................................................................
env-6    ....................................................................................................................................................................................
env-7    ...............GGAUC..............................CCGCCUCCGG..UCUGGGG..............GAAA.........................CCCUAGG..CCG.GUG...CCCACAGGCCCAGCGC.UGAGG.CCCGUGAAGGAACACGGGGUGUCGGU
env-8    ................................................UCCCGCCUCCGG..UCUGGGG..............GAAA.........................CCCUAGG..CCG.GUG...CCCACAGGCCCAGCGC.UGAGG.CCCGUGAAGGAACACGGGGUGUCGGU
env-9    ...............GGAUC..............................CCGCCUCCGG..UCUGGGG..............GAAA.........................CCCUAGG..CCG.GUG...CCCACAGGCCCAGCGC.UGAGG.CCCGUGAAGGAACACGGGGUGUCGGU
env-10   ....................................................................................................................................................................................
env-11   ....................................................................................................................................................................................
env-12   GAUGUCCC.......UAUGG...............GGGGAUGUCCCUAUCCCGCCUCCGG..UCUGGGG..............GAAA.........................CCCUAGG..CCG.GUG...CCCACAGGCCCAGCGC.UGAGG.CCCGUGAAGGAACACGGGGUGUCGGU
env-13   GAUGUCCC.......UAUGG...............GGGGAUGUCCCCAUCCUGCCUCCGG..UCUGGGG..............GAAA.........................CCCUAGG..CCG.GUG...CCCACAGGCCCAGCGC.UGAGG.CCCGUGAAGGAACACGGGGUGUCGGU
env-14   ....................................................................................................................................................................................
env-15   GGAUGGUGCG.....GUAUC..............CGCAUCGUCUUC..CCCCACCGCCGGUGAUC.GGG..............AAAACGC.GGAA.................CCC.GGUUGCUG.GUG..CCCCACGGGUGCGGCGC.UGAGG.CCCGUGAAUAAACACGGGGUGUCGG.
env-16   ....................................................................................................................................................................................
env-17   .....................................................................................................................................................................ACACGGGGUGUCGGU
env-18   .........................................................UCGGGAAA.GAA..............GGAACCC.G.AA........................UCGCCGGUG..CCCCACAGGCCCAGCGC.UGAGG.CCCGUGAAUAAACACGGGGUGUCGGU
env-19   G..............GGCCUUGUG...................CCUUCCCCCGCCGCCGGUGAUC.GGG..............GAAAGAA.GGAA.................CCC.GGUUGCCG.GUG..CCCCACAGGCCCAGCGC.UGAGG.CCCGUGAAUAGACACGGGGUGUCGGU
env-20   ....................................................................................................................................................................................
env-21   GGAUGG.........UGGUAU.................CCAUCUUC..CCCCACCGCCGGUGAUC.GGG..............GAAACACAG.AA.................CCC.GGUUGCCG.GUG..CCCCACGGGCGCAGCGC.UGAGG.CCCACGAACAAACGUGGGGUGUCAU.
env-22   G..............GGCCUUGUG...................CCUUCCCCCACCGCCGGUGAUC.GGG..............GAAACGC.G.AAC................CCC.GGUUGCCG.GUG..CCCCACUGGCGCGGCAC.UGA.G.CCCACGAAUAGACGUGGGGUGUCCGG.
Tos-1-1  ...............GCGA.............................GUCCCACCCGCGGCGGGGAGU..............GAACCCAUA....................GCUCCCCGACGC.GUG.CGGAGC.UGGCC.GGUGC.UGAG............................
env-25   ...............GCGA.............................GUCCCACCCGCGGCGGGGAGU..............GAACCCAUA....................GCUCCCCGACGC.GUG.CGGAGC.UGGCC.GGUGC.UGAG............................
env-26   ...............GCGA.............................GUCCCACCCGCGGCGGGGAGU..............GAACCCAUA....................GCUCCCCGACGC.GUG.CGGAGC.UGGCC.GGUGC.UGAG............................
Tth-1-1  ...............GCAA.............................GUCCCACCCACGGGAUGGGGU..............GAACCCAUA....................GCCCCAUCACGU.GUG.CGGAGC.AGGCC.GGUGC.UGAG............................
env-27   ...............GAAA.............................GUCCGGGCCACGGGAUGGGGU..............GAACCCAUA....................GCCCCAUCACGU.GUG.CGGAGC.AGGCC.GGUGC.UGAG............................
env-29   ...............GCGA.............................GUCCGGGCCACGGCAUGGAGG..............GAACCCAUA....................CCUCUAUGACGU.GUG.CGGAGC.ACGCC.GGUGC.UGAG............................
env-30   ...............GCGA.............................GCCGGG.CCACGGCAUGGAGG..............GAACCCAUA....................CCUCUAUGACGU.GUG.CGGAGC.ACGCC.GGUGC.UGAG............................
Taq-1-2  ...............GCGA.............................GUCCGGGCCACGGCAUGGGGU..............GAACACAUA....................GCCCCAUGACGU.GUG.CGGAGC.ACGCC.GGUGC.UGAG............................
env-31   ....................................................................................................................................................................................
env-32   ....................................................................................................................................................................................
env-33   ...............GCGA.............................AUCCCCCCCGCGGCAUGGGGC..............GAACCCAUA....................GCCCCAUGACGC.GUG.CGGCGG.CGGCC.GGUGG.UCAU............................
env-34   ...............GCAA.............................GUCCCCCCCGCGGCCUGGGGC..............GAACCCAUA....................GCCCCAUGACGC.GUG.CGGCGG.CGGCC.GGUGG.UCAU............................
env-35   ...............GCAA.............................GUCCCCCCCGCGGCCUGGGGC..............GAACCCAUA....................GCCCCAUGACGC.GUG.CGGCGG.CGGCC.GGUGG.UCAU............................
env-36   ....................................................................................................................................................................................
env-37   ...............GCGA.............................AUCCCCCCCGCGGCAUGGGGC..............GAACCCAUA....................GCCCCAUGACGC.GUG.CGGCGG.CGGCC.GGUGG.UCAU............................
env-38   ...............GCGA.............................AUCCCCCCCGCGGCCUGGGGC..............GAACCCAUA....................GCCCCAUGACGC.GUG.CGGCGG.CGGCC.GGUGG.UCAU............................
env-39   ...............GCGA.............................AUCCCCCCCGCGGCGUGGGGC..............GAACCCAUA....................GCCCCAUGACGC.GUG.CGGCGG.CGGCC.GGUGG.UCAU............................
env-40   ...............GCAA.............................GUCCCCCCCGCGGGAUGGGGC..............GAACCCAUA....................GCCCCAUGACGC.GUG.CGGCGG.CGGCC.GGUGG.UCAU............................
env-41   ....................................................................................................................................................................................
env-42   ...............GCGA.............................AUCCCCCCCGCGGGAUGGGGC..............GAACCCAUA....................GCCCCAUGACGC.GUG.CGGCGG.CGGCC.GGUGG.UGAU............................
env-43   ....................................................................................................................................................................................
env-44   ...............GCGA.............................GUCCCCCUCACGGGACGGGGU..............GAAUCUAUA....................GCCCUGUGACGU.GUG.CGGAGC.AGGCC.GGUGC.UGAG............................
env-45   ....................................................................................................................................................................................
env-46   ...............GCGA.............................GUCCCCCUCACGGGACGGGGU..............GAAUCUAUA....................GCCCUGUGACGU.GUG.CGGAGC.AGGCC.GGUGC.UGAG............................
Cte-1-1  ...............GAAA.............................GACCGACCGGUGGGGCGUAUC..............GAAA.........................GA.GCGCAACAC.CUG.CCGCAC.AGGAUGGCUUC.UGAG............................
Rpi-2-1  .GACGGUG.......GCUGUCUG.............CACCGUCGUUAGCACCGCC.CAGGGGCUGCGG...............GAGA..........................CCGUAGAACCU.GUG.CCACAC.ACGUAGGCUGC.UGAG............................
Rsp-1-1  .GACGGUG.......GCUGUCUG.............CACCGUCGUUAGCACCGCC.CAGGGGCUGCGG...............GAGA..........................CCGUAGAACCU.GUG.CCACAC.ACGUAGGCUGC.UGAG............................
Tin-1-1  ...GAUGC.......GCAA.................GCAUCGUUAACACCG.CC...UCGGCAUGUAUC..............GCAA.........................GA.GCGUGACGA.GUG.CCGCAG.AUGAGGG...C.UUAG............................
         ....................................................................................................................................................................................
         ....................................................................................................................................................................................
         <<<<<<<<<<<<<<<..............>>>>>>>>>>>>>>>>>>>>>>>>>...<<<.<<<<<<<<<<<<<<<<<<<<<<...............>>>>>>>>>>>>>>>>>>>>>>.>>>....<<<<<<<.<<<<<..<<<<.......<<<<<<......>>>>>>..<<<<<<
         ????2???22220???..............???02222??2????????????22...222.?2222?22???2?????00000...............00000?????2???22?2222?.222....?22222?.221??..??22.......002220......022200..222???
         ....................................................................................................................................................................................
         ....................................................................................................................................................................................
         ....................................................................................................................................................................................
         ....................................................................................................................................................................................
         ---------------R●R●-------------------------○---●●CY●●Y○●●●GR●●●●●R●●--------------GRR●-------------------------●●○●●●Y●AC●●-●UG-●●●●R●-R●GY●○G●●●C-UGAR----------------------------
```

alignment positions 721 · · · 900

```
Gsp-1-1  ........................GCA...........................GUAA..........................UGCUUGCA........................................................................................
Ara-1-1  ........................GGCA.UC.......................GUGA.......................GGUGUC.AGGGUUGC...AAG...............GC........GAAA............GC..UGGC..GCAAUCGGU.ACUC.GA.UUUUA.GGU
env-1    ........................UUAG.CC.......................GAAA.......................GGU.GA.UCAAGACA...GAG...............AAAAACUGUCAGAU....GUCAGAUUUAAA.AAA..GGUCAUGGACAUC....GGGUA.CUU
Ajo-1-1  CC....GAAA.....GGCGAUCAAGACA.G........................AGAAAAA.....................CUGUC.AGAUGUCA...GA................UU........UUAA............AA...GGG..U..CAUGGACAUC....GGGUA.CUU
Pna-1-1  ........................GAUG.UU.......................GUAA.......................GGCAUC.AGGGCAUC...GAG...............GG........GAAA............CC..UGGA..CAUGCUGGC.ACCU.GG.......A..
Lni-1-1  ........................GGCG.CA.......................GUAA.......................UGCGUC.AGGCCGCA...GAG...............GG........GAAA............CC..UGGA..AGCCGCGGC.ACCU.CG.......G..
Gdi-1-1  ........................UGGC.GG.......................GAAA.......................CCGCCAUGAGUCUCA...GAG...............GG........GAAA............CC..UGGA..UGGCAUGGU.ACCA.GG.G.....GGC
Tth-2-1  ........................GGGC.CUC.C....................GUGA.....................G.GGGCUC...GGAACCA....................C.........CCUGG............G........GGA.CCCCCUCCU.GG.C.....UUC
Tth-2-2  CGUGC.GGCC..GCACGACCGGC.UGGG.CC.......................GUAA.......................GGCCCC.AGGCCGCC...GA..........................G....................GGA..GGCGGUGGC.ACAG.GGGGGGA..AGC
env-2    .....GGAAA.C......CCGGC.UGGA.CC.......................GCAAA......................GGUCCC.AGGCCCCC...AA................GG........GUAAAA..........CC...GGC..GGGGUGGC.A.CA.CGGGGGA..UGU
env-3    UGCGGGGAAA.CCCGCG.CCGGC.UGGA.CC.......................GCGA.......................GGUCCC.AGGCUCCC...GA.................G........GCUU............C....GGA..GGGAGUGGC.ACAG.GAGGGGA..GG.
env-4    ....................................................................................................................................................................................
env-5    ....................................................................................................................................................................................
env-6    ....................................................................................................................................................................................
env-7    CGCGGGGAAAACCCGCG.CCGGC.UGGA.CC.......................GUAA.......................GCUCCC.AGCCUCCC...GA................GG........GCGA............CU...GGA..GGGAGGGGC.ACAG.GGGGGGA..GGC
env-8    CGCGGGGAAAACCCGCG.CCGGC.UGGA.CC.......................GUAA.......................GCUCCC.AGCCUCCC...GA................GG........GCGA............CU...GGA..GGGAGGGGC.ACAG.GGGGGGA..GGC
env-9    UGCGGGGAAA.CCCGCG.CCGGC.UGGA.CC.......................GCAA.......................GCUCCC.AGCCUCCC...GA................GG........GCGA............CU...GGA..GGGAGGGGC.ACAG.GAGGGGA..GGC
env-10   ....................................................................................................................................................................................
env-11   ....................................................................................................................................................................................
env-12   UGCGGGGAAA.CCCGC..CCGGC.UGGA.GUAAGCGCUAGGUGCCGGCGUUGGGGAUAACCCAC.CCGGCUCC.AGCGUGAGCUCCC.AGCCUCCC...GA................GG........GCGA............CU...GGA..GGGAGGGGC.ACAG.GGGAGGA..GGC
env-13   UGCGGGGAAA.CCCGC..CCGGC.UGGA.GUAAGCGCUAGGUGCCGGCGUUGGGGAUAACCCAC.CCGGCUCC.AGCGUGAGCUCCC.AGCCUCCC...GA................GG........GCGA............CU...GGA..GGGAGGGGC.ACAG.GGGAGGA..GGC
env-14   ....................................................................................................................................................................................
env-15   .....GGAAA.C......CCGGC.UGGA.CC.......................GCGA.......................GGUCCC.AGGCUCCC...GA................GG........GUAG............CC...GGA..GGGAGUGGC.ACGG.GAGGGGA..GGC
env-16   ....................................................................................................................................................................................
env-17   CGCGGGGAAA.CCCGCG.CCGGC.UGGA.CC.......................GCGA.......................GGUCCC.AGGCUCCC...GA.................G........GCUU............C....GGA..GGGAGUGGC.ACAG.GAGGGGA..GG.
env-18   UGCGGGGAAA.CCCGCG.CCGGC.UGGA.CC.......................GCGA.......................GGUCCC.AGGCUCCC...GA.................G........GCUU............C....GGA..GGGAGUGGC.ACAG.GGG.GGA..GGC
env-19   CGCGGGGAAA.CCCGCG.CCGGC.UGGA.CC.......................GCGA.......................GGUCCC.AGGCUCCC...GA.................G........GCUU............C....GGA..GGGAGUGGC.ACAG.GAGGGGA..GG.
env-20   ....................................................................................................................................................................................
env-21   CGG...GUAA....CCG.AUGGC.UGGG.GC.......................GUGA.......................GCCCCC.AGGCUCCC...GA................GG........GCAA............CC...GGA..GGGAGUGGC.ACAG.GAGGGGA..GAC
env-22   .....GAAAA.C......CCGGC.UGGA.CC.......................GCGA.......................GGUCUC.AGGCUCCC...GA................GG........GUAA............CC...GGA..GGGAGUGGC.ACAGAGGG......AGC
Tos-1-1  ........................GGUG.UC.......................GCAA.......................GGCACC.AGGGGGCC...GA................GG........GAGA............CU...GGA..GCCCCCGCUGACCC.GG.GAAGCC...
env-25   ........................GGUG.UC.......................GCAA.......................GGCACC.AGGGGGCC...GA................GG........GAGA............CU...GGA..GCCCCCGCUGACCC.GG.GAAGCC...
env-26   ........................GGUG.UC.......................GCAA.......................GGCACC.AGGGGGCC...GA................GG........GAGA............CU...GGA..GCCCCCGCUGACCC.GG.GAAGCC...
Tth-1-1  ........................GGUG.CC.......................GCGA.......................GGCACC.AGGGGGCU...GA................GG........GAGA............CU...GGA..GCCCCCGCUGACCA.GG.GAAAA.CGG
env-27   ........................GGUG.CC.......................GCGA.......................GGCACC.AGGGGGCU...GA................GG........GAGA............CU...GGA..GCCCCCGCUGACCA.GG.GAAAA.CGG
env-29   ........................GUCG.CC.......................GCGA.......................GGCGAC.AGGGGGCU...GA................GG........GAGA............CU...GGA..AGCCCCGCUGACCA.GG.GAAAA.CGG
env-30   ........................GUUG.CC.......................GCGA.......................GGCAAC.AGGGGGCU...GA................GG........GAGA............CU...GGA..AGCCCCGCUGACCA.GG.GAAAA.CGG
Taq-1-2  ........................GUUG.CC.......................GCGA.......................GGCAAC.AGGGGGCU...GA................GG........GAGA............CU...GGA..GGCCCUGCU.ACCA.GG.G........
env-31   ....................................................................................................................................................................................
env-32   ....................................................................................................................................................................................
env-33   ........................GCGC.UCC......................GUGA.......................GGGCGU.AGGCCCAG...GG................GG........GAGA............CC...UGC..CUGGGCGCCCACCC..UG.........
env-34   ........................GCGC.UU.......................GCGA.......................GGGCGU.AGGCCCAG...GG................GG........GAGA............CC...UGC..CUGGGCGCCCACCC..UG.........
env-35   ........................GCGC.UC.......................GCGA.......................GGGCGU.AGGCCCAG...GG................GG........GAGA............CC...UGC..CUGGGCGCCCACCC..UG.........
env-36   ....................................................................................................................................................................................
env-37   ........................GCGC.UC.......................GCGA.......................GGGCGU.AGGCCCAG...GG................GG........GAGA............CC...UGC..CUGGGCGCCCAUCC..UG.........
env-38   ........................GCGC.UC.......................GCGA.......................GGGCGU.AGGCCCAG...GG................GG........GAGA............CC...UGC..CUGGGCGCCCACCC..UG.........
env-39   ........................GCGC.UC.......................GCGA.......................GGGCGU.AGGCCCAG...GG................GG........GAGA............CC...UGC..CUGGGCGCCCACCC..UG.........
env-40   ........................GCGC.UU.......................GCGA.......................GGGCGU.AGGCCCAG...GG................GG........GAGA............CC...UGC..CUGGGCGCCCACCC..UG.........
env-41   ....................................................................................................................................................................................
env-42   ........................GCGC.CC.......................GUGA.......................GGGCGU.AGGCUCGG..GGU................GG........GAGA............CC...UGC..CCGAGCGCCCACCC..UG.........
env-43   ....................................................................................................................................................................................
env-44   ........................GGUG.CC.......................GCGA.......................GGUACC.AGGGGGCU...GA................GC........GCGA............GU...GGA..GCCCCCGCUGACCC.GG.GAAUUC...
env-45   ....................................................................................................................................................................................
env-46   ........................GUCG.UC.......................GUGA.......................GGCGAC.AGGGGGCU...GA................GC........GCGA............GU...GGA..GCCCCCGCUGACCC.GG.GAAUUC...
Cte-1-1  ........................GUAC.CG.......................GUGA.......................CGGUAC.AGAACGCG...GAG...............GG........GAAA............CC..UGGA..AGCCAGGGC.ACCU.CG.......G..
Rpi-2-1  ........................GCGU.UCC......................GAAA......................GGG.CGC.AGGCGUGC...AAG...............GG........GAAA............CC..UGGC..GCACGCGGC.ACCU.AC.......CGG
Rsp-1-1  ........................GCGU.UCC......................GAAA......................GGG.CGC.AGGCGUGC...AAG...............GG........GAAA............CC..UGGC..GCACGCGGC.ACCU.AC.......CGG
Tin-1-1  ........................CACG.CCA......................GAAA......................UGGCGUG.A.UGGCGU...GA.UGUCCGCGAGGACGGCUGG.CCG.AGCGA....U.CGGAACAGACGAGG..ACGCCAGGC.ACCU.GGG.........
         ....................................................................................................................................................................................
         ....................................................................................................................................................................................
         <<<<<<.....>>>>>>>>>>>>.<<<<.<<<.<<<<<<<<<<<<<<<<..<<<.....>>>.>>>>>>>>>>>>>>>>.>>>>>>>...<<<<<<<<....<<<<<....>>>>>.<<<<<<<<.<........>.>>>>>>>>.....>>>>>>>>><<<..<<<.<<<......<<<
         122020.....020221???222.?222.222.20000?00?00000?0..0??.....??0.0?00000?00?00002.222222?...??2?2???....00000....00000.222??020.2........2.020??222.....???2?2???222..222.220......222
         ....................................................................................................................................................................................
         ....................................................................................................................................................................................
         ....................................................................................................................................................................................
         ....................................................................................................................................................................................
         ------------------------●●●●-Y○-----------------------G●RA-----------------------○G○●●C-AGR●●●●●---GA----------------○○--------G●RR------------○○---RG○--●○○○●YG●Y-ACY○-○○-○-----○○○
```

alignment positions 901 · · · 1080

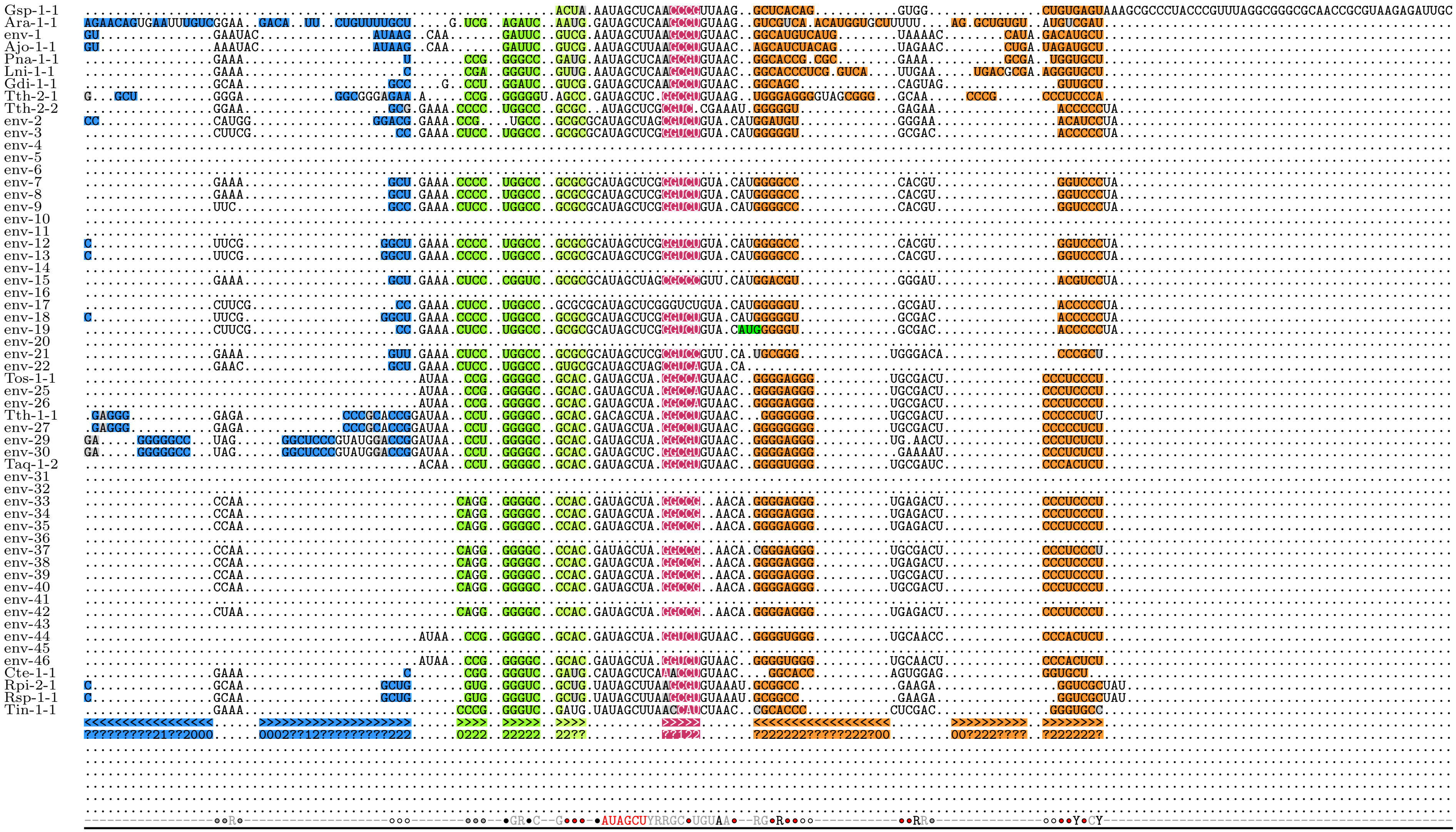

alignment positions 1081 ··· 1260

```
Gsp-1-1  AAAGACGCGAGGCUAAACC.GCGU.AGAAGGCGUAGCGUAGCCGAAAGU.............................................................CU.UGCCA.UAA.GGC.........................AAGAU...GAGUA..AGGG.....GAUGC
Ara-1-1  .................AU.GGGUAAGGCGUGAACGUGGAUGAUU..............................................................................AUUAA..............CUACAUAUUGCAUU...GUAUAAACCUGA....UACGA
env-1    .................GC.CUAG.AAAUUUUGCAUUGCACGGUAAAUCUAAAGA.................................................................GUCUGUAGC.....................CCAUCU...AUAA....CCG.GGA....UC
Ajo-1-1  .................UU.GCUG.AAA.AUUUUGAUA..GCACGGUAA.........................................................................CUCUAAA..............GAGUCUUUAGUCG...GUGUA....AGUGGG....AU
Pna-1-1  .................GU.GUGG.AGC.AAGGAGGAG..CCGUUGUUUGCCCUUGGCAAGUAAUCGGUA.....................................................GUCGAUGA..............AGGAUU.UUUC...GAGUA..ACCCACUG....GC
Lni-1-1  .................GU.CUGG.AGUG.UUGUAGAG................................CUG.CUAUCCAUGUUCAG..CAUGGACAGUCAGG.................G.CGUGAUGA..............UAC.GUUUUCC...CAGUA..ACCGACCA....GC
Gdi-1-1  .................GC.UUGG.AGAGAAAGUGCAAGU..............................GCCCGGGC.....CUACAGU.....GUCCCGGUGGGUAAA.............GUUGGAA...............ACCAUGA.UGU...GAGUA..AGGGAGCG....GC
Tth-2-1  .................UC.GGCG.AGGCAGGAA..CCCUAGGCGCCCGCG...............................................................CUCG.CGU.ACCGCAU......................GCCC......U...ACCC.....GA.GC
Tth-2-2  .................CG.GGG..AAGUACCGGUG.CCGAUGGACGCCA............................................................CG.C.CCG.GCG.CGG.......................GCCCCCC......U...AACC.....GA.GC
env-2    .................UG.GGG..AACAGGACGUGAGCCCACGGGUACUG...........................................................CC.CAUCA.ACUGCUG.......................GUCCUCC......U...AACC.....GA.GC
env-3    .................UG.GGG..AACAGGGCGUGAGCCCACGGGUACUG...........................................................CC.CGCCACACU.CUG.......................GCCCCCC......U...AUCC.....GA.GC
env-4    ....................................................................................................................................................................................
env-5    ..............................................................................................................CC.CGUCU.GCCUCUG.......................GCCCCCC......U...AUCC.....GA.GC
env-6    ....................................................................................................................................................................................
env-7    .................UG.GG...AAGCCAAGUG...CCCACGGGUAAUC............................................................C.CGU................................................................
env-8    .................UG.GG...AAGCCAAGUG...CCCACGGGUAAUC............................................................C.CGUCU.GCCCCUG.......................GCCCCCC......U...AUCC.....GA.GC
env-9    .................UG.GG...AAGCCAAGUG...CCCACGGGUACUG...........................................................CC.CGCCACACU.CUG.......................GCCCCCC......U...AUCC.....GA.GC
env-10   ....................................................................................................................................................................................
env-11   ....................................................................................................................................................................................
env-12   .................UG.GG...AAGCCAAGUG...CCCACGGGUAAU............................................................CC.CGUCU.GCCCCUG.......................GCCCCCC......U...AUCC.....GA.GC
env-13   .................UG.GG...AAGCCAAGUG...CCCACGGGUAAU............................................................CC.CGUCU.GCCCCUG.......................GCCCCCC......U...AUCC.....GA.GC
env-14   ....................................................................................................................................................................................
env-15   .................UG.GGG..AACAGGAUGCGGGCCCACGGGUACUG...........................................................CC.UGCCA.GUUUGCG.......................GUCUUCC......U...AUCC.....GA.GC
env-16   ....................................................................................................................................................................................
env-17   .................UG.GGG..AACAGGCCGUGAGCCCACGGGUACUG...........................................................CC.CGCCACACU.CUG.......................GCCCCCC......U...AUCC.....GA.GC
env-18   .................UG.GGG..AACAGGCCGUGAGCCCACGGGUAGCG...........................................................CC.CAUCA.ACCGCUG.......................GCCCCCC......U...AUCC.....GA.GC
env-19   .................UG.GGG..AACAGGGCGUGAGCCCACGGGUACUG...........................................................CC.CGCCACACC.CUG.......................GCCCCCC......U...AUCC.....GA.GC
env-20   ....................................................................................................................................................................................
env-21   .................AU.GGG.GAACAGAAUGCCAGCGUACGGGUAGCG...........................................................CU.CGCCG.GUUUGCG.......................GCCCCCC......U...AUCU.....GA.GC
env-22   ....................................................................................................................................................................................
Tos-1-1  .................UC.UCCG.AGACAGGCCG....CCUGGGGUC.UGCAACG......................................................CC.CACGC.CG..UGC...........................CCG..GUAGUA..CCCC.....GA.GC
env-25   .................UC.UCCG.AGACAGGCCG....CCU..........................................................................................................................................
env-26   .................UC.UCCG.AGACAGGCCG....CCUGGGGUC.UGCAACG......................................................CC.CACGC.CG..UGC...........................CCG..GUAGUA..CCCC.....GA.GC
Tth-1-1  .................GC.UCCG.AGC.CCCCCG....CCUGGGGUCCGUGAA...........................................................GCCUU.GGC.CGU..........................GCCC..GGAGUA..CCCC.....GA.GC
env-27   .................GC.UCCG.AGC.CCCCCG....CCUGGGGUCCGUGAA...........................................................GUCCU.GGC.CGU..........................GCCC..GGAGUA..CCCC.....GA.GC
env-29   .................GC.UCCG.AGUUGCCCCG....CCUGGGGUCCUG...........................................................CC.CUGCC.GUCCCGU...........................GCC..GGAGUA..UCCC.....GA.GC
env-30   .................GC.UCCG.AGUUGCCCCG....CCUGGGGUCCUG...........................................................CC.CUGCC.GUC.CCG..........................UGCC..GGAGUA..UCCC.....GA.UC
Taq-1-2  .................GC.UCCG.AGUUGCCCCG....CCUGGGGUCCUGC..........................................................CC.UGCCG.UCC.CGU...........................GCC..GUAGUA..CCUC.....GA.GC
env-31   ....................................................................................................................................................................................
env-32   ....................................................................................................................................................................................
env-33   .................UC.GCCG.AGUUGCCCCG....CCUGGGGUCCUGC..........................................................CC.UGCCG.UCC.CGU...........................GCC..GUAGUA..CCCC.....GA.GC
env-34   .................UC.GCCG.AGC.ACCCCG....CCUGGGGUCCUGC..........................................................CC.CGCCG.UCC.CGU...........................GCC..GGAGUA..CCCC.....GA.GC
env-35   .................UC.GCCG.AGCUGCCCCG....CCUGGGGUCCUGC..........................................................CC.CGCCG.UCC.CGU...........................GCC..GGAGUA..CCCC.....GA.GC
env-36   ....................................................................................................................................................................................
env-37   .................UC.GCCG............................................................................................................................................................
env-38   .................UC.GCCG.AGC.ACCCCG....CCUGGGGUCCUGC..........................................................C..CCGCC.UCC.CGU...........................GCC..GUAGUA..CCCC.....GA.GC
env-39   .................UC.GCCG.AGC.ACCCCG....CCUGGGGUCCUGC..........................................................CC.CGCCGUCC..CGU...........................GCC..GGAGUA..CCCC.....GA.GC
env-40   .................UC.GCCG.AGC.ACCCCG....CCU..........................................................................................................................................
env-41   ....................................................................................................................................................................................
env-42   .................UC.GCUG.AGC.AUCCCG....CCUGGAGAUCUG...........................................................CU.CGCCC.UGC.UGU...........................GCC..GUGGUA..CCCC.....GA.GC
env-43   ....................................................................................................................................................................................
env-44   .................GC.UCCG.AGUGAUCCCG....CCUGGAGUCGU............................................................GAAACGUU.GAC.CGU..........................GCCU..GGAGUA..CCUC.....GA.GC
env-45   ....................................................................................................................................................................................
env-46   .................GC.UCCG.AGC.GUCCCG....CCUGGAGAUCU.............................................................GCUCGCC.CUG.CUG..........................UGCC..GUGGUA..CCCC.....GA.UC
Cte-1-1  .................GU.GCGG.AGC.AACGUGGAG................................CCACAGGC.....AUGAA.......GCC.GUGGUUC..............GUAGUCGAUGAGAC...............AAGCGGU...GAGUAA.GGGAAGGG.....C
Rpi-2-1  .................GUGGUGG.GGC.AUCAAGCCU.GUCUGAAGACG...................................................................AAGCU.GUUGAUGGC...................ACAAC...AGGUA..AGCGAGCC....GC
Rsp-1-1  .................GUGGUGG.GGC.AUCAAGCCU.GUCUGAAGACG...................................................................AAGCU.GUUGAAGGC...................ACAAC...AGGUA..AGCGAGCC....GC
Tin-1-1  .................AU.GCGG.AGA.AAGCGACCGACGCCGUUGGCCAGCGUAGCUGGCUAAUCG.................................................G.UAG.CGUGAUGAG..............GAAUCAGACC...AGGUA..AGGGAGGC....GC
         ....................................................................................................................................................................................
         ....................................................................................................................................................................................
         .................>>.>>>>>.............................................<<<<<<<<<<<<<.......>>>>>>>>>>>>>.......<<.<<<<<.<<<.<<<.........<<<<<<<<<<<<<<<<<<<<<..........<<<<<<<<......
         .................?2.2222?.............................................222??2??200000......000002??2??222......??.?????.???.222.........???????0?0????2???222..........??22?222......
         ....................................................................................................................................................................................
         ....................................................................................................................................................................................
         ..........................................<<<<<<....................................................................................................................................
         ..........................................??????....................................................................................................................................
         -----------------●Y-●●●●-AR●○R●●●●R○○○○○○○○○○○○○○○○------------------------------------------------------------------○-○○○-●●Y○○○-------------------○○○●●YY----●●RU○--●●●●-----○○-GC
```

alignment positions 1261 · · · 1440

```
Gsp-1-1  UU.CCAUAUUCGGU.....CCCGUGCC.................................................UAUUUU.....................................GGCACACAA........GC..UACGGCU.GG..AGCGACGUCAGUGCAAUC..........
Ara-1-1  CU.UGGAACUGCGA....UCGGGGGAG.AUA......GGAA........UGU.......................AGGUGUUGUAUGUAG....................................GA........GAU..............CGGCACGGGUUCUGCCAGUUUUCAGGC
env-1    UU..GGAACAACU..UCC.CGG.GGGC.................................................AGAUGG.....................................AC.....AA........GCAGGC..........AGCAGCACCCACAGCGGCCAACAACAUG
Ajo-1-1  CU.UGGAACAACUA.CCCAU...GAAG.CG.CC....AAU.......GGACG........AGACGAGUGU....AACGAGUAAGACUU...............................G......AGCGAGAGCGAGAA...............GCCGGGCAUCCGA............
Pna-1-1  UU.UGGAAUCUCG..CGG.GGGUGAAA.GGUCA....CAUU......UGGCCGU.AGGC...GUA...GCCUA...GAAACAAUCUG.......................................GA........GAC..................GGCGGCGCUUCCAAGCG......
Lni-1-1  UU.UGGAAUCUCG..UGG.CGGUGAAA.GCC......GAAA........GGCGGAGGCCC..UCA..GGGCUACG.GGCAAAUAGUG.......................................GA........GCGC.................GGCAGCGCUACGGCAUG......
Gdi-1-1  UU.UGGAACUUCG..CGC.CCCCCAAA.UUGGCCGUGCAUAG.CGCGGUCGAA..AGGCUCUGA..GAGCCUA...GCGUUCAUGGU.......................................GA........GAC.............GCUUGCGUACUGCUCGU...........
Tth-2-1  UC.CGCAACCUCGUA...GGG.A.................................................AC..GGGC.................UACCGAGGCUUG...CCUCGGU.......CA........GGUGCGCGUG......UCGCGGUAAAGGGGAUAAACCGCCACCU
Tth-2-2  UC.UGGAACCUCGUA....GGGU.....................................................GGGGGA................UCCG...GUUUU.....CGGA.......AA........CCGCGCUGG.GCCG....CUGGUCAGGCGGCCGGG.........
env-2    UCCAGGGACAUCGUA....GGGU.....................................................GGAGUAU...............GCGG...UCAGG.....CCGC.......AA........CAGGGUUGGUG.GG....U.........................
env-3    UCCAGGAACCUCGUA....GGGU.....................................................GGGGGGU...............GCGG...UUA.......CCGC.......AA........CAGGGUUGGCG.GG....U.........................
env-4    ....................................................................................................................................................................................
env-5    U..UGGAACCUCGGA....GGGU.....................................................GGGGGGU...............GCGG...UUA.......CCGC.......AA........CAGGGUUGGCG.GG....UCAUAAGGGCGUACUGGCG.......
env-6    ....................................................................................................................................................................................
env-7    ....................................................................................................................................................................................
env-8    U..UGGAACCUCGUA....GGGU.....................................................GGGGGGU...............GCGG...UCA.......CCGC.......AA........CAGGGUUGGCG.G...............................
env-9    U..UGGAACCUCGUA....GGGU.....................................................GGGGGGU...............GCGG...UCA.......CCGC.......AA........CAGGGUUGGCG.GG....UCAUAAGGGCGUACUGGCA.......
env-10   ....................................................................................................................................................................................
env-11   ....................................................................................................................................................................................
env-12   U..UGGAACCUCGUA....GGGU.....................................................GGGGGGU...............GCGG...UCA.......CCGC.......AA........CAGGGUUGGCG.GG....UCAUAAGGGCGUACUGGCG.......
env-13   U..UGGAACCUCGUA....GGGU.....................................................GGGGGGU...............GCGG...UCA.......CCGC.......AA........CAGGGUUGGCG.GG....UCAUAAGGGCGUACUGGCG.......
env-14   ....................................................................................................................................................................................
env-15   UC.CGGAACCUCGUA....GGGU.....................................................GGAAGGC...............GCGGG..ACCGCAA..CCCGC.......AA........CGCAACUGGUU.GG....UCAUAAGGGCGUGCAUCC........
env-16   ....................................................................................................................................................................................
env-17   UCCAGGAACCUCGUA....GGGU.....................................................GGGGGGU...............GCGG...CUA.......CCGC.......AA........CAGGGUUGGCG.GG....UCAUAAGGGCGCGCGUCC........
env-18   UCCAGGAACCUCGUA....GGGU.....................................................GGGGGGU...............GCGG...UCAGG.....CCGC.......AA........CAGGGUUGGUG.GG....UCAUAAGGGCGCGCGUCC........
env-19   UCCAGGAACCUCGUA....GGGU.....................................................GGGGGGU...............GCGG...UUA.......CCGC.......AA........CAGGGUUGGCG.GG....UCAUAAGGGCGCGCGUCC........
env-20   ....................................................................................................................................................................................
env-21   UC.UGGGACCUCGUA....GGGU.....................................................GGGGGGU...............GCGG...CCGGA.....CCGC.......AA........CGCAACCGGUG.AG....U.........................
env-22   ....................................................................................................................................................................................
Tos-1-1  UC.CGGAACCUCGUA....GGGGGAAA.................................................CGG........................................GUAG...CA........GCGGCGGCGUGGC...CAAGCGAAGGCGGGAACCUGUC......
env-25   ....................................................................................................................................................................................
env-26   UC.CGGAACCUCGUA....GGGGGAAA.................................................CGG........................................GUAG...CA........GCGGCGGCGUGGC...CAAGCGAAGGCGGGAACCUGUC......
Tth-1-1  UC.CGGAACCUCGUA....GGGGGAAA.................................................GGGC.......................................CACGC..AA........GCGGCCAGGGC.....GUAGCGAAGGCGGGAAGGUGGC......
env-27   UC.CGGAACCUCGUA....GGGGGAAA.................................................GGGC.......................................CACGC..AA........GCGGCCAGGGC.....AUAGCGAAGGCGGGAAGGUGGC......
env-29   UCUGGGAACCUCGUA....GG.AGAA..................................................GGC........................................AACAAA.AA........ACGGACGGUAU.GG..G...........................
env-30   UCUGGGAACCUCGUA....GGGAGAAA.................................................GGCA.......................................ACAAAAAAA........CGGGACGGUAUGGG....UGUGAAGGCGGGAAGGCAAGC.....
Taq-1-2  UC.CGGAACCUCGUA....GAGGGAAG.................................................GGC........................................CUAGC..AA........GCGGG.CGGCGAGG..GCGUGAAGGCGGGAAGGCAAC.......
env-31   ....................................................................................................................................................................................
env-32   ....................................................................................................................................................................................
env-33   UC.CGGAACCUCGUA....GGGGGAAA.................................................GGC........................................GAGAAA.AA........ACGGGACGGUAUGG..G...........................
env-34   UC.CGGAACCUCGUA....GGGGGAAA.................................................GGC........................................CUAGC..AA........GCGGG.CGGCGAGG..GCGUGAAGGCGGGAAGGU.GC.......
env-35   UC.CGGAACCUCGUA....GGGGGAAA.................................................GGC........................................CUAGC..AA........GCGGG.CGGCGAGG..GCGUGAAGGCGGGAAGGU.GC.......
env-36   ....................................................................................................................................................................................
env-37   ....................................................................................................................................................................................
env-38   UC.CGGAACCUCGUA....GGGGGAAA.................................................GGC........................................CUA....GA........ACGC.GGGCGG.CGAGGGCGUGAAGGCGGGAAGGUGC.......
env-39   UC.CGGAACCUCGUA....GGGGGAAA.................................................GGC........................................CUAGA..AA........GCG.GGCGGCGAGG..G...........................
env-40   ....................................................................................................................................................................................
env-41   ....................................................................................................................................................................................
env-42   UG.UGGAACCUCGUA....GGGGGACA.................................................GGC........................................CUGGA..CA........GCGGCAGGGUUGGG....UGUGAAGGCGGGAAGGUGC.......
env-43   ....................................................................................................................................................................................
env-44   UU.UGGAACUUCGUA....GGGGGAAA.................................................GGGC.......................................GCA....AA........GCGGUCAGGGCGU.....AGCGAAGGCGGCAAGACAC.......
env-45   ....................................................................................................................................................................................
env-46   UG.UGGAACCUCGUA....GGGGGACAG................................................GCCA.......................................UA.....GA........CAGCAGCAGGGUUG..GGUGUGAAGGCGGCAAGACGC.......
Cte-1-1  U..GCGAACAUCG..CCU.CCCCGAAA.UC.......CAAG.........GAAA.GCC...GAAA....GGCUA..GCCGCUU...............UGU....UGAG.......ACA.......GU........GGCGC...........CACGUUGCG...................
Rpi-2-1  UU.UGGAAUCUCG..GGC.CGCUGAAA.ACC......UAAUG.......GGUGA.GAGG..GAACA..CCUCACU.GUUGU.................UGCU...GGA.......GGCG.......CA........GGC.GU..........GAAGUCCUGGCGCGCAGUUUGGCGU...
Rsp-1-1  UU.UGGAAUCUCG..GGC.CGCUGAAA.ACC......UAAUG.......GGUGA.GAGG..GAACA..CCUCACU.GUUGU.................UGCU...GGA.......GGCG.......CA........GGC.GU..........GAAGUCCUGGCGCGCAGUUUGGCGU...
Tin-1-1  UU.UGGAAUCUCG..GCC.CCCUGAAA.CCGC.....GAAA.......GCGG...GUAAG.CGUAG.CG.AC....GGUUUGAUUU.................................G......GA........GCGCGGCGGCUAAG..G...........................
         ....................................................................................................................................................................................
         ....................................................................................................................................................................................
         ....................................................................................................................................................................................
         ....................................................................................................................................................................................
         ...............>>>>>>>>.....<<<<<<<<<......>>>>>>>>>...<<<<<<.....>>>>>>....>>>>>>>>>>>>>>>>>>>>>.<<<<<<<.......>>>>>>>.........<<....>>>>>>>>>>>>>.>>..............................
         ...............222?22??.....22?220000......000022?22...22????.....????22....222???2????0?0???????.2222200.......0022222.........00....00222????????.??..............................
         ....................................................................................................................................................................................
         ....................................................................................................................................................................................
         UY-YGGAAYYUCG○-----●●●●●R●●-------------------------------------------------GR●●●○○-------------------------------------------RA--------R●●○○○○○○○-------○●●●●●●R●●●●●●●●●○○○-------
```

alignment positions 1441 · · · 1620

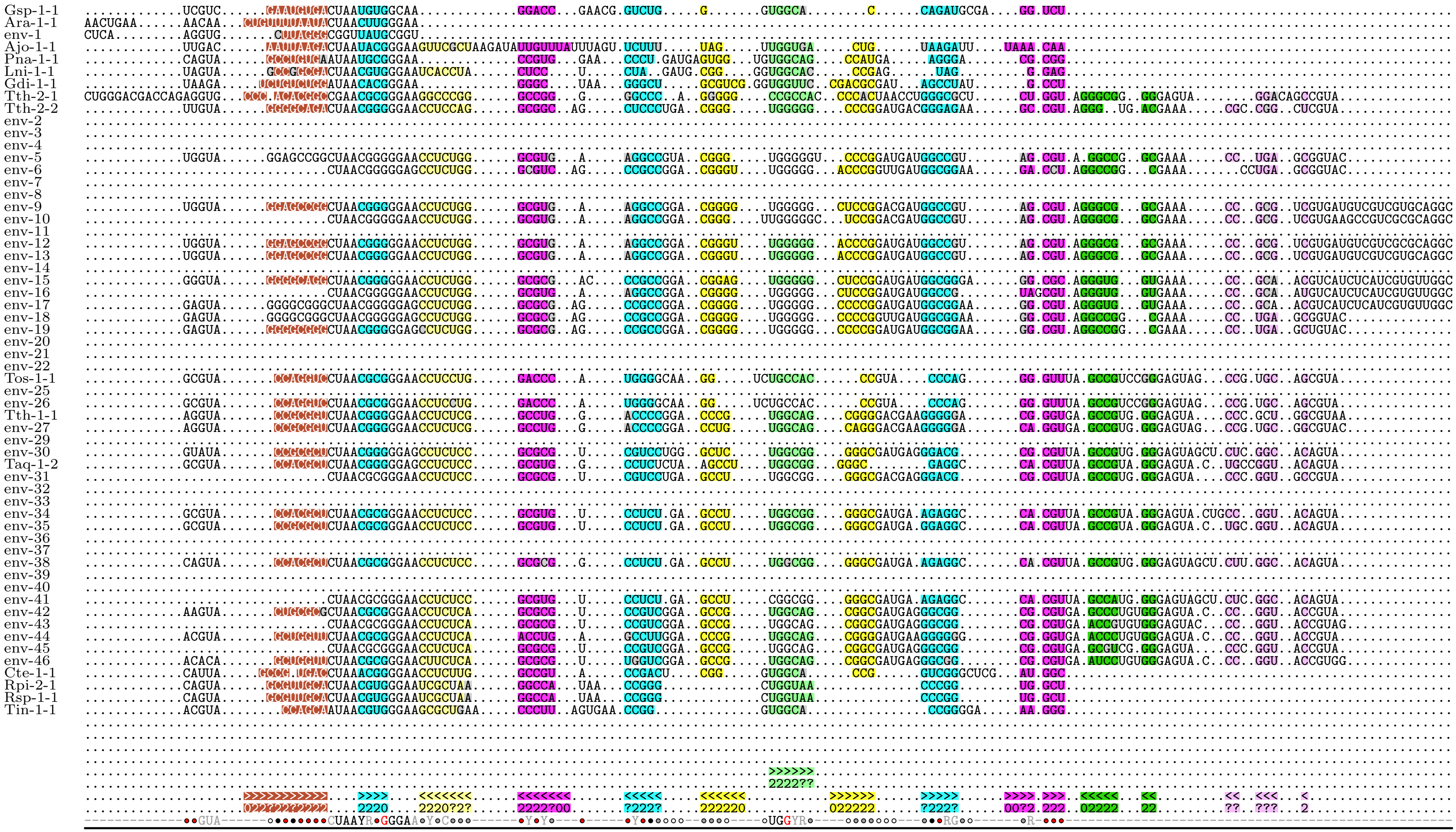

alignment positions 1621 · · · 1800

```
Gsp-1-1   ................................................................GAGAGCUUAACU.......................GAGUAGGUU.CCC...UGUG....GGGGCAUU............AGA.GCU.........GC.AGGGCAGU.......
Ara-1-1   ....................................................................................................................................................................................
env-1     ....................................................................................................................................................................................
Ajo-1-1   ....................................................GUUUUAGAGGAUGGGAGAUCGAUU.......................GAGUAAAG....................CGACUU.UUA......AGU.CGG.....GA..GC.AU.GAAUAU......
Pna-1-1   ................................................................GGGAGACUGGCUGAGCCGAGCAGCAAUGCGGAGCAAGUAGGG....................UUUGAA.GAG......GGG.GGCA.........GAACC.GAUGGA......
Lni-1-1   ..........................................................AGGUGAGGGAGACUGGCU.......................GAGUAGGAG.AUGC...GAAC...GCAUGUCGACCAGC.......AG.GGCA........GACCU.GACGGA......
Gdi-1-1   ................................................................GGGAGC.GGUCU.......................GAGUAGAG....................UGAAAA.UCU......GUG.CGGC........GG.UU.UAUCCGAA....
Tth-2-1   ....CCC...UGGAAC....GGG..GAG.GGC.CC.AA.CGAAAACC.CGUCCGAA..CCGGGCUGGGAGACCGGAU.......................CAGUAGGC....................UAA....ACC.CCGCCUGCUCCCGU...GGGG.GU.GC.GUCCA......
Tth-2-2   ....GG.GG.GAAGA...UC.CC..GAGGGGCUGG.CG.CGAUAAGUG..CCCGUG..CUGGAGGGGGAGACCAGGC.......................GAGUAGGU......................CA....CGC.GCGGG.GCUAGCGU..UCGGG.GUCGU.CCUCA.......
env-2     ....................................................................................................................................................................................
env-3     ....................................................................................................................................................................................
env-4     ....................................................................................................................................................................................
env-5     ....GGG...GAAA.....CCC.AGAGGGCU.CA.GGCACAUAAGC.GGCC.GCA..CCAGAGGGGGAGACCAGGC.......................AAGUAGGU......................G...UGC.CUUUGGGCUCCCAU...CGGG.GUACC.UGAUG.......
env-6     ....GGG...GAAA.....CCC.AUAGGGCU.CAUAG.AGAUAAG..CGGCCGCA..CCAGAGGGGGAGACCAGGC.......................GACUAGGC...................U.AA...ACC.GAAGCGGCUCCCAGUUUCCGGG.GUACC.UGAUG.......
env-7     ....................................................................................................................................................................................
env-8     ....................................................................................................................................................................................
env-9     UCUAGGGG..GAAA....CCCC..GAG.GGC.CC.GG.GGAUAAGU.CGCUCGCA..CCAGAGGGGGAGACCAGGC.......................GAAUAGGC...................U.AA...ACC.GAAGCGGCUCCCAC..ACGGG.GUACC.UGAUUGCUG...
env-10    UCUAGGGG..GAAA....CCCC..GAG.GGC.CC.GG.GGAUAAGU.CGCUCGCA..CCAGAGGGGGAGACCAGGC.......................GAAUAGGC...................U.AA...ACC.GAAGCGGCUCCCAC..ACGGG.GUACC.UGAUUGCUG...
env-11    ....................................................................................................................................................................................
env-12    UCUAGGGG..GAAA....CCCC..GAG.GGC.CC.GG.GGAUAAGU.CGCUCGCA..CCAGAGGGGGAGACCAGGC.......................GAAUAGGC...................U.AA...ACC.GAAGCGGCUCCCAC..ACGGG.GUACC.UGAUUGCUG...
env-13    UCUAGGGG..GAAA....CCCC..GAG.GGC.CC.GG.GGAUAAGU.CGCUCGCA..CCAGAGGGGGAGACCAGGC.......................GAAUAGGC...................U.AA...ACC.GAAGCGGCUCCCAC..ACGGG.GUACC.UGAUUGCUG...
env-14    ......................................................................CCAGGC.......................GAAUAGGC.....................UG...ACC.GAUGCGGCUCCCGU...CGGG.GUACC.UGUUG.......
env-15    CGUACGGG..GAAA....CCCA..GAG.GGC.CC.GG.GGAUAAGC.CACCCGAA..CCAGAGGGGGAGACCAGGC.......................GAAUAGGC...................U.AA...ACC.GAAGCGGCUCCCAU...CGGG.GUACC.UGAUG.......
env-16    CGUAUGGG..GAAA....CCCG..GAG.GGC.CC.GG.GGAUAAGC.CACCCGCA..CCAGAGGGGGAGACCAGGC.......................GACUAGGC...................U.AA...ACC.GAAGCGGCUCCCAU...CGGG.GUACC.UGAUG.......
env-17    CGCACGGG..GAAA....CCCG..GAGGGC..CC.GG.GGAUAAGC.CACCCGCA..CCAGAGGGGGAGACCAGGC.......................GAAUAGGC...................U.AA...ACC.GAAGCGGCUCCCAC..ACGGG.GUACC.UGAUUGAUG...
env-18    ....GGG...GAAA.....CCC.AGAGGGCU.CA.GGCACAUAAG..CGGCCGCA..CCAGAGGGGGAGACCAGGC.......................GAAUAGGC.....................UG...ACC.GAAGCGGCUCCCGU...CGGG.GUACC.UGCUG.......
env-19    ....GGG...GAAA.....CCC.AGAGGGCU.CA.GGCACAUAAG..CGGCCGCA..CCAGAGAGGGAGACCAGGC.......................GAAUAGGC...................AAAA...ACC.GACACGGCUCCCAAUUUCCGGG.GUACC.UGA..GAUG...
env-20    ....................................................................................................................................................................................
env-21    ....................................................................................................................................................................................
env-22    ....................................................................................................................................................................................
Tos-1-1   ....CC....GCAA.......GG..GAGGCGG.C..GG.CCAAAACGGCGGC.GUG..CAGGAGGGGGAGACCGGGC.......................CAGUACGA.......................A...GGC.GAAUG.GCC.UGUG...GGGG.GG.CC.CCGUA.......
env-25    ....................................................................................................................................................................................
env-26    ....CC....GCAA.......GG..GAGGCGG.C..GG.CCAAAACGGCGGC.GUG..CGAGAGGGGGAGACCGGGC.......................CAGUACGA.......................G...GGC.GAAUG.GCU.GCAG..UGGGG.GG.CC.CCGUA.......
Tth-1-1   ....GG.GGGCUUAGA.CCC.CC.CGAGGCGGU...GG.CGAAAACC.CGGC.GCG..CGAGAGGGGGAGACCGGGC.......................GAGUACGA.......................C...GGC.GAACG.GCU.GCUG...GGGG.GG.CC.CCGUA.......
env-27    ....GGAGG.C.CAGG..CCUCC.CGAGGCGG.C..GG.CGAAAACC.CGGC.GCG..CGAGAGGGGGAGACUGCCC.......................GAGUACGA.......................G...GGC.GGAA..GCU.GCGG...UGGG.GG.CC.CCGUA.......
env-29    ....................................................................................................................................................................................
env-30    ....GCCGG.CAUCGA..CCGGC..GAG.GGG.CC.AG.UGAAAACCAUGGU.GAG..GGAGAGGGGGAGACCGGGC.......................CAGUACGA.......................A...GGC.GAAUG.GCU.GCAG..UGGGG.GG.CC.CCGUA.......
Taq-1-2   ....CCGCGGCCUUGA.CCGCGG..GAG.GGA.CC.CA.GGAAAACUGCGGC.GUG..GGAGAGGGGGAGACCGGGC.......................CAGUACGA.......................G...GGC.GAAUG.GCU.GCAG..UGGGG.GG.CC.CCGUA.......
env-31    ....GCGCGGCGUUUGACCGCGC..GAG.GGG.CC.GG.CGAAAACCAUGGC.GUG..GGAGAGGGGGAGACCGGGC.......................CAGUACGA.......................G...GGC.GAAUG.GCU.GCGG..UGGGG.GG.CC.CCGUA.......
env-32    ....................................................................................................................................................................................
env-33    ....................................................................................................................................................................................
env-34    ....CCGCGGCGUUGA.CCGCGG..GAG.GGACCC.GG..GAAAACUGCGGU.GAG..GGAGAGGGGGAGACCGGGC.......................CAGUACGA.......................G...GGC.GAAUG.GCU.GCAG..UGGGG.GG.CC.CCGUA.......
env-35    ....CCGCGGCGUUGA.CCGCGG..GAG.GGA.CC.CG.GGAAAACUGCGGC.GUG..GGAGAGGGGGAGACCGGGC.......................CAGUACGA.......................G...GGC.GAAUG.GCU.GCGG..UGGGG.GG.CC.CCGUA.......
env-36    ....................................................................................................................................................................................
env-37    ....................................................................................................................................................................................
env-38    ....GCCGG.CAUCGA..CCGGC..GAC.GGG.CC.AG.UGAAAACCAUGGC.GAG..GGAGAGGGGGAGACCGGGC.......................CAGUACGA.......................G...GGC.GAAUG.GCU.GCAG..UGGGG.GG.CC.CCGUA.......
env-39    ....................................................................................................................................................................................
env-40    ....................................................................................................................................................................................
env-41    ....GCCGG.CAUCGA..CCGGC..GAG.GGG.CC.AG.UGAAAACCAUGGC.GAG..GGAGAGGGGGAGACCGGGC.......................CAGUACGA.......................C...GGC.GAAUG.GCU.GCAG..CGGAG.GA.CC.CCGUA.......
env-42    ....GC.GG.CAGCGA..CC.GCGCGAG.GGG.CC.GG.CGAAAACCGGGGU.GUG..UGAGAGGGGGAGACCUCCC.......................GAGUACG......................AG...GGC.GAAUG.GCU.GCAU.....GG.GGACU.CCGUA.......
env-43    ....GC.GG.CAGCGA..CC.GCGCGAG.GGG.CC.GG.CGAAAACCGGGGU.GUG..UGAGAGGGGGAGACCGGGC.......................CAGUACGA.......................A...AGC.GGA..GCU.UCGU...AGGG.GA.CU.CCGUA.......
env-44    ....GC.GG.CAGCGA..CC.GCGCGAG.GGG.CC.GG.CGAAAACCGGGGU.GUG..UGAGAGGGGGAGACCAG.C.......................CAGUACGA...........................GGC.GAAUG.GCU.GCAU...GGGG.GA.CU.CCUUU.......
env-45    ....GCGCGGCGUUGA.CCGUGU..GAG.GGG.CC.GG.CGAAAACCGGGGU.GCG..UGAGAGGGGGAGACCAGGC.......................CAGUACGA.......................G...GGC.GAAUG.GCU.GCGC...GGGG.GA.CU.CCAUG.......
env-46    ....GG.GG.CAGCGA..CC.GCGCGAG.GGG.CC.GG.CGAAAACCGGGGU.GUG..UGAGAGGGGGAGACCAGGC.......................CAGUACGA.......................G...GGC.GAAUG.GCU.GCGC...GGGG.GA.CU.CCUUG.......
Cte-1-1   .......................................................U.CAAGAGGGGGAGAUGUGAU.......................GAUUAGGG....................UUUGA..CCC......GUG.AUGC........GA.UA.CGACCGAA....
Rpi-2-1   .........................................................AU.GCGAGGGAGAGUGGUU.......................GAGUAGGGCCGCGUGGCGCAA.GCCACGU..A..............GCU.GUUU...GUCGCGA.UU.CGUCUGAA....
Rsp-1-1   .........................................................AU.GCGAGGGAGAGUGGUU.......................GAGUAGGGCCGCGUGGCGCAA.GCCACGU..A..............GCU.GUUU...GUCGCGA.UU.CGUCUGAA....
Tin-1-1   ..........................................................AAGCGUGGGAGA..............................................................................................................
          ....<<<<<<......>>>>>>.....>.>.>>.>>.......>>.>>>>>....>>>>>>>.....<<<<<<..<<<<<<<<...>>>>>>>>....<.<<.<<<<<<<....>>>>>>>.......<<<......<<<.<<<<...<<<<<.................
          ....222210......012222.....2.?.??.??.......22.22220....?2?0222.....222221..00???000...000???00....2.?2.?222000....000222?......?2?......2??.222?...??200.................
          ....................................................................................................................................................................................
          ....................................................................................................................................................................................
          ..................................................................................................................................................................>>.>>.>>..........
          ..................................................................................................................................................................??.??.??..........
          ....................................................................................................................................................................................
          ....................................................................................................................................................................................
          -----------------------------------------------------∘∘∘--∘∘∘G•R•GGGAGAYYRR•Y----------------------•AGUA•G•-----------------------∘•----∘∘∘-∘∘∘---G••-•••∘----∘∘∘∘-G•-YY-•••Y•-------
```

alignment positions 1801 · · · 1980

```
Gsp-1-1   ..............................................GGCUCU.......U......GUGUUAGCGGAAUCGG..GUGGCG..CCGAUUC.AAUAACGCUA.....................GACCGAAAGGUUAA...................................
Ara-1-1   ....................................................................................................................................................................................
env-1     ....................................................................................................................................................................................
Ajo-1-1   ..........................................UCGAGCGAAU.........AAAA.ACGUA.............AGCGUA.............UACAUGAGU....................AAUGAAAGGUUGAGCC................................
Pna-1-1   .............................................UGUUCCC.........GUU............................................AGU.....................ACCGAAAGCCUGGACC................................
Lni-1-1   .............................................UGUUCU..........GUU...................................................................UGGUGAAAGGCCGGAGC................................
Gdi-1-1   .............................................GCUGCAU.........GGAGAGGUUGCGUCAAACGGGGGUGGU..UCCUCGU.....GCAAUCAAAGU...................CUCGAAAGGACUAGGC................................
Tth-2-1   .........................................CCCUGCGGUGUGGGUUGGG.GG...ACGAUGC...........GCAU.............ACGUUAUAC......................GCCGAAAGUCCGGACCGGCAGA..........................
Tth-2-2   .........................................CCCGGCGGUGUCCCACGCG.GCG..ACGUGAA...........GGCGC.............UC.UGU........................ACCGAAAGCCUGGACCGGAUCA..........................
env-2     ....................................................................................................................................................................................
env-3     ....................................................................................................................................................................................
env-4     ....................................................................................................................................................................................
env-5     .........................................CCCGGUGGUGUCCGAAGGA.GCG..UCCCCUU...........GGCU..............ACGUGA........................ACCGAAAGCCUGGACCGGC..ACCCUGGGGG..CUUCCA.CCCCCAGG
env-6     .........................................CCCGGUGGUGU.CGUGGAG.GGA..CGUCAUC...........CCAGC............AACGAGG........................GCCGAAAGCCUGGACCGGU..ACCCUGGGGG..CUUCCA.CCCUCAAG
env-7     ....................................................................................................................................................................................
env-8     ....................................................................................................................................................................................
env-9     .........................................CCC.GUGGUGU.CGUGGAAGGGA..CGUCAUC...........CCAGC............AACGAGG........................GCCGAAAGCCUGGACG................................
env-10    .........................................CCCGGUGGUGU.CGUGGAG.GGA..CUUCAUC...........CCAGC............AACGAGG........................GCCGAAAGCCUGGACCGGC..ACCCUGGGGG..CUUCCA.CCCCCAGG
env-11    ....................................................................................................................................................................................
env-12    .........................................CCCGGUGGUGU.CGUGGAG.GGA..CGUCAUC...........CCAGC............AACGAGG........................GCCGAAAGCCUGGACCGGU..ACCCUGGGGG..CUUCCA.CCCUCAGG
env-13    .........................................CCCGGUGGUGU.CGUGGAG.GGA..CUUCAUC...........CCAGC............AACGAGG........................GCCGAAAGCCUGGACCGGC..ACCCUGGGGG..CUUCCA.CCCCCAGG
env-14    .........................................CCCGGCGGUGU.CGCGGAG.GG...CGACAUC...........UCGUC............UAUGCGG........................GCCGAAAGCCUGGACCGGC..ACCCCGGAG...UGUCCA..CUCCGGG
env-15    .........................................CCCGGUGGUGU.CGUGGAG.GGA..CGUCAUC...........UCAGC............AACGAGG........................GCCGAAAGCCUGGACCGG..ACCCCUGGAUGGAUUUCCA.CAUCCAGG
env-16    .........................................CCCGGUGGUGU.CGCGGAG.GGA..CGUCAUC...........UCAGC............AACGAGG........................GCCGAAAGCCUGGACCGGU..ACCCUGGGGG..CUUCCA.CCCUCAGG
env-17    .........................................CCCGGUGGUGU.CGUGGAG.GGA..CGUCAUC...........CCAGC............AACGAGG........................GCCGAAAGCCUGGACCGGU..ACCCUGGGGG..CUUCCA.CCCUCAGG
env-18    .........................................CCCGGCGGUGU.CGCGGAG.GG...CGACAUC...........UCGUC............UACGCGG........................GCCGAAAGCCUGGACCGGU..ACCCUGGGGG..CUUCCA.CCCUCAGG
env-19    .........................................CCCGGUGGUGU.CGUGGAG.GGA..CAUCGUC...........AUAUC............UACGA.G.A......................GCCGAAAGCCUGGACCGGC..ACCCUGGGGG..CUUCCA.CCCUCAGG
env-20    ....................................................................................................................................................................................
env-21    ....................................................................................................................................................................................
env-22    ....................................................................................................................................................................................
Tos-1-1   .........................................CCCCCACCGGU.GCACGAA.CCC..GGCCAGG...........GGAA.............CCUGGCC........................UAGGAAAGCCCGGACCGGCUUACCCGUGCGG..CGUCCA.CCGCACGG
env-25    ....................................................................................................................................................................................
env-26    .........................................CCCCCUGCGGU.GCACGAA.CCC..GCUGCCG...........CUGUG............CGGUGGC........................UAGGAAAGCCCGGACCGGCUUACCCUGGCU....UUCCA..GCCCAGG
Tth-1-1   .........................................CCCCUAGCGGU.GCGCGAA.CCC..GGCCCGC...........GUC..............GCGGGAU.G......................UAGGAAAGCCCGGACCGGCUUACCUCC........ACCA.....GGAG
env-27    .........................................CCCGCCGUGGU.G...GAA.CCU..GGCC..............UAAG................GGCC........................UAGGAAAGGGCAGACCGGCUUACCCUGGGG...CUUCCA..CCCCAGG
env-29    ....................................................................................................................................................................................
env-30    .........................................CCCCCUGCGGU.GCACGAA.CCC..GCUACCG...........CUACG............CGGUGGC........................UAGGAUAGCCCGGACCGGCUUGCCCUAGGAG..CUUCCACUUCCUAGG
Taq-1-2   .........................................CCCCCUGCGGU.GCACGAA.CCC..GCUACCG...........CUAUG............CGGUGGC........................UAGGAAAGCCCGGACCGGCUUACCCUGGCU....UUCCA..GCCCAGG
env-31    .........................................CCCCCUGCGGU.GCACGAA.CCC..GCUGCCG...........CUGUG............CGGUGGC........................UAGGAAAGCCCGGACCGGCUUACCCUGGCU....UUCCC..GCCCAGG
env-32    ....................................................................................................................................................................................
env-33    ....................................................................................................................................................................................
env-34    .........................................CCCCCUGCGGU.GCACGAA.CCC..GCUGCCG...........CUAUG............CGGUGGC........................UAGGAAAGCCCGGACCGGCUUACCCUGGCU...U.UCCA..GCCCAGG
env-35    .........................................CCCCCUGCGGU.GCACGAA.CCC..GCUGCC............GUAU..............GGCGGC........................UAGGAAAGCCCGGACCGGCUUACCCUAGGGG..CUUCCA.CCCCUAGG
env-36    ....................................................................................................................................................................................
env-37    ....................................................................................................................................................................................
env-38    .........................................CCCCCUGCGGU.GCACGAA.CCC............................................GN......................UAG...CCUAUGG...................................
env-39    ....................................................................................................................................................................................
env-40    ....................................................................................................................................................................................
env-41    .........................................CUCCCUGCGGU.GCACGAA.CCC..GCUGCC............GCAU..............GGCAGC.G......................UAGGAAAGCCCGGACCGGCUUACCCUGGCU...U.UCCA..GCCCAGG
env-42    .........................................CCU.GUGUGGU.GCACGAA.CCU..GGCCCCGC..........GAGGG...........G...............................................................................
env-43    .........................................CCCUACGCGGU.....GAA.CCU..GCUGCU............GUCC..............AGCAGC........................UAGGAAAGCCCGGACCGGCUUGCUCUGGGAG..CUUCCA.CUCCCAGA
env-44    .........................................GCCCGUGUGGU.GCACGAA.CCU..GGCCC.............GCGA...............GGGAU.G......................UAGGAAAGCCUGGACCGGCUUA.............CCCU.........
env-45    .........................................CCCUGUGUGGU.GCACGAA.CCU..GGCCCC............GCGA..............GGGGAU.G......................UAGGAAAGGGGGGACCGGCUACCCCUGGCU....UUCCG..GCCUGGG
env-46    .........................................CCCCGUGUGGU.GCACGAA.CCU..GGCCCC............GCGA..............GGGGAU.G......................UAGGAAAGCCUGGACCGGCUUACCCUGGCU...U.UCCA..GCCCAGG
Cte-1-1   .............................................GCAUC.C.........GGGGAGCUGUCUGAC........GAAGA.........GUCGG.CAGC.A...GUGGGUUUGGCGACCCGC.UCCGAAAGUCGCAAGC................................
Rpi-2-1   ........................................GCG.CAAACAGC.......A......GGC...............GCAA.................GCC.U.....................GUCCGAAAGACUGCGGC................................
Rsp-1-1   ........................................GCG.CAAACAGC.......A......GGC...............GCAA.................GCC.U.....................GUCCGAAAGACUGCGGC................................
Tin-1-1   ....................................................................................................................................................................................
          ....................................................................................................................................................................................
          ....................................................................................................................................................................................
          ....................................................................................................................................................................................
          ....................................................................................................................................................................................
          ........................................>>>>>>>>>>>>.........>>>..<<<<<<<<<<<<<<<<<<......>>>>>>>>>>>>>>>>>>.....<<<<<<<....>>>>>>>>>.>....>>>>>>...<<<...<<<<<<<<<.........>>>>>>>>
          ........................................002???222??2.........?2?..2?22?2?????0221000......0001220?????2?22?2.....0000000....00000002?.2....122222...11?...022221221.........12212222
          ....................................................................................................................................................................................
          ....................................................................................................................................................................................
          -----------------------------------------○○○○●●●●●●Y-----○○○-●●●--●●●●●○○-----------●●●●--------------○○○●●●-○----------------------●●YGAAAG●YYGRA●CGGY○○ACYY●●○○○---○○YCC●--○○○●●RR
```

alignment positions 1981 · · · 1999

```
Gsp-1-1   ...................
Ara-1-1   ...................
env-1     ...................
Ajo-1-1   ...................
Pna-1-1   ...................
Lni-1-1   ...................
Gdi-1-1   ...................
Tth-2-1   .UGGACCUC..GGCACCAC
Tth-2-2   .G.GACCUC..AGCGCCAC
env-2     ...................
env-3     ...................
env-4     ...................
env-5     GCCGGCCUC..AGUGCCAC
env-6     GCCGGCCUU..GGCGCCAC
env-7     ...................
env-8     ...................
env-9     ...................
env-10    GCCGGCCUC..AGUGCCAC
env-11    ...................
env-12    GCCGGCCUU..GGCACCAG
env-13    GCCGGCCUC..AGUGCCAC
env-14    GCCGGCCUU..AGCACCAC
env-15    GCCGGCCUC..GGCACA.C
env-16    GCCGGCUUC..GGCACCAC
env-17    GCCGGCCUU..GGCACCAG
env-18    GCCGGCCUC..AGUGCCAU
env-19    GCCGGCUUUA.GGCACUGC
env-20    ...................
env-21    ...................
env-22    ...................
Tos-1-1   GG..GCCUC..GGCGCCAA
env-25    ...................
env-26    GG..GCCUC..GGCACC.C
Tth-1-1   GG..GCCUC..GGCACCAC
env-27    GG..GCCUC..GGCACCGC
env-29    ...................
env-30    GG..GCCUC..GGCGCCAA
Taq-1-2   GG..GCCUC..GGCGCCAA
env-31    GG..GCCUC..GGCACC.C
env-32    ...................
env-33    ...................
env-34    GG..GCCUC..GGCACC.C
env-35    GG..GCCUC..GGCACC.C
env-36    ...................
env-37    ...................
env-38    ...................
env-39    ...................
env-40    ...................
env-41    GG..GCCUC..GGCGCCAA
env-42    ...................
env-43    GG..GCCUC..GGCGUCAC
env-44    .GG.GUUUC..CA......
env-45    GG.GGCCUC..GGCGUCAC
env-46    GGG.GCCUC..GGCGUCAC
Cte-1-1   ...................
Rpi-2-1   ...................
Rsp-1-1   ...................
Tin-1-1   ...................
          ...................
          ...................
          .............>>>>>>
          .............122012
          >...>>>............
          0...?11............
          ...................
          ...................
          G•-∘GCCUC--RGCRCCAC
```
